# Purinergic P2Y2 and P2X4 Receptors Are Involved in the Epithelial-Mesenchymal Transition and Metastatic Potential of Gastric Cancer Derived Cell Lines

**DOI:** 10.3390/pharmaceutics13081234

**Published:** 2021-08-11

**Authors:** Mauricio Reyna-Jeldes, Erwin De la Fuente-Ortega, Daniela Cerda, Erandi Velázquez-Miranda, Katherine Pinto, Francisco G. Vázquez-Cuevas, Claudio Coddou

**Affiliations:** 1 Departamento de Ciencias Biomédicas, Facultad de Medicina, Universidad Católica del Norte, Larrondo 1281, Coquimbo 1781421, Chile; mauricio.reyna.jeldes@gmail.com (M.R.-J.); edelafuente@ucn.cl (E.D.l.F.-O.); daniela.cerda.barraza@gmail.com (D.C.); k.pintoirish@gmail.com (K.P.); 2 Millennium Nucleus for the Study of Pain (MiNuSPain), Santiago 8331150, Chile; 3 Núcleo Para el Estudio del Cáncer a Nivel Básico, Aplicado y Clínico, Universidad Católica del Norte, Larrondo 1281, Coquimbo 1781421, Chile; 4 Departamento de Neurobiología Celular y Molecular, Instituto de Neurobiología, Universidad Nacional Autónoma de México, Querétaro 76230, Mexico; erandivelazquez@gmail.com (E.V.-M.); fvazquez@comunidad.unam.mx (F.G.V.-C.)

**Keywords:** gastric cancer, purinergic receptors, cell migration, proliferation, TEER

## Abstract

Gastric cancer (GC) is a major health concern worldwide, presenting a complex pathophysiology that has hindered many therapeutic efforts so far. In this context, purinergic signaling emerges as a promising pathway for intervention due to its known role in cancer cell proliferation and migration. In this work, we explored in more detail the role of purinergic signaling in GC with several experimental approaches. First, we measured extracellular ATP concentrations on GC-derived cell lines (AGS, MKN-45, and MKN-74), finding higher levels of extracellular ATP than those obtained for the non-tumoral gastric cell line GES-1. Next, we established the P2Y2 and P2X4 receptors (P2Y2R and P2X4R) expression profile on these cells and evaluated their role on cell proliferation and migration after applying overexpression and knockdown strategies. In general, a P2Y2R overexpression and P2X4R downregulation pattern were observed on GC cell lines, and when these patterns were modified, concomitant changes in cell viability were observed. These modifications on gene expression also modified transepithelial electrical resistance (TEER), showing that higher P2Y2R levels decreased TEER, and high P2X4R expression had the opposite effect, suggesting that P2Y2R and P2X4R activation could promote and suppress epithelial-mesenchymal transition (EMT), respectively. These effects were confirmed after treating AGS cells with UTP, a P2Y2R-agonist that modified the expression patterns towards mesenchymal markers. To further characterize the effects of P2Y2R activation on EMT, we used cDNA microarrays and observed that UTP induced important transcriptional changes on several cell processes like cell proliferation induction, apoptosis inhibition, cell differentiation induction, and cell adhesion reduction. These results suggest that purinergic signaling plays a complex role in GC pathophysiology, and changes in purinergic balance can trigger tumorigenesis in non-tumoral gastric cells.

## 1. Introduction

Cancer, or malignant neoplasia, is a disease that involves several impairments among signaling pathways that regulate crucial cellular functions like cell growth and proliferation [1]. This accumulation of cell disturbances can be enhanced by several lifestyle risk factors, such as obesity, smoking, and alcohol consumption, or exposition to environmental factors like UV light, ionizing radiation, and chemical compounds known as carcinogens [2]. This large number of possible causes, its unpredictable onset, and the high variety of tissues and cell types that can be affected, make cancer a major health concern. Nowadays, and according to WHO assessments performed in 2011, cancer-related deaths are the highest worldwide, surpassing those linked to cardiovascular diseases. Overall, there were 14.1 million new cases and 8.2 million deaths associated with cancer in 2012, being lung (1.6 million deaths), liver (745,000 deaths), and stomach cancer (723,000 deaths) among the deadliest around the world [3]. About the latter, gastric cancer (GC) had a global incidence of 1,089,103 cases in 2020, being distributed similarly between males and females, with higher rates of incidence and mortality in Eastern and Western Asia (Japan, Korea, and Iran), and Latin America (Ecuador and Costa Rica) [4]. In Chile, GC is the leading cause of cancer-related deaths, with incidence and mortality rates of 15.6 and 13.8 per 100,000 inhabitants, respectively, according to IARC [5].

Understanding cancer pathophysiology is a frequent strategy to identify prospective therapeutic targets or clinical biomarkers. Intervening on cellular processes which are hallmarks on cancer onset, like cell proliferation, metabolism, cell death, genome stability, angiogenesis, inflammation, cell migration, and immune evasion [6], could lead to more effective, selective, and safer antineoplastic effects, which could be used as feasible treatment alternatives for highly-resistant tumors to traditional chemotherapeutics [7]. Among the plethora of cell mediators that play crucial roles in cancer progression, extracellular nucleotides, like adenosine triphosphate (ATP) and uridine triphosphate (UTP), and nucleosides like adenosine, contribute to several cell functions using a route known as purinergic signaling. It has been proposed that this pathway is involved in several processes such as immunosuppression, migration, cell survival/death balance, and differentiation, among other host/tumor interactions [8]. All these purinergic-associated functions are driven by the interaction between nucleotides and their respective purinergic receptors located in the cell membrane. These receptors are classified into two groups: ligand-gated ion channels (P2X) and G protein-coupled receptors (P2Y and P1). P1 receptors are exclusively activated by adenosine, and P2Y receptors (P2YR) are a receptor family triggered by a series of nucleotides like ATP, ADP, UTP, and other nucleotide-based synthetic molecules. On the other hand, P2X receptors (P2XR) are ATP-gated ion channels permeable to Ca^2+^, Na^+^, and K^+^, which are divided into seven different subtypes: P2X1 to P2X7 [9]. The involvement of these receptors has been described in several tumors affecting different tissues and organs like bladder, skin, prostate, pancreas, colon, ovary, and breast; where, in most cases, P2YR are involved in cell proliferation signaling networks, while P2XR mediate responses among differentiation and viability circuits [8,10]. Regarding GC, studies have revealed a significant increase in P2Y2R expression in biopsy samples from GC patients submitted to gastrectomy [11], whose overexpression has been widely associated with an increase in cell proliferation, tumor growth, and metastasis [12,13]. Additionally, our group confirmed this proliferative role associated with P2Y2R on GC cell lines, finding also a regulatory role for P2X4R [14].

Epithelial-mesenchymal transition (EMT) is a key process during development and differentiation that involves the loss of epithelial identity and acquisition of mesenchymal characteristics by the expression of corresponding proteins [15]. In cancer, EMT is highly deregulated and EMT-transcription factors exert important roles in all cancer stages, including initiation, primary tumor growth, invasion, dissemination, metastasis, colonization, and therapy resistance as well [16]. Moreover, EMT is also related to the generation of cancer stem cells (CSCs), a small subpopulation of tumor cells with capabilities of self-renewal, differentiation, and tumorigenicity, that make them responsible for tumor relapse and chemotherapy resistance [17]. In general, EMT results in the loss of typical proteins that constitute the epithelial phenotype, such as E-cadherin, β-catenin, claudin-1, and occludin, and acquisition of mesenchymal markers such as N-cadherin, vimentin, and fibronectin [18]. Several proteins have been described to play a role in EMT and, interestingly, it has been suggested a role for extracellular nucleotides and purinergic signaling in EMT, acting through P2YRs, P2XRs, and P1Rs [18]. Regarding cancer, studies have been performed on several cancer-derived cell lines, reporting different roles in cell proliferation for P2X5R, P2X7R, P2Y1R, P2Y2R, and P2Y11R in prostate cancer; P2X7R and P2Y2R in ovarian cancer; P2X7R, P2Y2R, P2Y6R, P2Y11R, and P2Y12R in breast cancer; and P2X4R, P2X7R, P2Y2R and P2Y6R in lung cancer [19]. However, there is no further information about the involvement of P2Y2R and P2X4R in cell motility, differentiation, or EMT. In this investigation, we focused to characterize the P2Y2R and P2X4R expression profile in GC cell lines from different tumor stages and grades to evaluate differences among them, and to establish P2Y2R and P2X4R contribution on GC proliferation and migration using molecular intervention strategies. To further investigate the role of purinergic signaling in EMT, we performed microarray analysis on GC-derived cells after UTP stimulation.

## 2. Materials and Methods

### 2.1. Cell Culture

All the experiments were performed on human gastric cell lines corresponding to the non-tumoral epithelium (GES-1), primary adenocarcinoma (AGS), and moderately (MKN-74) and poorly (MKN-45) differentiated metastatic adenocarcinoma, which were acquired from ECACC (AGS cell number: 89090402; Porton Down, Salisbury, UK), Riken Cell Bank (MKN-45 cell number: RCB1001, and MKN-74 cell number: RCB1002; Tsukuba, Ibaraki, Japan) and Beyotime Biotechnology (GES-1 cell number: C6268; Shanghai, China). These cells lines were cultured in vented cap flasks with DMEM, F12K, and RPMI 1640 culture media (all from Corning, Corning, NY, USA) for GES-1, AGS, and MKN-45 and MKN-74, respectively. These media were supplemented with 10% fetal bovine serum (Biological Industries, Beit HaEmek, Israel), 100 U/mL penicillin, and 100 μg/mL streptomycin (Corning, Corning, NY, USA). Cell lines were maintained in a humidified incubator at 37 °C with 5.0% CO_2_, using them for experiments up to their twentieth passage, being replaced with a new cryopreserved culture after reaching that number.

### 2.2. Extracellular ATP Measurements

For extracellular ATP assessment, GES-1, AGS, MKN-74, and MKN-45 were seeded in 96-well white opaque plates (Thermo Fisher Scientific, Waltham, MA, USA) considering an initial seeding of 3000 cells/well to achieve a 60–80% confluence after a 48 h incubation at 37 °C + 5.0% CO_2_. After incubation, ATP assessments were performed with a chemiluminescent ATP Determination Kit (Molecular Probes, Eugene, OR, USA) following the manufacturer’s guidelines for ATP standard curves, reagents concentration, and a 20 min incubation at 37 °C to promote the enzymatic reaction prior to measurement. To rule out the ATP contribution by supplemented culture media, ATP standard curves were performed using each one of these media as a solvent, creating 0 to 2000 nM relative ATP gradients, where 0 nM ATP corresponds to the amount of ATP available on supplemented culture medium. Relative Light Units (RLU) measurements were performed using a NOVOstar Plate Reader (BMG Labtech, Ortenberg, Germany) and ATP concentrations were calculated by interpolating RLU values on their respective standard curves. For statistical analysis, ATP measurements from each GC cell line were compared between them and with those obtained for GES-1 non-tumoral cells.

### 2.3. Real-Time qPCR Experiments to Determine Purinergic Receptors and EMT-Related Gene Expression

To guarantee quality and reproducibility in our experiments, MIQE Guidelines were considered in every aspect of the following methodology [20]. GC and non-tumoral gastric cell lines were seeded on 60 mm culture dishes at a proportion of 50,000 cells/dish to achieve a 60–80% confluence after a 24 h incubation at 37 °C + 5.0% CO_2_. To assess the effects of purinergic stimulation on the expression of epithelial and mesenchymal markers, 100 μM UTP (Sigma-Aldrich, St. Louis, MO, USA) was added to each GC and non-tumoral cell line, and these plates were incubated for 24 or 48 h. After incubation, mRNA was extracted using TRIzol reagent (Invitrogen, Waltham, MA, USA) according to the manufacturer’s protocols. Yield and purity ratios (260 nm/280 nm and 260 nm/230 nm) were established using a NanoDrop One^®^ microvolume spectrophotometer (Thermo Fisher Scientific, Waltham, MA, USA). Reverse transcription was performed using an Affinity Script qPCR cDNA Synthesis Kit (Agilent Technologies, Santa Clara, CA, USA), employing 1.0 μg of extracted mRNA, considering a no retrotranscriptase control (-RT) for each sample. For qPCR reactions, Brilliant II SYBR^®^ Green QPCR Master Mix (Agilent Technologies, Santa Clara, CA, USA) was used considering 50 ng of cDNA, and 300 nM as final primer concentration for each one of the studied genes. In every situation, P2Y2R, P2X4R, tight junction protein-1 (ZO-1), E-cadherin (CDH-1), and vimentin (VIM) were defined as target genes, and B2M was used as a referential gene. Primer design was performed using Primer-Blast, focusing on obtaining an amplicon size of 50–250 bp and an annealing/extension temperature of 60 °C (Supplementary Table S1). The thermal protocol used for all of these experiments starts with an initial denaturation step of 95 °C for 10 min, followed by an amplification phase of 40 cycles consisting of 30 s at 95 °C and 60 s at 60 °C, detecting fluorescence at the end of each cycle. After amplification, melting curves were performed on the amplified products, incubating them at 95 °C for 60 s, ramping down to 55 °C and then increasing temperature to 95 °C by a rate of 0.2 °C/s, measuring fluorescence data continuously. For additional identity control, 10 μL of each amplified sample was used for amplicon size detection in GelRed (Biotium, Fremont, CA, USA)-stained 1.5% agarose gels. To calculate relative gene expression, efficiency curves were performed for each gene, choosing the most proper calculation method between the one proposed by Livak and Schimittgen or Pfaffl according to the differences in the amplification efficiency of each one of the analyzed genes [21,22]. P2X4R and P2Y2R experiments were analyzed using GES-1 cells as basal expression control, and statistical comparisons were made between GC-derived cell lines, and between GES-1 cells and GC cell lines. For EMT-related genes, statistical analysis was performed using the UTP-untreated condition of each cell line as control.

### 2.4. P2Y2R and P2X4R Molecular Interventions

*Plasmids and siRNA.* As overexpression (OE) strategies, plasmids containing human P2Y2R and P2X4R were used. The P2Y2R-EGFP plasmid was designed by P2Y2R PCR amplification using a hP2Y2R-pcDNA3.1+ construct (Missouri S&T cDNA Resource Center) as a template, and a primer pair with *Bgl*II restriction sites and no stop codon sequence (Fw: 5′-TACCGGACTCAGATCTCGGATCCACCATGGCAG-3′; Rv: 5′-CTTGAGCTCGAGATCTGACAGCCGAATGTCCTTAG-3′). After amplification, the P2Y2R fragment was digested, purified, and cloned into the *Bgl*II restriction site of the pEGFP-N1 vector (Clonetech, Mountain View, CA, USA) using the In-Fusion Cloning kit (Takara Bio, San José, CA, USA). Proper fusion of the hP2Y2R pEGFP-N1 cDNA was confirmed by sequencing. Expression of P2Y2R-EGFP was confirmed by western-blot and immunofluorescence (data not shown). P2X4R pIRES2-EGFP plasmid was kindly provided by Dr. Stanko Stojilkovic and was designed according to previous reports [23]. For knockdown (KD) experiments, Silencer Select siRNA (Thermo Fisher Scientific, Waltham, MA, USA) was used for both P2Y2R (siRNA ID: s9965) and P2X4R (siRNA ID: s9955).

*Transient transfection protocol.* GES-1 and GC-derived cell lines were seeded in 24-well plates (Corning, Corning, NY, USA) to achieve a 50–60% confluence after a 24 h incubation at 37 °C + 5.0% CO_2_. After cell adhesion, cells were transfected for 6 h using Lipofectamine 2000 and Lipofectamine RNAiMax (Thermo Fisher Scientific, Waltham, MA, USA) for OE and KD experiments, respectively, according to manufacturer’s guidelines for cell transfection in 24-well plates. For OE experiments, 500 ng of cDNA were used, and 5 pmol of siRNA were employed for each KD situation. These transfection parameters for OE and KD experiments were optimized assessing P2Y2R and P2X4R mRNA levels via qPCR on AGS cells transfected for 6, 12, and 24 h, using as control conditions AGS cells transfected with empty pEGFP-N1 and pIRES2-EGFP vectors for overexpression experiments, and Silencer Select siRNA Negative Control (siRNA ID: 4390843; Thermo Fisher Scientific, Waltham, MA, USA) for knockdown experiments. These results are summarized in Supplementary Figure S1.

### 2.5. Cell Viability Experiments

GES-1, AGS, MKN-74, and MKN-45 were seeded in 24-well plates (Corning, Corning, NY, USA) considering an initial seeding of 3000 cells/well to achieve a 40–50% confluence after a 24 h incubation at 37 °C + 5.0% CO_2_. After cell adhesion, wells were transfected for 6 h using the same protocols, strategies, and constructs described for transient transfections. 48 h post-transfection, a 70 μM Resazurin (Sigma-Aldrich, St. Louis, MO, USA) solution was applied to each well, and fluorescence measurements were executed after a 6 h incubation at 37 °C + 5.0% CO_2_. Relative Fluorescent Units (RFU) determinations were performed using a NOVOstar Plate Reader (BMG Labtech, Ortenberg, Germany) and cell proliferation was calculated by comparing each experimental condition with its respective control of non-transfected cells.

### 2.6. Transepithelial Electrical Resistance (TEER) Measurements

GES-1, AGS, MKN-74, and MKN-45 were seeded in 12-well Transwell plates with 8.0 μm pore size inserts (Corning, Corning, NY, USA) considering an initial seeding of 150,000 cells/well to achieve a 60–80% confluence after a 24 h incubation at 37 °C + 5.0% CO_2_. After this incubation, OE and KD procedures were performed as previously described. After 1, 3, 5, and 7 days post-transfection, transepithelial electrical resistance (TEER) measurements were performed using an EVOM2 Epithelial Voltohmeter with an STX2 electrode (World Precision Instruments, Sarasota, FL, USA). TEER was calculated according to Kim et al. [24], considering a growth area of 1.12 cm^2^ and the non-transfected wild-type (WT) condition of each cell line as a control situation for statistical analysis.

### 2.7. Immunofluorescence

GES-1 and AGS cells were seeded on 24-well plates with glass coverslips to obtain a 60–80% confluence after a 24 incubation. After this, cells were fixed and processed for immunofluorescence using the same protocol previously described in our group [14]. After permeabilization and blocking, cells were incubated with a 1:200–1:400 dilution of ready-to-use primary antibodies against vimentin or Pan-CK (RM-9120 and MS-343, respectively; Thermo Fisher Scientific, Waltham, MA, USA) in a humidified chamber overnight at 4 °C. Following incubation, cells were washed three times with PBS and incubated with a 1:500 dilution of a proper Alexa Fluor 488-conjugated goat anti-mouse or anti-rabbit secondary antibody (A-11001 and A-11008, respectively; Thermo Fisher Scientific, Waltham, MA, USA), together with nuclei stain Hoechst 33342 (1:2000) (Sigma-Aldrich, St. Louis, MO, USA) for 1 h at RT. After washing with PBS, coverslips were mounted with Prolong (Thermo Fisher Scientific, Waltham, MA, USA). Single focal images were taken using a Zeiss LSM 800 confocal microscope (Carl Zeiss, Heidelberg, Germany) with a planapochromat 63×/1.46 oil objective. Images were acquired as 16-bit, 1024 × 1024 pixels, avoiding signal saturation, pinhole adjusted to 1 Airy unit, gain between 630 and 770 V, and laser power ranging from 1.48 to 21.32% for the 488 nm laser. Acquired images were processed generating regions of interest (ROIs) using ZEN Imaging Software 3.4 (Carl Zeiss, Heidelberg, Germany), and figure composition was performed using Adobe Photoshop CS6 (Adobe Systems, San José, CA, USA). Signal colocalization was quantified using the Manders’ coefficients method as described previously [25,26]. To establish the area of nuclei (Hoechst 33342) occupied by Vimentin or cytokeratins (Pan-CK, Alexa Fluor 488), we measured the pixel number of markers (Vimentin or Pan-CK) that colocalized with nuclei signal and divided these values by the total pixels obtained for each marker.

### 2.8. Microarray Studies

Microarrays were performed in the Microarray Unit at the Institute of Cellular Physiology (UNAM, CDMX, Mexico). AGS cells were seeded on 100 mm culture plates to achieve a 70–80% confluence after a 24 h incubation. Subsequently, a control condition with culture medium and another with a 100 μM UTP treatment were maintained for 24 or 48 h. Additionally, a final condition with AGS cells incubated with 5 U/mL Apyrase for 48 h was added, after which mRNA was extracted and purified using the Trizol method following manufacturer’s instructions (Thermo Fisher Scientific, Waltham, MA, USA). Then, cDNA synthesis was performed with 10 μg of total mRNA and using the First-Strand cDNA labeling kit (Thermo Fisher Scientific, Waltham, MA, USA) to incorporate both dUTP-Alexa555 or dUTP-Alexa647 probes. Fluorescence emission was analyzed at 555 and 650 nm for Alexa555 and Alexa647, respectively. Labeled cDNA was then hybridized against an array of 1920 transcripts related to human cancer. This array contained a 45-mer oligo library and was synthesized by MWG Biotech, USA. Array images were acquired and quantified using GenePix 4100A software (OMICtools, RRID:SCR_002250; Molecular Devices, San José, CA, USA). Values of the mean density of both fluorescent probes and mean background were calculated, and microarray data analysis was performed with freeware (genArise, http://www.ifc.unam.mx/genarise/, accessed on 8 November 2019) (RRID:SCR_001346) developed in the Computing Unit of the Institute of Cellular Physiology (UNAM, Mexico) GenArise identifies different gene expression patterns by calculating an intensity-dependent Z-score, where z stands for the number of standard deviations a datapoint is from the mean. Considering this criterion, elements with a z-score > 1.5 standard deviations were defined as transcript genes differentially expressed. To perform the bioinformatics analysis, and to identify the physiological roles of those genes regulated by UTP at both time conditions, we used the STRING database to visualize the interactions between the sets of genes that were up or downregulated in each condition as networks. The interactions shown in each network included known and predicted associations. Data were deposited in ArrayExpress-EMBL-EBI (accession number: E-MTAB-10742 for UTP experiment and E-MTAB-10841 for apyrase experiment). STRING database allows an enrichment analysis with two indicators for the functional pathways that genes can be annotated to, which include: Count in Network, which refers to the number of genes in our network annotated to a Gene Ontology (GO) term in relation with the total number of genes of the GO term; and False Discovery Rate (FDR), which describes the significance of the enrichment with a *p*-value obtained with the Benjamini–Hochberg Procedure considering the information from the sources available in STRING [27]. For the styling and manipulation of the information as networks, we used the software platform Cytoscape (Cytoescape Team, San Diego, CA, USA) [28], from which the Shared network from the 24 and 48 h stimulation conditions were obtained.

### 2.9. Statistical Analysis

**Figure 1:**
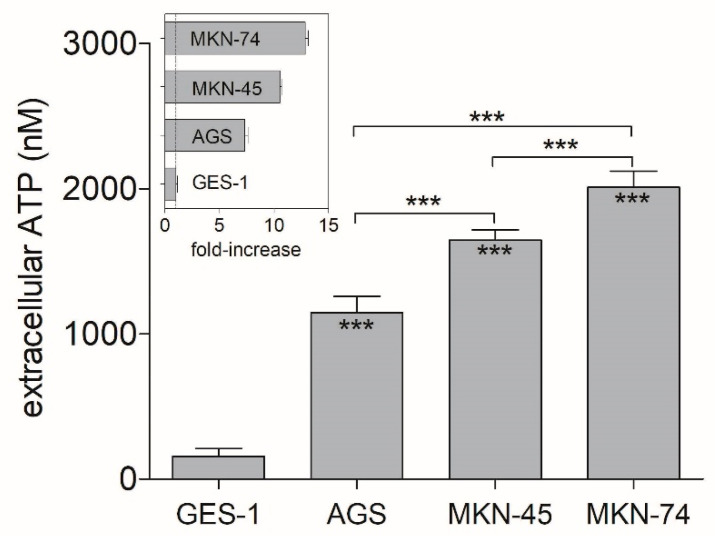
Extracellular ATP concentrations were found on non-tumoral and GC cell lines under basal conditions. ATP measurements were performed after a 48 h incubation of each cell line at normal culture conditions. Luciferin/Luciferase reactions were incubated for 20 min before reading. Inset shows fold-increase in extracellular ATP normalized against GES-1 cells. *** *p* < 0.00, 1-way ANOVA and Tukey’s post hoc test. For statistical analysis, comparisons were done between each GC cell line and the ATP concentrations found for GES-1 cells and comparing among GC-derived cell lines (*n* = 6).

**Figure 2:**
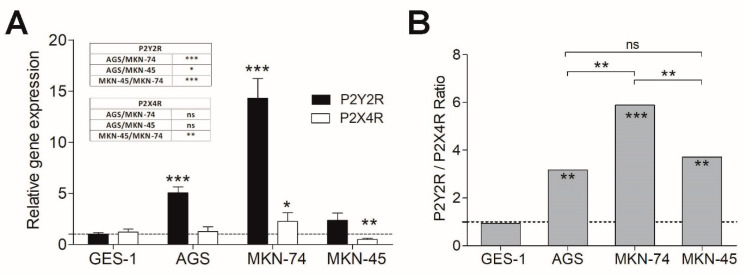
P2Y2R and P2X4R expression analysis on GC cell lines. (**A**) mRNA levels obtained by qPCR using the 2-ΔΔC_q_ method. For proper quantification, hB2M was chosen as a referential gene and GES-1 cells were used as a basal condition. (**B**) P2Y2R/P2X4R relative gene expression ratio among the studied GC-derived cell lines. *ns* *p* ≥ 0.05; * *p* < 0.05; ** *p* < 0.01; *** *p* < 0.001, 1-way ANOVA and Tukey’s post hoc test, comparing among GC cell lines, and between each GC-derived cell line and the non-tumoral GES-1 condition (*n* = 4).

Stata 14.1 (StataCorp, College Station, TX, USA) was used as statistical analysis software and the graphs were made using GraphPad Prism 6.01 (GraphPad Software, San Diego, CA, USA). To evaluate the normal distribution and homoscedasticity among our variables, Shapiro–Wilk and Bartlett tests were respectively performed. Parametric analysis was performed by Student’s *t*-test, considering Mann–Whitney as a non-parametric alternative. For the results of Figure 1 and Figure 2, One-way analysis of variance (ANOVA) and Tukey’s post hoc test for multiple comparisons were performed. For all cases, significance level was established at α = 0.05, and defining 0.01 ≤ *p* < 0.05 as statistically significant (*), 0.001 ≤ *p* < 0.01 as highly significant (**), and *p* < 0.001 as whole level significance (***).

## 3. Results

### 3.1. Extracellular ATP Concentrations on Non-Tumoral and GC Cell Lines

First, we measured the ATP concentrations released by our studied GC-derived cell lines (AGS, MKN-45, and MKN-74), to compare them with the non-tumoral epithelium-derived cell line (GES-1), used as control. After discarding the ATP contribution made by supplemented culture medium, the lowest extracellular concentrations were found on GES-1 cells (≈150 nM), which can provide a hint of the availability of the purinergic ligand on a non-tumoral condition. Conversely, all the GC-derived cell lines showed extracellular ATP concentrations that were 6–14 fold higher (≈1000–2000 nM) than those observed on control GES-1 cells (Figure 1). MKN-74 cells exhibited the highest extracellular ATP levels (≈2000 nM), which were significantly higher than those obtained for the other GC-derived cell lines. A similar trend was found for MKN-45, which showed higher extracellular ATP levels (≈1500 nM) than those observed on AGS cells (≈1000 nM, Figure 1). These results suggest that there is an important increase in ATP release on GC-derived cell lines.

### 3.2. P2Y2R and P2X4R Expression Profile in Non-Tumoral and GC-Derived Cell Lines

Next, we characterized the expression of P2Y2R and P2X4R on GES-1, AGS, MKN-45, and MKN-74 cell lines using qPCR and the Livak and Schmittgen method (2ΔΔCq). Our results showed a P2Y2R overexpression in all three GC-derived cell lines as compared to control GES-1 cells (Figure 2A). However, MKN-45 cells exhibited a very modest increase in P2Y2R (2.36 fold) in comparison with AGS and MKN-74 cells (5.0 and 14.2 fold, respectively). In the case of P2X4R, a significant decrease in relative mRNA levels was observed only in MKN-45 cells, with no significant changes in the other GC-derived cell lines (Figure 2A). To establish a possible relationship between P2Y2R and P2X4R, we performed a P2Y2R/P2X4R relative expression ratio, finding the following pattern: in all GC-derived cell lines this ratio was in the range of 3–6 fold in favor of P2Y2R, being these ratios obtained mainly by the P2Y2R overexpression on AGS and MKN-74 cells, and, interestingly, by a combination of the modest P2Y2R overexpression and P2X4R downregulation on MKN-45. However, in all of these cases, the final change in gene expression, independently if they were a result of P2Y2R increase or P2X4R decrease, always favors the predominance of P2Y2R over P2X4R (Figure 2B).

### 3.3. Changes in P2Y2R and P2X4R Expression Modify Cell Viability in Non-Tumoral and GC Cell Lines

To further establish the role of P2Y2R and P2X4R in cell proliferation, we performed molecular interventions like overexpression (OE) or knockdown (KD) of these receptors via transient transfections. After testing these protocols on AGS cells, we were capable to reduce by 70–80% (KD) or increase by 6–10 fold (OE) the expression of P2Y2R and P2X4R (Supplementary Figure S1).

**Figure 3:**
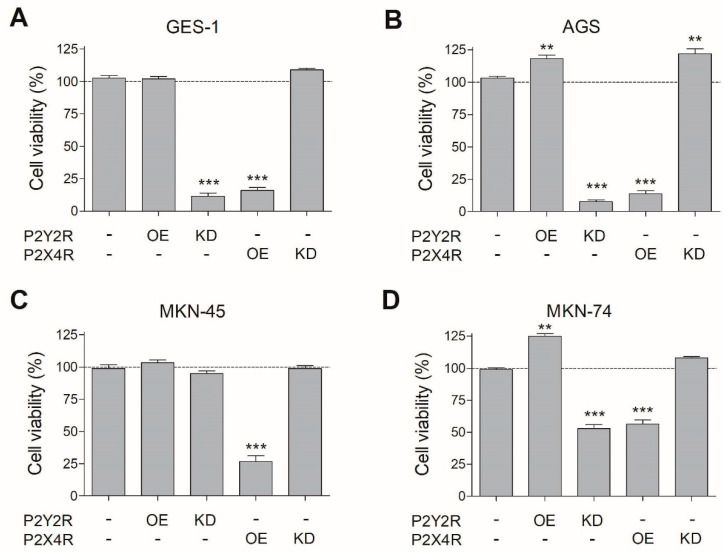
Effects of molecular interventions on P2Y2R and P2X4R on cell proliferation. (**A**–**D**) Overexpression (OE) or silencing (KD) of P2Y2R or P2X4R were performed using a 6 h transfection protocol. After 48 h post-transfection, resorufin fluorescent signal was measured on GES-1 (**A**), AGS (**B**), MKN-45 (**C**), and MKN-74 (**D**) cell lines. Cell proliferation values correspond to the RFU ratio between each molecular intervention (KD and OE) and its non-transfected WT condition. ** *p* < 0.01; *** *p* < 0.001 Student’s *t*-test comparing each molecular intervention with its non-transfected condition for each one of the analyzed cell lines (*n* = 8).

Following these interventions, cell viability assays were performed after 48 h of cell culture. We found a significant decrease in cell viability in all the cells tested with P2Y2R KD, except for MKN-45 cells in which this intervention had no effect (Figure 3). Regarding P2X4R, overexpression of this receptor reduced cell viability in all three GC-derived cell lines (Figure 3). On the other hand, P2Y2R OE increased cell viability only on AGS and MKN-74 cells (Figure 3B,D). These results with molecular interventions indirectly suggest that extracellular nucleotides are being released by cultured cells and having effects on cell proliferation after interacting with P2Y2R or P2X4R. This situation was confirmed by treating AGS cells with 5 U/mL apyrase for 48 h, which reduced cell proliferation by 20%, as compared to untreated cells (Supplementary Figure S2A). These results confirm relevant roles for P2Y2R and P2X4R in cell viability.

### 3.4. Changes in P2Y2R and P2X4R Expression Modify TEER in Non-Tumoral and GC Cell Lines

**Figure 4:**
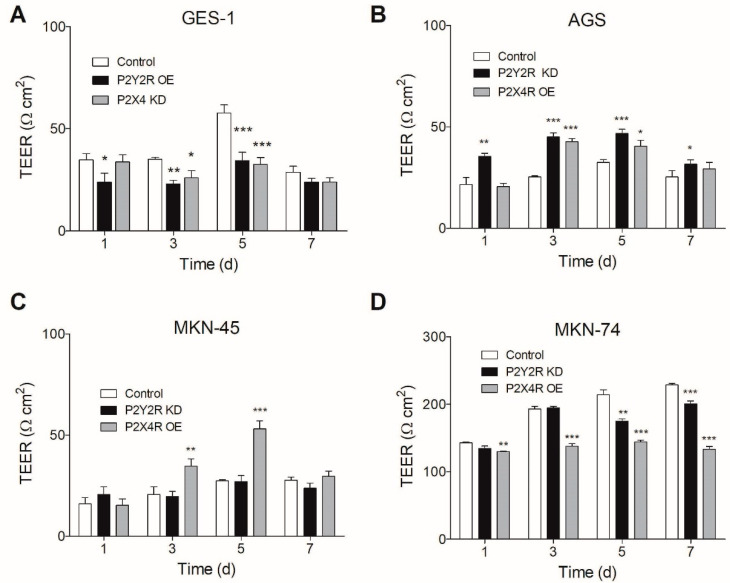
Effects of molecular interventions of P2Y2R and P2X4R on TEER. (**A**–**D**) TEER measurements were made on GES-1 (**A**), AGS (**B**), MKN-45 (**C**), and MKN-74 (**D**) cell lines after overexpression (OE) or silencing (KD) of P2Y2R or P2X4R. * *p* < 0.05; ** *p* < 0.01; *** *p* < 0.001 Student’s *t*-test performed by comparing each molecular intervention with its respective non-transfected condition for each measurement day and studied cell line (*n* = 4).

To establish the basal TEER levels on each cell line, we measured this electrical resistance on non-transfected cells, obtaining TEER values that ranged from 15 to 140 Ω cm^2^. As a non-tumoral cell line, GES-1 cells showed TEER values ranging from 26 to 62 Ω cm^2^ at days 1 to 7 of cell culture (Figure 4A). When we analyzed the non-transfected GC-derived cell lines AGS and MKN-45, we found TEER values consistently smaller than those observed for GES-1, but, in contrast, MKN-74 cells exhibited significantly higher TEER values. In all cases, TEER values increased after 5 days of culture, and this is probably related to cells reaching confluence after this period (Figure 4B–D). When we changed the expression of purinergic receptors on non-tumoral GES-1 cells trying to emulate a “tumoral phenotype”, overexpressing P2Y2R or silencing P2X4R, both interventions significantly reduced TEER values at days 3 and 7 (Figure 4A). In GC-derived cell lines, we decided on a different approach: to change the expression profile of purinergic receptors to emulate a “non-tumoral phenotype”. To do this, we performed P2Y2R KD and P2X4R OE as molecular interventions. In the case of AGS cells, P2Y2R KD and P2X4R OE significantly increased TEER values at days 3 and 5 of culture (Figure 4B). Interestingly, in MKN-45 cells P2X4R OE but not P2Y2R KD increased TEER values at days 3 and 5 of cell culture (Figure 4C). A different situation was observed for MKN-74 cells, which exhibited the highest TEER values in control conditions; in these cells, P2X4R OE did not increase but decreased TEER values at days 3–7 of cell culture, and P2Y2R KD slightly decreased TEER values at days 5 and 7 of culture (Figure 4D). Compiled together, these results show different TEER patterns for every cell line in non-transfected conditions, which can be modified from normal-to-tumoral phenotype and vice-versa after intervening in the expression of P2Y2 and P2X4 purinergic receptors.

### 3.5. Purinergic Stimulation Changes the Expression of Epithelial and Mesenchymal Markers in Non-Tumoral and GC-Derived Cell Lines

**Figure 5:**
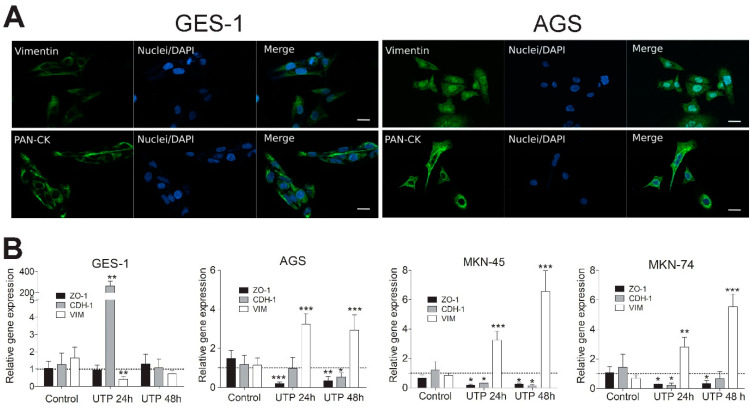
Effect of purinergic stimulation on the expression of epithelial and mesenchymal markers. (**A**) Basal expression of vimentin and Pan-CK in GES-1 (**left**) and AGS (**right**) by immunofluorescence assay and confocal microscopy detection. A representative image was acquired avoiding signal saturation. Both proteins are present on GES-1 and AGS cell lines. Scale bar: 20 µm. (**B**) mRNA levels were obtained by qPCR after treating GES-1, AGS, MKN-45, and MKN-74 cells with 100 μM UTP for 24 h. Gene expression calculations were performed using the 2-ΔΔC_q_ method, hB2M as referential gene, and UTP-untreated control cells as basal expression condition. * *p* < 0.05; ** *p* < 0.01; *** *p* < 0.001 Student’s *t*-test performing comparisons between control and UTP-treated condition for each gene and cell line (*n* = 4).

After we confirmed the opposite roles of P2Y2R and P2X4R on GC cell viability and TEER, we focused our work on studying deeper the signaling pathways and effectors elicited after P2Y2R activation. In the first series of experiments, we tested the basal subcellular distribution of pan-cytokeratin and vimentin, which are epithelial and mesenchymal markers, respectively, by immunofluorescence on GES-1 and AGS (Figure 5A). Pan-cytokeratin (Pan-CK) immunoreactivity was perinuclear in both cells types, while vimentin is perinuclear in GES-1 and AGS, but also presented a significant colocalization with nuclei (28.34 ± 2.17%, *n* = 4–7) in AGS cells (Figure 5A and Supplementary Figure S3). These observations suggest that GES-1 cells have an epithelial phenotype while AGS cells are partially mesenchymal. Next, we measured the mRNA levels of vimentin (VIM) and the epithelial markers E-cadherin (CDH-1) and tight junction protein-1 (ZO-1) via qPCR after treating GES-1 and GC-derived cell lines with 100 μM UTP, a selective P2Y2R agonist, for 24 and 48 h using untreated cells as basal expression control (Figure 5B). In GES-1 cells we found an important CDH-1 overexpression and VIM downregulation at 24 h of UTP simulation, without any changes on ZO-1 expression; however, these changes returned to control values after 48 h of UTP stimulation (Figure 5B, left graph). In contrast, on GC-derived cell lines, UTP stimulation significantly increased VIM and decreased ZO-1 relative gene expression at both 24 and 48 h of stimulation (Figure 5B). In the case of CDH-1, we found a significant decrease of this epithelial marker after UTP-stimulation at 24 and 48 h of stimulation on MKN-45 cells, after 48 h on AGS cells, and after 24 h on MKN-74 cells (Figure 5B). As an additional control, we performed experiments using 5 U/mL apyrase to deplete extracellular ATP of our cultures, finding that apyrase-treated AGS cells had no significant changes on VIM, CDH-1 nor ZO-1 mRNA levels compared to untreated AGS cells (Supplementary Figure S2B) Altogether, these results suggest a role for P2Y2R activation in promoting EMT on GC cell lines and stimulating epithelial phenotype on non-tumoral GES-1 cells.

### 3.6. Microarray Analysis of AGS Cells after UTP Stimulation

**Figure 6:**
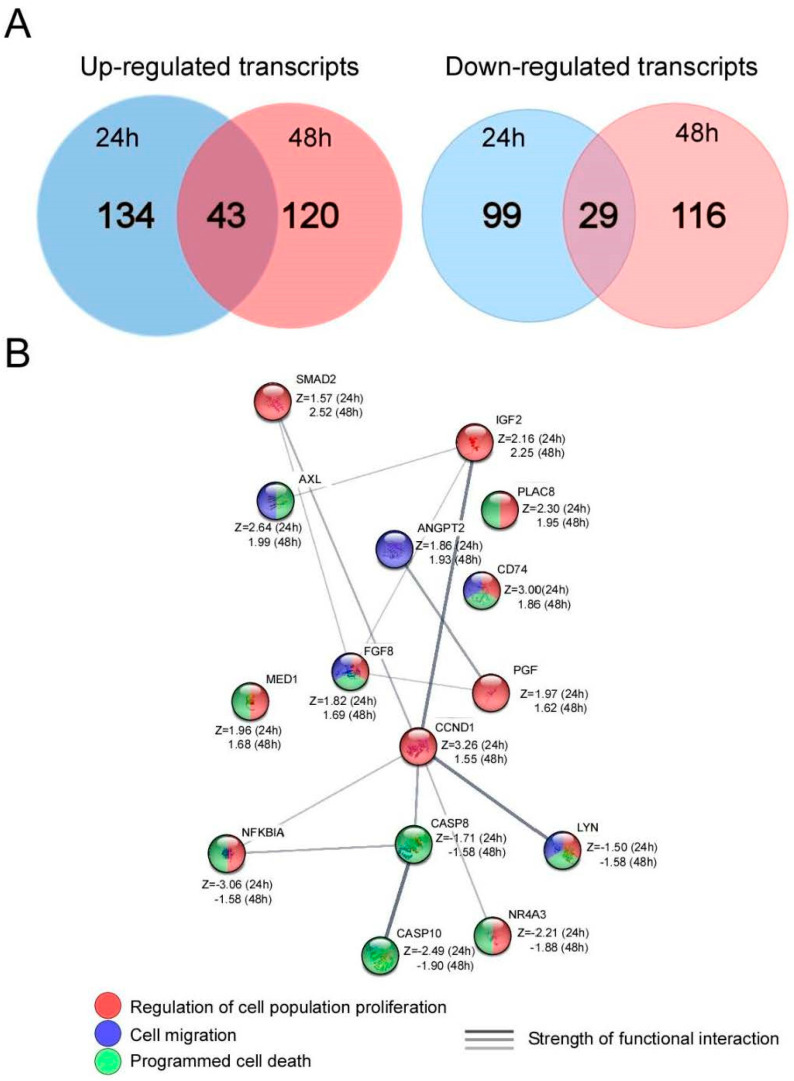
Microarray analysis in AGS cells at 24 and 48 h of UTP stimulation. An experiment of cDNA microarrays was made in AGS cells stimulated by 24 or 48 h with 100 mM UTP hybridized with a library of 1902 transcripts related to human cancer. (**A**) Venn diagram showing the number of up and down-regulated transcripts at 24 or 48 h of UTP stimulation and the SHARED transcripts subgroup. (**B**) After the analysis of SHARED transcripts by GO, noteworthy transcripts were grouped and analyzed in the STRING platform to find functional networks. Z (z-score). Information about the transcripts proteins and functions can be found in Supplementary Table S3.

To further identify the signaling pathways induced by UTP-induced P2Y2R activation on GC-derived cell lines, a microarray-based experiment was performed. AGS cells were stimulated with UTP 100 mM for 24 or 48 h, total RNA was isolated, cDNA synthesized, and hybridized with a library of 1920 transcripts related to human cancer. Data analysis was focused on GO as described in the Materials and Methods section. UTP induced notable changes, compared to non-stimulated cells. There were 134 and 120 up-regulated and 99 and 116 down-regulated transcripts, after 24 and 48 h of UTP treatment, respectively, that showed significant changes (Z-score +/− 1.5). Additionally, both conditions shared 43 up-regulated and 29 down-regulated transcripts (Figure 6A). Data analysis with GO revealed that the main up-regulated categories at 24 and 48 h were identical: (1) *Cell differentiation* (GO:0030154), (2) *Regulation of cell population proliferation* (GO:0042127), and (3) *Cell migration* (GO:0016477). Conversely, the most down-regulated categories after 24 and 48 h treatment were: (1) *Response to drug* (GO:0042493), (2) *Programmed cell death* (GO:0012501), and (3) *Positive regulation of cell adhesion* (GO:0045785). The STRING analysis values obtained (description, count in network, and FDR) are summarized in Table 1, and the specific up and downregulated transcripts are detailed in Supplementary Table S2.

To perform sharper data analysis and to identify those transcripts associated with cellular processes that were already activated at 24 h and sustained after 48 h, the shared transcripts group was analyzed by GO, describing the categories that were up or down-regulated and their values obtained in STRING platform (description, count in network and FDR); these are shown in Table 2. The terms identified by GO-analysis of the up-regulated transcripts were *Cell differentiation* and *Regulation of cell population proliferation*, like previous observations at 24 and 48 h of UTP treatment, but also *Negative regulation of cell adhesion* (GO:007162) was identified. Regarding the down-regulated transcripts, the categories identified by GO-analysis were identical to those recognized for 24 and 48 h of UTP stimulation (Table 1 and Table 2). In our results, several growth factors such as placental growth factor (PGF), fibroblast growth factor 8 (FGF8), and insulin-like growth factor 2 (IGF2) as well as effectors such as SMAD2 and cyclin D1 (CCND1) increased their expression level (Supplementary Table S3). Moreover, some transcripts, like CD74, PPARBP, and PLAC8, were identified in agreement with the upregulation of *Negative regulation of apoptosis,* and CASP10, CASP8, and NFKBIA transcripts were identified to explain the down-regulation *of Programmed cell death* genes, highlighting the effects of UTP on the extrinsic pathway of apoptosis (Supplementary Table S3). Furthermore, analyzing the up-regulation of the group of genes related to *Cell Differentiation,* AXL stands as a relevant transcript (Z-score = 2.63 and 1.99, at 24 and 48 h respectively). This transcript codifies for the AXL tyrosine kinase receptor (AXL), a member of the TAM family, whose ligand is called Gas6, and both belong to EMT-related pathways, being related with metastatic phenotype induction [29]. Moreover, an up-regulation of SMAD2 transcript was observed (Z score = 1.57 and 2.51, at 24 and 48 h respectively) (Supplementary Table S2), which codes for the transcription factor Smad-2, the main effector of the transforming growth factor- β (TGF-β) signaling pathway (Supplementary Table S3). Additionally, we detected the upregulation of transcripts belonging to the *Negative regulation of cell adhesion* group and, concomitantly, the downregulation of *Positive regulation of cell adhesion*; indicating a general inhibition of the cell adhesion process, and sustaining the idea of epithelial phenotype loss, as suggested in other experiments of this article. In the group of transcripts relevant for cell adhesion and EMT, the upregulation ANGPT2 transcript (Z score = 1.85 and 1.93 at 24 and 48 h, respectively), is highlighted. This transcript codifies for angiopoietin 2 (Ang-2), a potent inductor of EMT in lung cancer cells [30]. To find possible functional interactions between the shared group transcripts, we analyzed them with STRING, and interestingly a functional network was observed. This analysis predicted a relationship between both up and down-regulated transcripts, obtaining an important upregulation of CCND1 transcript, which codifies for cyclin D1, that belongs to the *Regulation of cell population proliferation* group, and the downregulation of NR4A3, a transcription factor that has been linked to gastric cancer prognosis [31] and is part of the *Positive regulation of cell adhesion* group (Figure 6B). Finally, and based on evidence that extracellular ATP present in the tumor microenvironment is an important regulator [32], we investigated the effects of extracellular ATP depletion with apyrase (5 U/mL for 24 h) on the transcriptional activity of AGS cells using cDNA microarrays. The main categories detected by GO analysis for up-regulated transcripts were: *Negative regulation of mitotic cell cycle* (GO:0045930, FDR = 0.00031) and *Positive regulation of cell adhesion* (GO:0045785, FDR = 0.0076); for down-regulated transcripts, the categories were *Positive regulation of cell migration* (GO:0030335, FDR 0.0022) and *Positive regulation of MAPK cascades* (GO:0043410, FDR 0.00044) (Supplementary Table S4). This analysis reveals that the group of transcripts regulated by extracellular ATP depletion is mainly related to inhibition of both cell migration and proliferation.

## 4. Discussion

According to Globocan 2020, gastric cancer (GC) ranks fourth in the number of cancer-related deaths per year, with 768,793 casualties in 2020 (Global Cancer Observatory; https://gco.iarc.fr/; last accessed 1 July 2021). Additionally, differential diagnosis of GC supposes a substantial challenge due to the vague nature of its symptomatology (anemia, dysphagia, abdominal pain, anemia, and anorexia) on early and later phases, which can be easily attributed to other milder gastrointestinal diseases, supposing an important delay on cancer therapy and prognosis [33,34]. This situation of unspecific symptoms, high mortality, and difficult diagnosis hinders the success rate of chemotherapeutics but also leads the scientific effort towards the development of novel tools for early diagnosis, or therapeutic strategies with higher efficacy and selectivity against cancer cells than current antineoplastic approaches [35,36]. Inspired by this effort, this work was focused on describing the role of P2Y2 and P2X4 purinergic receptors on EMT and its link with the metastatic potential of GC-derived cell lines.

Our measurements of extracellular ATP concentrations by non-tumoral and GC-derived cell lines corroborated the known fact that tumoral cells synthesize and release higher amounts of ATP to the extracellular media [37]. In our experiments, GC-derived cell lines had substantially higher (6–14 fold) extracellular ATP concentrations, strengthening the idea that paracrine/autocrine released nucleotides by tumoral cells can stimulate purinergic signaling and tumor growth, as we have previously demonstrated for GC-derived cells [14]. The effects of extracellular nucleotides on cell proliferation were further confirmed by treating AGS cells with 5 U/mL apyrase, which reduced cell viability as compared to untreated AGS cells (Supplementary Figure S2A). This phenomenon has also been observed on human lung cancer cell lines H292 and PC-9, where ATP release was associated with P2X7R activation and cell migration induction [38]. High concentrations of extracellular ATP have been also reported on breast cancer, where this situation was related to an increase in cancer invasion and metastatic potential, and overexpression of lysyl oxidase-like 2 (LOXL2) and matrix metalloproteinase-9 (MMP-9), which are direct targets of hypoxia-inducible factor 2α (HIF-2α), a relevant EMT agent [39]. This information sustains our findings and could explain why moderately, and highly invasive metastatic cell lines (MKN-74 and MKN-45, respectively) have higher extracellular ATP concentrations than human primary gastric adenocarcinoma cells (AGS).

Our findings of P2Y2R and P2X4R relative gene expression on AGS, MKN-45, and MKN-74 cells against the non-tumoral GES-1 cell control reveal a 2.3-to-14.0-fold overexpression of P2Y2R and a reduction of the P2X4R expression only observed on MKN-45 cells. When we performed a ratio between the relative expression of these two receptors, a 3:1 to 6:1 P2Y2R/P2X4R ratio is observed in these GC-derived cell lines, with certain differences that could be attributed to the stage and grade of gastric cancer that these cell lines represent: AGS correspond to human primary adenocarcinoma cells, and MKN-74 and MKN-45 are human metastatic adenocarcinoma cells with a moderate and poor differentiation rate, respectively [40]. Considering these differences, our results of P2Y2R and P2X4R relative expression could provide an idea about the progression of gastric cancer. This relationship between P2Y2R and P2X4R gene expression remarks the potential of purinergic signaling as a prospective biomarker for gastric cancer progression, as similar studies have reported for P2X7R, whose overexpression is related to tumor size, metastatic potential, and overall survival in human colorectal and gastric cancer [41,42]. The opposite effects observed after P2Y2R or P2X4R activation can be explained by the different signaling pathways that these receptors can trigger: P2Y2R is a Gαq-coupled receptor that increases intracellular calcium and activates protein kinase C but also can activate other signaling pathways such as PI3K-Akt [43] or ERK1/2 phosphorylation via a protein kinase C (PKC)-dependent mechanism [44,45]. On the other hand, P2X4R is an ATP-gated ion channel that rapidly depolarizes the plasma membrane by the influx of sodium and calcium [9]. In this context, the P2Y2R/P2X4R expression ratio could function as a predictor of which of these signaling pathways will predominate, resulting in a final proliferative or antiproliferative effect of ATP and related nucleotides. Additionally, the differences in ATP affinity between P2Y2R and P2X4R could explain that one subtype has a predominant activation in our experiments. P2Y2Rs have an ATP EC_50_ ≤ 1 µM, whereas P2X4R has an EC_50_ of around 10 µM [46]. As our measurements reveal, extracellular ATP concentrations were in the range of 1–2 µM, favoring the activation of P2Y2R, and therefore a positive effect on cell proliferation.

When we modified the P2Y2R and P2X4R expression using molecular interventions and measured their effects on cell proliferation, our results describe that, at different rates, P2Y2R overexpression induces cell proliferation, while P2X4R overexpression has an antiproliferative effect on GC-derived cell lines. Conversely, when knockdown strategies were applied for both receptors, an inverse trend of cell proliferation inhibition and stimulation for P2Y2 KD and P2X4 KD, respectively, appeared for the studied cell lines. These effects on cell proliferation are in accordance with those observed in pharmacological assays performed on P2Y2R in gastric cancer [14], pancreatic ductal adenocarcinoma [47], esophageal [48], breast carcinoma [49], and on P2X4R in gastric [14], breast [50], and prostate cancer [51]. Interestingly, in MKN-45 cells P2Y2R KD did not have any effect on cell proliferation; however, this result was not unexpected, since previous reports using 1–300 µM applications with the P2Y2R agonist UTP in this cell line had minor effects on cell proliferation [14]; conversely, P2X4R OE was a successful strategy for reducing cell proliferation on this cell line. These results suggest that purinergic approaches for GC therapy could be applied for both early or late-stage tumors, but their outcomes and specific design will depend on the capacity of these strategies to restore the purinergic balance that these tumors have lost depending on their grade or stage.

Although there are fewer studies on gastric than other types of cancer, such as lung, breast, or ovarian cancers [19], some interesting results about the role of purinergic signaling have arisen from different research groups. It has been shown that the activation of P2Y6R by its preferring agonist UDP decreases GC proliferation via inhibition of the β-catenin signaling pathway, which is dependent on store-operated calcium entry (SOCE) [52]. Interestingly, it has been reported in this work that the expression of P2Y6R is diminished in GC-derived cells and gastric tumors [52]. Another receptor that has been linked with GC is P2X7R, whose expression is increased in gastric tumors and associated with lower survival of GC patients [42]. Adenosine and ATP inhibit cell proliferation and induced apoptosis in lung cancer [53,54]. Other studies have found that adenosine promotes metastasis, cell invasion, and expression of EMT-related genes through the activation of the A_2A_ receptor, a signaling pathway that involves the activation of PI3K, Akt, and mTOR [55]. Our group has also studied the role of purinergic signaling in GC, finding a positive and negative effect on cell proliferation for P2Y2R and P2X4R, respectively [14]. 

TEER experiments and molecular interventions revealed that, at non-transfected conditions, GC-derived cell lines had lower electrical resistance values than their non-tumoral counterpart GES-1 (TEER ranging from 26 to 62 Ω cm^2^), with the only exception of MKN-74 cells, which have noticeably higher TEER levels (from 140 to 230 Ω cm^2^). This situation observed for AGS and MKN-45 cells are consistent with the high cell motility and EMT-induction profile defined by the aberrant nuclear vimentin distribution that has been described for both cell lines during a high-throughput analysis of gastric tumors for drug screening [56]. MKN-74 TEER levels can be explained considering their moderate differentiation grade, which gives them an epithelial-like phenotype, and their high P2Y2R-induced proliferation rates [14], allowing them to reach 100% confluence after 24 h post-seeding, giving them the electrical resistance pattern observed by us. When we performed KD and OE experiments during TEER measurements, the effect of P2Y2R KD was an increase in TEER values on AGS towards "non-tumoral" values like the ones observed for GES-1 cells, being P2X4R OE capable to do this only at days 3 and 5. P2Y2R KD on MKN-74 reduced TEER values, but to a minor extent compared to P2X4R OE, which consistently reduced TEER at days 1, 3, 5, and 7. On MKN-45, a late-stage high-grade model for gastric cancer, TEER values were increased only after 3 days post-P2X4R OE, showing no effect for P2Y2R KD. These results show that the countereffects observed for P2Y2R and P2X4R in cell proliferation are also present in cell migration experiments. This last point can also be proven by the interventions performed on GES-1 cells, where the opposite strategies, P2Y2R OE and P2X4R KD, decreased TEER, mimicking a GC situation. Restoration of non-tumoral TEER values by the use of prospective drugs targeting EMT regulators like matrix metalloproteinases or tight junction proteins could be used as promising alternatives for reducing cell motility, invasion, and metastasis on gastric cancer [24].

To evaluate the involvement of P2Y2R activation on EMT, we performed qPCR experiments to measure changes in epithelial and mesenchymal markers after P2Y2R activation with its specific agonist, UTP. In general, we found overexpression of vimentin and a decrease of ZO-1 and CDH-1 mRNA levels on GC-derived cell lines treated for 24 and 48 hrs with UTP. Changes towards a mesenchymal phenotype were not uniform on GC-derived cell lines, being more noticeable on MKN-45, which is in accordance with its features as a poorly differentiated adenocarcinoma cell line with a mutation on the CDH-1 promoter region [57]. In line with this, the decrease in CDH-1 mRNA levels in AGS cells is significant only after 48 h of UTP stimulation, and its 3-fold vimentin overexpression remains equal at 24 and 48 h and can be related to the phenotype of these cells ab initio. On GES-1 cells, an important CDH-1 overexpression is seen at 24 h, and this phenomenon can be explained by induction of cell proliferation via UTP. Considering that this cell line corresponds to gastric epithelia, stimulation of cell proliferation correlates with overexpression of E-cadherin (CDH-1). When GES-1 cells reached confluence at 48 h, CDH-1 mRNA levels were normalized. To understand more about the localization patterns of epithelial and mesenchymal markers on gastric cancer, we performed a series of immunocytochemistry experiments on GES-1 and AGS cells, using proper antibodies to detect pan-cytokeratin as epithelial protein, and vimentin as mesenchymal protein. The expression of the later protein exhibited a signal on GES-1, being vimentin mainly located at the cytoskeletal level on these cells; however, on the GC-derived AGS cells vimentin showed higher expression and an aberrant dotted expression pattern both at cytoplasmic and nuclear levels. This atypical expression of nuclear vimentin has been reported as a cell survival mechanism via ATM kinase that phosphorylates vimentin in response to DNA damage on colorectal and lung cancer cells HCT-116 and A549, respectively, triggering EMT to increase cell motility and metastatic potential on early stages of treatment with the traditional chemotherapeutic drug camptothecin [58]. Nuclear vimentin has also been proposed as a metastasis and prognosis biomarker on paraffin-embedded nasopharyngeal carcinoma biopsies [59], highlighting the metastatic potential and aggressiveness of gastric cancer, even on AGS cells, that could represent early-stage/low-grade tumors. These changes towards the overexpression of mesenchymal markers and downregulation of epithelial markers on GC-derived cell lines could also be explained by the higher ATP concentrations found at the extracellular level, which have been previously reported to induce the expression of EMT-related proteins like vimentin, Snail and Slug on the human non-small cell lung cancer cell line A549 [60]. To relate these EMT-induction effects to extracellular ATP levels on gastric cancer cell lines, we treated AGS cells with 5 U/mL apyrase for 48 h, and measured ZO-1, CDH-1, and VIM mRNA levels via qPCR, finding that apyrase-treated cells did not show any differences with untreated cells, suggesting that basal extracellular ATP plays a role on cell proliferation, but not on EMT in these particular experimental conditions. To trigger EMT, in our experiments, we had to specifically activate the P2Y2R with exogenous UTP (Figure 5 and Figure 6, and Supplementary Figure S2).

Regarding the microarray experiments, our approach allows us to appreciate a landscape of processes and pathways regulated by P2Y2R activation. First, we noted that UTP induces important changes in gene expression on AGS cells, indicating a role for P2Y2R in the regulation of cellular processes. The group of up and down-regulated differs between 24 or 48 h UTP-treatment groups, but also a group of shared transcripts was obtained. From these results, we infer that UTP-dependent P2Y2R signaling regulates different pathways at 24 and 48 h of stimulation, but at the same time, the regulation of some processes is sustained (Figure 6A). Clearly, UTP commands cell proliferation and apoptosis inhibition, indicating that UTP-responsive receptors regulate tumor growth. These observations are in agreement with previous work from our workgroup where we documented that UTP induces proliferation of gastric carcinoma cell lines [14]. The GO analysis for transcripts that modified their expression at 24 and 48 h, allowed us to detect categories related to important cell tumor process as cell proliferation, cell differentiation, and cell migration (Table 1 and Supplementary Table S2), suggesting a role for P2Y2R in tumor growth and invasiveness. The analysis of shared transcripts was aimed to identify these transcripts and cell processes whose up- or downregulation is sustained after 24 and 48 h of UTP stimulation. In general, the processes regulated by UTP/P2Y2R in the shared groups were similar to those detected after analyzing the 24 and 48 h conditions separately (Table 2). However, the group of transcripts involved in these responses was reduced, making it possible to observe their identity and characteristics. From this analysis, it was observed that three positive transcriptional regulation of genes coding for growth factors such as PGF, IGF2, and FGF8, is related to an induction of cell proliferation which converges in the up-regulation of CCND1, a transcript that codifies for cyclin D1. This protein is involved in the regulation of G1/S transition and its overexpression has been documented in the proliferation of gastric cancer cells [61]. IGF2 has also been associated with cell proliferation and differentiation in gastric cancer [62,63]. These changes are concomitant with the down-regulation of apoptotic elements such as CASP8 and CASP10. Furthermore, GO analysis revealed that UTP is also affecting cell migration and adhesion pathways, in agreement with the experimental evidence shown in this work. Another noteworthy up-regulated transcript up-regulated is ANGPT2, coding for angiopoietin 2 (Ang-2); Ang-2 has been identified as a metastasis inducer in melanoma [64] as well as a bad prognosis biomarker in progress and development of colorectal cancer in Japanese [65] and Chinese [66] populations. AXL and SMAD2 were also identified as up-regulated transcripts, coding the first for the AXL tyrosine kinase receptor (AXL); whose ligand is named Gas6. The AXL/Gas6 pathway is a potent EMT inducer and a metastatic phenotype promoter [29], that has been proposed as a pharmacological target in breast cancer for its role in controlling the invasive phenotype of cancer cells [67]. On the other hand, SMAD2 codifies for the transcription factor Smad-2, the canonical effector of TGF-b. The TGF-β/Smad-2/Snail pathway is a master regulation mechanism of EMT induction, and has a recognized relevance in cell differentiation, guiding the metastatic state in a broad number of cancer types [68]. To evaluate if the shared transcripts regulated by UTP/P2Y2 in the microarray are functionally related, we analyzed this group using STRING [27]. This analysis served to identify that the mentioned transcripts are related in broad informational networks, appearing CCND1 as an integrator node of this group of transcripts (Figure 6B). Altogether, this analysis of gene expression regulated by P2Y2R activation in gastric cancer cells opens new ways to understand the network of molecular mechanisms interacting with purinergic signaling elements that regulate important cellular processes in cancer, like cell proliferation stimulation, cell death inhibition, and EMT induction, that will allow the identification of new specific therapeutic targets.

Interestingly, microarrays reinforced the notion that extracellular ATP self-released by GC cells is responsible, at least in part, for cell proliferation and migration. The depletion of ATP by addition of apyrase at the culture medium by 24 h induced the following modifications in gene expression (Supplementary Table S4): (1) up-regulation of transcripts related to inhibition of cell proliferation (GO category: *negative regulation of mitotic cell cycle*). One example of this category is ATM, which codifies for ataxia-telangiectasia mutated protein, a serine-threonine kinase-related with cell cycle regulation and DNA damage response [69]; (2) down-regulation of transcripts related with proliferative signals (GO category: *positive regulation of MAPK cascades*); (3) Up-regulation of transcripts related with positive regulation of cell adhesion, and (4) Down-regulation of transcripts related *to positive regulation of cell migration*; an example on this category is SRC, coding for SRC proto-oncogene, non-receptor tyrosine kinase [70]. Therefore, ATP, extracellular nucleotides, and purinergic signaling, in general, play a role in the pathophysiology of gastric and other types of cancer.

In summary, our results show a potential relationship between P2Y2R and P2X4R expression and GC grade and stage, suggesting that high levels of P2Y2R are associated with primary or moderately differentiated gastric tumors, and a decrease in P2X4R expression is seen in poorly differentiated gastric adenocarcinomas. A balance between P2Y2R and P2X4R expression profiles seems to have a role in cell proliferation and migration potential, and the activation of P2Y2R results in several changes in gene expression related to cancer growth, progression, differentiation, and metastasis. Although these findings are preliminary, purinergic signaling constitutes a promising target that can be used both as a biomarker, like the P2Y2R/P2X4R ratio, and/or a therapeutic target for gastric cancer.

## Figures and Tables

**Table 1:** STRING analysis from up-regulated and down-regulated genes at 24 and 48 h of UTP-stimulation.

**Up-Regulated Genes in Networks**					
GO Term	Description	Count in Network		FDR	
		24 h	48 h	24 h	48 h
GO:0030154	Cell differentiation	45 of 3457	44 of 3457	0.00011	2.04 × 10^−5^
GO:0042127	Regulation of cell population proliferation	40 of 1594	33 of 1594	2.47 × 10^−10^	7.65 × 10^−8^
GO:0016477	Cell migration	15 of 812	19 of 812	0.0046	2.62 × 10^−5^
**Down-Regulated Genes in Networks**					
GO:0042493	Response to drug	20 of 900	15 of 900	1.85 × 10^−6^	0.0037
GO:0012501	Programmed cell death	14 of 1042	15 of 1042	0.0072	0.0108
GO:0045785	Positive regulation of cell adhesion	9 of 375	11 of 375	0.0018	0.00041

**Table 2:** STRING analysis from genes shared between 24 and 48 h of UTP stimulation.

**Up-Regulated Genes in SHARED Network**			
GO Term	Description	Count in Network	FDR
GO:0030154	Cell differentiation	15 of 3457	0.0415
GO:0042127	Regulation of cell population proliferation	14 of 1594	0.00078
GO:007162	Negative regulation of cell adhesion	4 of 245	0.0233
**Up-Regulated Genes in SHARED Network**			
GO:0042493	Response to drug	7 of 900	0.0069
GO:0012501	Programmed cell death	6 of 1042	0.0336
GO:0045785	Positive regulation of cell adhesion	4 of 375	0.0243

## Supplementary Materials

The following are available online at https://www.mdpi.com/article/10.3390/pharmaceutics13081234/s1, Figure S1: Optimization of P2Y2R and P2X4R overexpression and knockdown protocols on AGS cell lines; Figure S2: Effect of extracellular ATP hydrolysis on AGS cell proliferation and EMT-related genes expression; Figure S3: Pan-CK and Vimentin shows an altered subcellular distribution in AGS cells, compared to GES-1; Table S1: Primer sequences and characteristics for each analyzed gene; Table S2: Transcript changes after 24 h of UTP-treatment in AGS cells; Table S3: Transcript changes in SHARED network after 24 and 48 h of UTP-treatment in AGS cells; Table S4: Transcript changes after apyrase treatment in AGS cells.
