# Supplementary material for: Purinergic P2Y2 and P2X4 Receptors Are Involved in the Epithelial-Mesenchymal Transition and Metastatic Potential of Gastric Cancer Derived Cell Lines
Source: Pharmaceutics. 2021 Aug 11;13(8):1234. doi: 10.3390/pharmaceutics13081234 (PMC8398939; doi:10.3390/pharmaceutics13081234)
Supplement: Supplementary file 1 [file pharmaceutics-13-01234-s001.zip › pharmaceutics-1312793-supplementary.pdf]

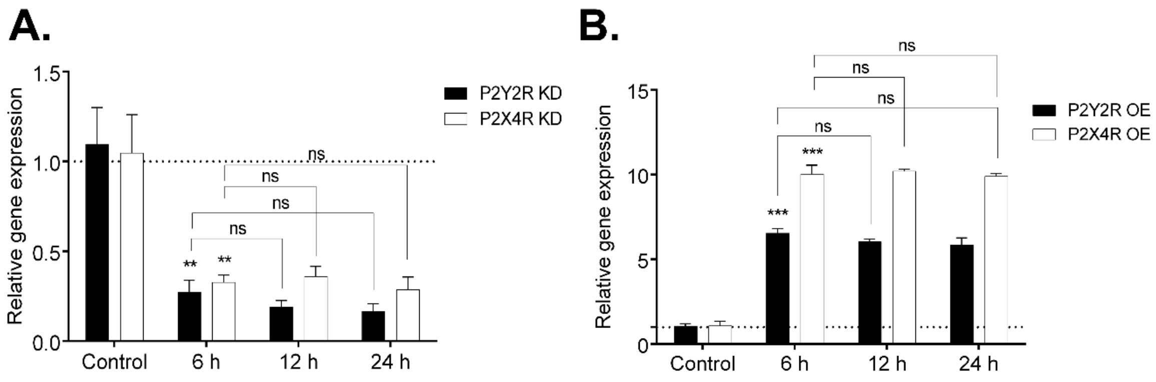

Supplementary Figure 1. Optimization of P2Y2R and P2X4R overexpression and knockdown protocols on AGS cell lines. A. Relative gene expression assessment via qPCR after P2Y2R and P2X4R knockdown via transfection for 6, 12 and 24 hours, and using AGS cells transfected with Silencer Select siRNA Negative Control (siRNA ID: 4390843) as control condition. **B.** mRNA levels obtained by qPCR after overexpressing P2Y2R and P2X4R via transfection for 6, 12 and 24 hours, and using AGS cells transfected with empty pEGFP-N1 and pIRES2-EGFP vectors as basal conditions for P2Y2R and P2X4R expression, respectively. ns $p \geq 0.05$; ** $p < 0.01$; *** $p < 0.001$ Student's t-test comparing 6h-transfeccion groups against control condition, and among transfection times (*n* = 3).

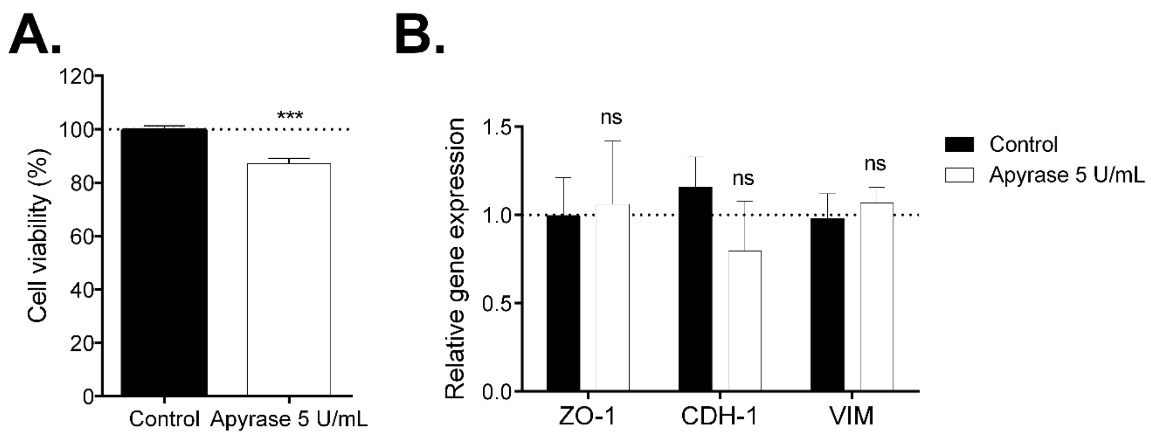

Supplementary Figure 2. Effect of extracellular ATP hydrolysis on AGS cell proliferation and EMT-related genes expression. A. Resazurin reduction measurements obtained after treating AGS cells with 5 U/mL apyrase for 48 h. Cell viability values correspond to the RFU ratio between the apyrase-treated condition and its untreated control condition. **B.** Relative gene expression assessment via qPCR after a 48 h treatment with 5 U/mL apyrase, using untreated AGS cells as basal condition to calculate ZO-1, CDH-1 and VIM expression. ns $p \geq 0.05$; *** $p < 0.001$ Student's t-test comparing apyrase-treated groups with their proper untreated control conditions (*n* = 3-5).

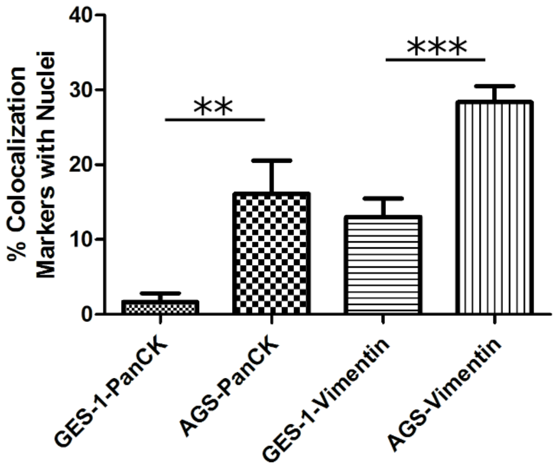

Supplementary Figure 3. Pan-CK and Vimentin shows an altered subcellular distribution in AGS cells, compared to GES-1. GES-1 and AGS cells lines were immunolabeled against Pan-CK, Vimentin and nuclei, and the co-localization between them was analysed by Mander´s coefficient (See immunofluorescence in material and methods). In AGS, both Pan-CK and Vimentin presented a significant increase of colocalization with nuclei compared with GES1 cell line. ** $p < 0.05$; *** $p < 0.001$ Student's t-test. (n = 4-7).

Supplementary Table 1

Supplementary Table 1. Primer sequences and characteristics for each analyzed gene				
**Gene**	**NCBI Reference Sequence**	**Primer Sequence**	**Amplicon size (bp)**	**Amplicon T_m (°C)**
P2Y2R	NM_176072.3	Fw: 5'-CAGGTCCAGGCGTGTGCATT-3'<br>Rv: 5'-AGCCACCTGACCAGGGCTTT-3'	150	84.0 ± 0.5
P2X4R	NM_001256796.2	Fw: 5'-GCCGTGCAACTGCTCATCCT-3'<br>Rv: 5'-ACACCCACCTTCTAGGGGACT-3'	91	81.0 ± 0.5
ZO-1	NM_001301025.3	Fw: 5'-GCCACCTGAAGATATTGTTCG-3'<br>Rv: 5'-CGCCAAATGATCTATCCACAC-3'	242	80.5 ± 0.5
CDH-1	NM_004360.5	Fw: 5'-AGAGGACCAGGACTTTGAC-3'<br>Rv: 5'-CACGAGCAGAGAATCATAAGG-3'	211	84.0 ± 0.5
VIM	NM_003380.5	Fw: 5'-CCCTTGACATTGAGATTGCC-3'<br>Rv: 5'-CGTGATGCTGAGAAGTTTCG-3'	213	80.0 ± 0.5
B2M	NM_004048.3	Fw: 5'-AAGTGGGATCGAGACATGTAAGCA-3'<br>Rv: 5'-GGAATTCATCCAATCCAAATGCGGC-3'	70	77.0 ± 0.5

Supplementary Table 2. Transcript changes after 24 ah of UTP-treatment in AGS cells.

Supplementary Table 2. Up-Regulated Transcripts after 24 h of UTP		
**GO:0030154 Cell differentiation**		
**Symbol**	**Description**	**Z-score**
TNFRSF11A	Tumor necrosis factor receptor superfamily, member 11a, NFKB activator; Receptor for TNFSF11/RANKL/TRANCE/OPGL; essential for RANKL-mediated osteoclastogenesis. Involved in the regulation of interactions between T-cells and dendritic cells; CD molecules	3.68
CCND1	B-cell lymphoma 1 protein; Regulatory component of the cyclin D1-CDK4 (DC) complex that phosphorylates and inhibits members of the retinoblastoma (RB) protein family including RB1 and regulates the cell-cycle during G(1)/S transition. Phosphorylation of RB1 allows dissociation of the transcription factor E2F from the RB/E2F complex and the subsequent transcription of E2F target genes which are responsible for the progression through the G(1) phase. Hypophosphorylates RB1 in early G(1) phase.	3.26
CD74	CD74 molecule, major histocompatibility complex, class II invariant chain; Plays a critical role in MHC class II antigen processing by stabilizing peptide-free class II alpha/beta heterodimers in a complex soon after their synthesis and directing transport of the complex from the endoplasmic reticulum to the endosomal/lysosomal system where the antigen processing and binding of antigenic peptides to MHC class II takes place. Serves as cell surface receptor for the cytokine MIF; CD molecules	3.00
EIF4G1	Eukaryotic translation initiation factor 4 gamma, 1; Component of the protein complex eIF4F, which is involved in the recognition of the mRNA cap, ATP-dependent unwinding of 5'-terminal secondary structure and recruitment of mRNA to the ribosome; Parkinson disease associated genes	2.99
VEGFA	Vascular endothelial growth factor A; Growth factor active in angiogenesis, vasculogenesis and endothelial cell growth. Induces endothelial cell proliferation, promotes cell migration, inhibits apoptosis, and induces permeabilization of blood vessels. Binds to the FLT1/VEGFR1 and KDR/VEGFR2 receptors, heparan sulfate and heparin.	2.96
GAS7	Growth arrest-specific protein 7; May play a role in promoting maturation and morphological differentiation of cerebellar neurons; F-BAR domain containing	2.82
CYP1A1	Cytochrome P450, family 1, subfamily A, polypeptide 1; Cytochromes P450 are a group of heme-thiolate monooxygenases. In liver microsomes, this enzyme is involved in an NADPH-dependent electron transport pathway. It oxidizes a variety of structurally unrelated compounds, including steroids, fatty acids, and xeotics.	2.78
AXL	Tyrosine-protein kinase receptor UFO; Receptor tkinase that transduces signals from the extracellular matrix intcytoplasm by binding growth	2.64

	factor GAS6 and which is thus regulating many phgical processes including cell survival, cell proliferation, migration, and differentiation. Ligand binding at the cell surface induces dimerization and autophosphorylation of AXL. Following activation by ligand, ALX binds and induces tyrosine phosphorylation of PI3- kinase subunits PIK3R1, PIK3R2 and PIK3R3; but also, GRB2, PLCG1, LCK and PTPN11.	
PDCD6	Apoptosis-linked gene 2 protein homolog; Calcium sensor that plays a key role in processes such as endoplasmic reticulum (ER)-Golgi vesicular transport, endosomal biogenesis, or membrane repair. Acts as an adapter that bridges unrelated proteins or stabilizes weak protein-protein complexes in response to calcium: calcium-binding triggers exposure of apolar surface, promoting interaction with different sets of proteins thanks to 3 different hydrophobic pockets, leading to translocation to membranes.	2.48
PLAC8	Placenta-specific gene 8 protein; Placenta specific 8	2.30
EZR	Cytovillin; Probably involved in connections of major cytoskeletal structures to the plasma membrane. In epithelial cells, required for the formation of microvilli and membrane ruffles on the apical pole. Along with PLEKHG6, required for normal macropinocytosis; A-kinase anchoring proteins	2.29
JUND	Transcription factor jun-D; Transcription factor binding AP-1 sites; Belongs to the bZIP family. Jun subfamily.	2.18
IGF2	Insulin-like growth factor 2 (somatomedin A); The insulin-like growth factors possess growth-promoting activity. Major fetal growth hormone in mammals. Plays a key role in regulating fetoplacental development. IGF-II is influenced by placental lactogen. Also involved in tissue differentiation.	2.16
ANXA2	Placental anticoagulant protein IV; Calcium-regulated membrane-binding protein whose affinity for calcium is greatly enhanced by anionic phospholipids. It binds two calcium ions with high affinity. May be involved in heat-stress response. Inhibits PCSK9-enhanced LDLR degradation, probably reduces PCSK9 protein levels via a translational mechanism but also competes with LDLR for binding with PCSK9; Belongs to the annexin family.	2.13
PARP1	ADP-ribosyltransferase diphtheria toxin-like 1; Involved in the base excision repair (BER) pathway, by catalyzing the poly(ADP-ribosyl)ation of a limited number of acceptor proteins involved in chromatin architecture and in DNA metabolism. This modification follows DNA damages and appears as an obligatory step in a detection/signaling pathway leading to the reparation of DNA strand breaks. Mediates the poly(ADP-ribosyl)ation of APLF and CHFR. Positively regulates the transcription of MTUS1 and negatively regulates the transcription of MTUS2/TIP150.	2.11
CAMK2A	Calcium/calmodulin-dependent protein kinase type II subunit alpha; CaM-kinase II (CAMK2) is a prominent kinase in the central nervous system that may function in long-term potentiation and neurotransmitter release. Member of the NMDAR signaling complex in	2.10

	excitatory synapses it may regulate NMDAR-dependent potentiation of the AMPAR and synaptic plasticity (By similarity). Phosphorylates transcription factor FOXO3 on 'Ser-298'. Activates FOXO3 transcriptional activity (By similarity); Belongs to the protein kinase superfamily.	
C21orf91	Chromosome 21 open reading frame 91; Plays a role in cortical progenitor cell proliferation and differentiation. Promotes dendritic spine development of post- migratory cortical projection neurons by modulating the beta- catenin signaling pathway; Belongs to the EURL family.	2.09
C1QL1	C1q and tumor necrosis factor-related protein 14; May regulate the number of excitatory synapses that are formed on hippocampus neurons. Has no effect on inhibitory synapses (By similarity); C1q and TNF related	2.05
CDKN2B	Cyclin-dependent kinase inhibitor 2B (p15, inhibits CDK4); Interacts strongly with CDK4 and CDK6. Potent inhibitor. Potential effector of TGF-beta induced cell cycle arrest; Belongs to the CDKN2 cyclin-dependent kinase inhibitor family.	2.01
IFNA21	Interferon, alpha 21; Produced by macrophages, IFN-alpha have antiviral activities. Interferon stimulates the production of two enzymes: a protein kinase and an oligoadenylate synthetase; Interferons	1.99
ADRB2	Adrenoceptor beta 2, surface; Beta-adrenergic receptors mediate the catecholamine- induced activation of adenylate cyclase through the action of G proteins. The beta-2-adrenergic receptor binds epinephrine with an approximately 30-fold greater affinity than it does norepinephrine; Belongs to the G-protein coupled receptor 1 family. Adrenergic receptor subfamily. ADRB2 sub-subfamily.	1.98
PGF	Placental growth factor; Growth factor active in angiogenesis and endothelial cell growth, stimulating their proliferation and migration. It binds to the receptor FLT1/VEGFR-1. Isoform PlGF-2 binds NRP1/neuropilin-1 and NRP2/neuropilin-2 in a heparin-dependent manner. Also promotes cell tumor growth.	1.97
KLF6	Suppressor of tumorigenicity 12 protein; Transcriptional activator (By similarity). Binds a GC box motif. Could play a role in B-cell growth and development; Belongs to the krueppel C2H2-type zinc-finger protein family.	1.97
MED1	Thyroid hormone receptor-associated protein complex 220 kDa component; Component of the Mediator complex, a coactivator involved in the regulated transcription of nearly all RNA polymerase II-dependent genes. Mediator functions as a bridge to convey information from gene-specific regulatory proteins to the basal RNA polymerase II transcription machinery.	1.96
SMARCB1	SWI/SNF related, matrix associated, actin dependent regulator of chromatin, subfamily b, member 1; Core component of the BAF (hSWI/SNF) complex. This ATP- dependent chromatin-remodeling complex plays important roles in cell proliferation and differentiation, in cellular antiviral activities and inhibition of tumor formation. The BAF complex is able to create a stable, altered form of chromatin that constrains fewer negative supercoils than normal.	1.91

ROS1	ROS proto-oncogene 1, receptor tyrosine kinase; Orphan receptor tyrosine kinase (RTK) that plays a role in epithelial cell differentiation and regionalization of the proximal epididymal epithelium. May activate several downstream signaling pathways related to cell differentiation, proliferation, growth, and survival including the PI3 kinase-mTOR signaling pathway. Mediates the phosphorylation of PTPN11, an activator of this pathway.	1.87
KDM5B	Retinoblastoma-binding protein 2 homolog 1; Histone demethylase that demethylates 'Lys-4' of histone H3, thereby playing a central role in histone code. Does not demethylate histone H3 'Lys-9' or H3 'Lys-27'. Demethylates trimethylated, dimethylated and monomethylated H3 'Lys-4'. Acts as a transcriptional corepressor for FOXG1B and PAX9. Favors the proliferation of breast cancer cells by repressing tumor suppressor genes such as BRCA1 and HOXA5.	1.87
MYBL1	V-myb avian myeloblastosis viral oncogene homolog-like 1; Transcription factor that specifically recognizes the sequence 5'-YAAC[GT]G-3'. Acts as a master regulator of male meiosis by promoting expression of piRNAs: activates expression of both piRNA precursor RNAs and expression of protein-coding genes involved in piRNA metabolism (By similarity).	1.86
ANGPT2	Angiopoietin 2; Binds to TEK/TIE2, competing for the ANGPT1 binding site, and modulating ANGPT1 signaling. Can induce tyrosine phosphorylation of TEK/TIE2 in the absence of ANGPT1. In the absence of angiogenic inducers, such as VEGF, ANGPT2-mediated loosening of cell-matrix contacts may induce endothelial cell apoptosis with consequent vascular regression. In concert with VEGF, it may facilitate endothelial cell migration and proliferation, thus serving as a permissive angiogenic signal.	1.86
FGF8	Fibroblast growth factor 8 (androgen-induced); Plays an important role in the regulation of embryonic development, cell proliferation, cell differentiation and cell migration. Required for normal brain, eye, ear, and limb development during embryogenesis. Required for normal development of the gonadotropin-releasing hormone (GnRH) neuronal system.	1.82
DCC	Immunoglobulin superfamily DCC subclass member 1; Receptor for netrin required for axon guidance. Mediates axon attraction of neuronal growth cones in the developing nervous system upon ligand binding. Its association with UNC5 proteins may trigger signaling for axon repulsion.	1.82
ETV7	ETS translocation variant 7; Transcriptional repressor; binds to the DNA sequence 5'- CCGGAAGT-3'. Isoform A does not seem to have a repressor activity. Isoform C does not seem to have a repressor activity; Belongs to the ETS family.	1.81
TP73	P53-like transcription factor; Participates in the apoptotic response to DNA damage. Isoforms containing the transactivation domain are pro-apoptotic, isoforms lacking the domain are anti-apoptotic and block the function of p53 and transactivating p73 isoforms. May be a tumor suppressor protein.	1.76
PRKCZ	Protein kinase C zeta type; Calcium- and diacylglycerol-independent serine/threonine-protein kinase that functions in phosphatidylinositol 3-	1.75

	kinase (PI3K) pathway and mitogen-activated protein (MAP) kinase cascade, and is involved in NF-kappa-B activation, mitogenic signaling, cell proliferation, cell polarity, inflammatory response and maintenance of long-term potentiation (LTP).	
GRB7	Epidermal growth factor receptor GRB-7; Adapter protein that interacts with the cytoplasmic domain of numerous receptor kinases and modulates down-stream signaling. Promotes activation of down-stream protein kinases, including STAT3, AKT1, MAPK1 and/or MAPK3. Promotes activation of HRAS. Plays a role in signal transduction in response to EGF. Plays a role in the regulation of cell proliferation and cell migration.	1.73
FGFR1	Basic fibroblast growth factor receptor 1; Tyrosine-protein kinase that acts as cell-surface receptor for fibroblast growth factors and plays an essential role in the regulation of embryonic development, cell proliferation, differentiation, and migration.	1.72
USH2A	Usher syndrome 2A (autosomal recessive, mild); Involved in hearing and vision; Fibronectin type III domain containing	1.70
SPINK1	Serine peptidase inhibitor, Kazal type 1; Serine protease inhibitor which exhibits anti-trypsin activity. In the pancreas, protects against trypsin-catalyzed premature activation of zymogens (By similarity); Serine peptidase inhibitors, Kazal type	1.68
TCF12	Class B basic helix-loop-helix protein 20; Transcriptional regulator. Involved in the initiation of neuronal differentiation. Activates transcription by binding to the E box (5'-CANNTG-3'); Basic helix-loop-helix proteins	1.66
CDKN1A	Cyclin-dependent kinase inhibitor 1A (p21, Cip1); May be involved in p53/TP53 mediated inhibition of cellular proliferation in response to DNA damage. Binds to and inhibits cyclin-dependent kinase activity, preventing phosphorylation of critical cyclin-dependent kinase substrates and blocking cell cycle progression. Functions in the nuclear localization and assembly of cyclin D-CDK4 complex and promotes its kinase activity towards RB1.	1.65
CD9	Cell growth-inhibiting gene 2 protein; Involved in platelet activation and aggregation. Regulates paranodal junction formation. Involved in cell adhesion, cell motility and tumor metastasis.	1.63
BTK	Bruton agammaglobulinemia tyrosine kinase; Non-receptor tyrosine kinase indispensable for B lymphocyte development, differentiation and signaling. Binding of antigen to the B-cell antigen receptor (BCR) triggers signaling that ultimately leads to B-cell activation. After BCR engagement and activation at the plasma membrane, phosphorylates PLCG2 at several sites, igniting the downstream signaling pathway through calcium mobilization, followed by activation of the protein kinase C (PKC) family members. PLCG2 phosphorylation is performed in close cooperation with the adapter protein B-cell linker protein BLNK.	1.59
SMAD2	Mothers against decapentaplegic homolog 2; Receptor-regulated SMAD (R-SMAD) that is an intracellular signal transducer and transcriptional modulator activated by TGF-beta (transforming growth factor) and activin type 1 receptor kinases. Binds the TRE element in the promoter region of many genes that are regulated by TGF-beta and, on formation	1.57

	of the SMAD2/SMAD4 complex, activates transcription. May act as a tumor suppressor in colorectal carcinoma.	
IL1B	Interleukin 1, beta; Potent proinflammatory cytokine. Initially discovered as the major endogenous pyrogen, induces prostaglandin synthesis, neutrophil influx and activation, T-cell activation and cytokine production, B-cell activation and antibody production, and fibroblast proliferation and collagen production. Promotes Th17 differentiation of T-cells.	1.55
DYNLT1	T-complex testis-specific protein 1 homolog; Acts as one of several non-catalytic accessory components of the cytoplasmic dynein 1 complex that are thought to be involved in linking dynein to cargos and to adapter proteins that regulate dynein function. Cytoplasmic dynein 1 acts as a motor for the intracellular retrograde motility of vesicles and organelles along microtubules. Binds to transport cargos and is involved in apical cargo transport such as rhodopsin-bearing vesicles in polarized epithelia.	1.52
**GO:0042127 Regulation of cell population proliferation**		
**Symbol**	**Description**	**Z-score**
TNFRSF11A	Tumor necrosis factor receptor superfamily, member 11a, NFKB activator; Receptor for TNFSF11/RANKL/TRANCE/OPGL; essential for RANKL-mediated osteoclastogenesis. Involved in the regulation of interactions between T-cells and dendritic cells; CD molecules	3.68
CCND1	B-cell lymphoma 1 protein; Regulatory component of the cyclin D1-CDK4 (DC) complex that phosphorylates and inhibits members of the retinoblastoma (RB) protein family including RB1 and regulates the cell-cycle during G(1)/S transition. Cyclin D-CDK4 complexes are major integrators of various mitogenic and antimitogenic signals.	3.26
CD74	CD74 molecule, major histocompatibility complex, class II invariant chain; Plays a critical role in MHC class II antigen processing by stabilizing peptide-free class II alpha/beta heterodimers in a complex soon after their synthesis and directing transport of the complex from the endoplasmic reticulum to the endosomal/lysosomal system where the antigen processing and binding of antigenic peptides to MHC class II takes place. Serves as cell surface receptor for the cytokine MIF; CD molecules	3.00
EIF4G1	Eukaryotic translation initiation factor 4 gamma, 1; Component of the protein complex eIF4F, which is involved in the recognition of the mRNA cap, ATP-dependent unwinding of 5'-terminal secondary structure and recruitment of mRNA to the ribosome; Parkinson disease associated genes	2.99
VEGFA	Vascular endothelial growth factor A; Growth factor active in angiogenesis, vasculogenesis and endothelial cell growth. Induces endothelial cell proliferation, promotes cell migration, inhibits apoptosis, and induces permeabilization of blood vessels. Binds to the FLT1/VEGFR1 and KDR/VEGFR2 receptors, heparan sulfate and heparin.	2.96
TNFAIP3	Tumor necrosis factor, alpha-induced protein 3; Ubiquitin-editing enzyme that contains both ubiquitin ligase and deubiquitinase activities. Involved in immune and inflammatory responses signaled by cytokines, such as TNF-alpha and IL-1 beta, or pathogens via Toll-like receptors	2.94

	(TLRs) through terminating NF-kappa-B activity. Essential component of a ubiquitin-editing protein complex, comprising also RNF11, ITCH and TAX1BP1, that ensures the transient nature of inflammatory signaling pathways.	
CDH13	Truncated cadherin; Cadherins are calcium-dependent cell adhesion proteins. They preferentially interact with themselves in a homophilic manner in connecting cells; cadherins may thus contribute to the sorting of heterogeneous cell types. May act as a negative regulator of neural cell growth.	2.91
PDCD6	Apoptosis-linked gene 2 protein homolog; Calcium sensor that plays a key role in processes such as endoplasmic reticulum (ER)-Golgi vesicular transport, endosomal biogenesis, or membrane repair. Acts as an adapter that bridges unrelated proteins or stabilizes weak protein-protein complexes in response to calcium: calcium-binding triggers exposure of apolar surface, promoting interaction with different sets of proteins thanks to 3 different hydrophobic pockets, leading to translocation to membranes.	2.48
PLAC8	Placenta-specific gene 8 protein; Placenta specific 8	2.30
RPS3	Small ribosomal subunit protein uS3; Involved in translation as a component of the 40S small ribosomal subunit. Has endonuclease activity and plays a role in repair of damaged DNA. Cleaves phosphodiester bonds of DNAs containing altered bases with broad specificity and cleaves supercoiled DNA more efficiently than relaxed DNA.	2.19
JUND	Transcription factor jun-D; Transcription factor binding AP-1 sites; Belongs to the bZIP family. Jun subfamily.	2.18
IGF2	Insulin-like growth factor 2 (somatomedin A); The insulin-like growth factors possess growth-promoting activity. Major fetal growth hormone in mammals. Plays a key role in regulating fetoplacental development. IGF-II is influenced by placental lactogen. Also involved in tissue differentiation.	2.16
ANXA2	Placental anticoagulant protein IV; Calcium-regulated membrane-binding protein whose affinity for calcium is greatly enhanced by anionic phospholipids. It binds two calcium ions with high affinity. May be involved in heat-stress response. Inhibits PCSK9-enhanced LDLR degradation, probably reduces PCSK9 protein levels via a translational mechanism but also competes with LDLR for binding with PCSK9.	2.13
BAD	Bcl-xL/Bcl-2-associated death promoter; Promotes cell death. Successfully competes for the binding to Bcl-X(L), Bcl-2 and Bcl-W, thereby affecting the level of heterodimerization of these proteins with BAX. Can reverse the death repressor activity of Bcl-X(L), but not that of Bcl-2 (By similarity). Appears to act as a link between growth factor receptor signaling and the apoptotic pathways.	2.12
TNFRSF4	TAX transcriptionally-activated glycoprotein 1 receptor; Receptor for TNFSF4/OX40L/GP34. Is a costimulatory molecule implicated in long-term T-cell immunity; CD molecules	2.05

AVP	Vasopressin-neurophysin 2-copeptin; Neurophysin 2 specifically binds vasopressin; Endogenous ligands	2.03
CDKN2B	Cyclin-dependent kinase inhibitor 2B (p15, inhibits CDK4); Interacts strongly with CDK4 and CDK6. Potent inhibitor. Potential effector of TGF-beta induced cell cycle arrest; Belongs to the CDKN2 cyclin-dependent kinase inhibitor family.	2.01
PGF	Placental growth factor; Growth factor active in angiogenesis and endothelial cell growth, stimulating their proliferation and migration. It binds to the receptor FLT1/VEGFR-1. Isoform PlGF-2 binds NRP1/neuropilin-1 and NRP2/neuropilin-2 in a heparin-dependent manner. Also promotes cell tumor growth.	1.97
MED1	Thyroid hormone receptor-associated protein complex 220 kDa component; Component of the Mediator complex, a coactivator involved in the regulated transcription of nearly all RNA polymerase II-dependent genes. Mediator functions as a bridge to convey information from gene-specific regulatory proteins to the basal RNA polymerase II transcription machinery.	1.96
FANCA	Fanconi anemia, complementation group A; DNA repair protein that may operate in a post replication repair or a cell cycle checkpoint function. May be involved in interstrand DNA cross-link repair and in the maintenance of normal chromosome stability; Fanconi anemia complementation groups	1.94
SMARCB1	SWI/SNF related, matrix associated, actin dependent regulator of chromatin, subfamily b, member 1; Core component of the BAF (hSWI/SNF) complex. This ATP- dependent chromatin-remodeling complex plays important roles in cell proliferation and differentiation, in cellular antiviral activities and inhibition of tumor formation. The BAF complex is able to create a stable, altered form of chromatin that constrains fewer negative supercoils than normal.	1.91
KDM5B	Retinoblastoma-binding protein 2 homolog 1; Histone demethylase that demethylates 'Lys-4' of histone H3, thereby playing a central role in histone code. Does not demethylate histone H3 'Lys-9' or H3 'Lys-27'. Demethylates trimethylated, dimethylated and monomethylated H3 'Lys-4'. Acts as a transcriptional corepressor for FOXG1B and PAX9. Favors the proliferation of breast cancer cells by repressing tumor suppressor genes such as BRCA1 and HOXA5.	1.87
FGF8	Fibroblast growth factor 8 (androgen-induced); Plays an important role in the regulation of embryonic development, cell proliferation, cell differentiation and cell migration. Required for normal brain, eye, ear, and limb development during embryogenesis. Required for normal development of the gonadotropin-releasing hormone (GnRH) neuronal system.	1.82
HRAS	Harvey rat sarcoma viral oncogene homolog; Involved in the activation of Ras protein signal transduction. Ras proteins bind GDP/GTP and possess intrinsic GTPase activity; Belongs to the small GTPase superfamily. Ras family.	1.77
TP73	P53-like transcription factor; Participates in the apoptotic response to DNA damage. Isoforms containing the transactivation domain are pro-	1.76

	apoptotic, isoforms lacking the domain are anti-apoptotic and block the function of p53 and transactivating p73 isoforms. May be a tumor suppressor protein.	
PRKCZ	Protein kinase C zeta type; Calcium- and diacylglycerol-independent serine/threonine-protein kinase that functions in phosphatidylinositol 3-kinase (PI3K) pathway and mitogen-activated protein (MAP) kinase cascade, and is involved in NF-kappa-B activation, mitogenic signaling, cell proliferation, cell polarity, inflammatory response and maintenance of long-term potentiation (LTP).	1.75
TSPAN32	Tetraspanin 32; Belongs to the tetraspanin (TM4SF) family.	1.73
FGFR1	Basic fibroblast growth factor receptor 1; Tyrosine-protein kinase that acts as cell-surface receptor for fibroblast growth factors and plays an essential role in the regulation of embryonic development, cell proliferation, differentiation, and migration.	1.72
MBD2	Methyl-CpG-binding domain protein 2; Binds CpG islands in promoters where the DNA is methylated at position 5 of cytosine within CpG dinucleotides. Binds hemimethylated DNA as well. Recruits histone deacetylases and DNA methyltransferases.	1.71
CDKN1A	Cyclin-dependent kinase inhibitor 1A (p21, Cip1); May be involved in p53/TP53 mediated inhibition of cellular proliferation in response to DNA damage. Binds to and inhibits cyclin-dependent kinase activity, preventing phosphorylation of critical cyclin-dependent kinase substrates and blocking cell cycle progression. Functions in the nuclear localization and assembly of cyclin D-CDK4 complex and promotes its kinase activity towards RB1.	1.65
ASPH	Aspartyl/asparaginyl beta-hydroxylase; Isoform 1: specifically hydroxylates an Asp or Asn residue in certain epidermal growth factor-like (EGF) domains of a number of proteins; Belongs to the aspartyl/asparaginyl beta-hydroxylase family.	1.63
CD9	Cell growth-inhibiting gene 2 protein; Involved in platelet activation and aggregation. Regulates paranodal junction formation. Involved in cell adhesion, cell motility and tumor metastasis. Required for sperm-egg fusion; Belongs to the tetraspanin (TM4SF) family.	1.63
BTK	Bruton agammaglobulinemia tyrosine kinase; Non-receptor tyrosine kinase indispensable for B lymphocyte development, differentiation and signaling. Binding of antigen to the B-cell antigen receptor (BCR) triggers signaling that ultimately leads to B-cell activation. After BCR engagement and activation at the plasma membrane, phosphorylates PLCG2 at several sites, igniting the downstream signaling pathway through calcium mobilization, followed by activation of the protein kinase C (PKC) family members. PLCG2 phosphorylation is performed in close cooperation with the adapter protein B-cell linker protein BLNK.	1.59
PTPRJ	Protein tyrosine phosphatase, receptor type, J; Tyrosine phosphatase which dephosphorylates or contributes to the dephosphorylation of CTNND1, FLT3, PDGFRB, MET, RET (variant MEN2A), KDR, LYN, SRC, MAPK1, MAPK3, EGFR, TJP1, OCLN, PIK3R1 and PIK3R2. Plays a role in cell adhesion, migration, proliferation, and differentiation. Involved in	1.59

	vascular development. Regulator of macrophage adhesion and spreading.	
SMAD2	Mothers against decapentaplegic homolog 2; Receptor-regulated SMAD (R-SMAD) that is an intracellular signal transducer and transcriptional modulator activated by TGF-beta (transforming growth factor) and activin type 1 receptor kinases. Binds the TRE element in the promoter region of many genes that are regulated by TGF-beta and, on formation of the SMAD2/SMAD4 complex, activates transcription.	1.57
ZFP36	Growth factor-inducible nuclear protein NUP475; Zinc-finger RNA-binding protein that destabilizes several cytoplasmic AU-rich element (ARE)-containing mRNA transcripts by promoting their poly(A) tail removal or deadenylation, and hence provide a mechanism for attenuating protein synthesis. Acts as an 3'-untranslated region (UTR) ARE mRNA-binding adapter protein to communicate signaling events to the mRNA decay machinery.	1.56
IL1B	Interleukin 1, beta; Potent proinflammatory cytokine. Initially discovered as the major endogenous pyrogen, induces prostaglandin synthesis, neutrophil influx and activation, T-cell activation and cytokine production, B-cell activation and antibody production, and fibroblast proliferation and collagen production. Promotes Th17 differentiation of T-cells.	1.55
TCL1A	T-cell leukemia/lymphoma protein 1A; Enhances the phosphorylation and activation of AKT1, AKT2 and AKT3. Promotes nuclear translocation of AKT1. Enhances cell proliferation, stabilizes mitochondrial membrane potential, and promotes cell survival; Belongs to the TCL1 family.	1.55
APC	Adenomatous polyposis coli protein; Tumor suppressor. Promotes rapid degradation of CTNNB1 and participates in Wnt signaling as a negative regulator. APC activity is correlated with its phosphorylation state.	1.54
HYAL1	Hyaluronoglucosaminidase 1; May have a role in promoting tumor progression. May block the TGFB1-enhanced cell growth.	1.53
**GO:0016477: Cell migration**		
**Symbol**	**Description**	**Z-score**
TNFRSF11A	Tumor necrosis factor receptor superfamily, member 11a, NFKB activator; Receptor for TNFSF11/RANKL/TRANCE/OPGL; essential for RANKL-mediated osteoclastogenesis. Involved in the regulation of interactions between T-cells and dendritic cells; CD molecules	3.67780662
CD74	CD74 molecule, major histocompatibility complex, class II invariant chain; Plays a critical role in MHC class II antigen processing by stabilizing peptide-free class II alpha/beta heterodimers in a complex soon after their synthesis and directing transport of the complex from the endoplasmic reticulum to the endosomal/lysosomal system where the antigen processing and binding of antigenic peptides to MHC class II takes place. Serves as cell surface receptor for the cytokine MIF; CD molecules	2.99539557
VEGFA	Vascular endothelial growth factor A; Growth factor active in angiogenesis, vasculogenesis and endothelial cell growth. Induces endothelial cell proliferation, promotes cell migration, inhibits apoptosis,	2.95721153

	and induces permeabilization of blood vessels. Binds to the FLT1/VEGFR1 and KDR/VEGFR2 receptors, heparan sulfate and heparin.	
CDH13	Truncated cadherin; Cadherins are calcium-dependent cell adhesion proteins. They preferentially interact with themselves in a homophilic manner in connecting cells; cadherins may thus contribute to the sorting of heterogeneous cell types. May act as a negative regulator of neural cell growth.	2.90937722
AXL	Tyrosine-protein kinase receptor UFO; Receptor tyrosine kinase that transduces signals from the extracellular matrix into the cytoplasm by binding growth factor GAS6 and which is thus regulating many physiological processes including cell survival, cell proliferation, migration, and differentiation. Ligand binding at the cell surface induces dimerization and autophosphorylation of AXL.	2.63953376
TNFAIP1	BTB/POZ domain-containing adapter for CUL3-mediated RhoA degradation protein 2; Substrate-specific adapter of a BCR (BTB-CUL3-RBX1) E3 ubiquitin-protein ligase complex involved in regulation of cytoskeleton structure. The BCR(TNFAIP1) E3 ubiquitin ligase complex mediates the ubiquitination of RHOA, leading to its degradation by the proteasome, thereby regulating the actin cytoskeleton and cell migration. Its interaction with RHOB may regulate apoptosis.	2.49216276
ANGPT2	Angiopoietin 2; Binds to TEK/TIE2, competing for the ANGPT1 binding site, and modulating ANGPT1 signaling. Can induce tyrosine phosphorylation of TEK/TIE2 in the absence of ANGPT1. In the absence of angiogenic inducers, such as VEGF, ANGPT2-mediated loosening of cell-matrix contacts may induce endothelial cell apoptosis with consequent vascular regression. In concert with VEGF, it may facilitate endothelial cell migration and proliferation, thus serving as a permissive angiogenic signal.	1.85567559
FGF8	Fibroblast growth factor 8 (androgen-induced); Plays an important role in the regulation of embryonic development, cell proliferation, cell differentiation and cell migration. Required for normal brain, eye, ear, and limb development during embryogenesis. Required for normal development of the gonadotropin-releasing hormone (GnRH) neuronal system.	1.8224645
DCC	Immunoglobulin superfamily DCC subclass member 1; Receptor for netrin required for axon guidance. Mediates axon attraction of neuronal growth cones in the developing nervous system upon ligand binding. Its association with UNC5 proteins may trigger signaling for axon repulsion. It also acts as a dependence receptor required for apoptosis induction when not associated with netrin ligand.	1.81871284
PRKCZ	Protein kinase C zeta type; Calcium- and diacylglycerol-independent serine/threonine-protein kinase that functions in phosphatidylinositol 3-kinase (PI3K) pathway and mitogen-activated protein (MAP) kinase cascade, and is involved in NF-kappa-B activation, mitogenic signaling, cell proliferation, cell polarity, inflammatory response and maintenance of long-term potentiation (LTP). Upon lipopolysaccharide (LPS) treatment in macrophages, or following mitogenic stimuli, functions downstream of PI3K to activate MAP2K1/MEK1-MAPK1/ERK2 signaling cascade	1.74922215

	independently of RAF1 activation. Required for insulin-dependent activation of AKT3 but may function as an adapter rather than a direct activator.	
GRB7	Epidermal growth factor receptor GRB-7; Adapter protein that interacts with the cytoplasmic domain of numerous receptor kinases and modulates down-stream signaling. Promotes activation of down-stream protein kinases, including STAT3, AKT1, MAPK1 and/or MAPK3. Promotes activation of HRAS. Plays a role in signal transduction in response to EGF. Plays a role in the regulation of cell proliferation and cell migration.	1.72744231
FGFR1	Basic fibroblast growth factor receptor 1; Tyrosine-protein kinase that acts as cell-surface receptor for fibroblast growth factors and plays an essential role in the regulation of embryonic development, cell proliferation, differentiation, and migration. Required for normal mesoderm patterning and correct axial organization during embryonic development, normal skeletogenesis and normal development of the gonadotropin-releasing hormone (GnRH) neuronal system. Phosphorylates PLCG1, FRS2, GAB1 and SHB.	1.7239346
CXCR5	Chemokine (C-X-C motif) receptor 5; Cytokine receptor that binds to B-lymphocyte chemoattractant (BLC). Involved in B-cell migration into B-cell follicles of spleen and Peyer patches but not into those of mesenteric or peripheral lymph nodes. May have a regulatory function in Burkitt lymphoma (BL) lymphomagenesis and/or B-cell differentiation; C-X-C motif chemokine receptors	1.64279282
IL1B	Interleukin 1, beta; Potent proinflammatory cytokine. Initially discovered as the major endogenous pyrogen, induces prostaglandin synthesis, neutrophil influx and activation, T-cell activation and cytokine production, B-cell activation and antibody production, and fibroblast proliferation and collagen production. Promotes Th17 differentiation of T-cells.	1.55413196
APC	Adenomatous polyposis coli protein; Tumor suppressor. Promotes rapid degradation of CTNNB1 and participates in Wnt signaling as a negative regulator. APC activity is correlated with its phosphorylation state. Activates the GEF activity of SPATA13 and ARHGEF4. Plays a role in hepatocyte growth factor (HGF)-induced cell migration.	1.5412017
**Down-Regulated Transcripts after 24 h of UTP**		
**GO: 0042493 Response to drug**		
**Symbol**	**Description**	**Z-score**
SLC22A3	Solute carrier family 22 (organic cation transporter), member 3; Mediates potential-dependent transport of a variety of organic cations. May play a significant role in the disposition of cationic neurotoxins and neurotransmitters in the brain; Belongs to the major facilitator (TC 2.A.1) superfamily. Organic cation transporter (TC 2.A.1.19) family.	-3.64
CREB1	Cyclic AMP-responsive element-binding protein 1; Phosphorylation-dependent transcription factor that stimulates transcription upon binding to the DNA cAMP response element (CRE), a sequence present in many viral and cellular promoters. Transcription activation is enhanced by the TORC coactivators which act independently of Ser-133	-3.36

	phosphorylation. Involved in different cellular processes including the synchronization of circadian rhythmicity and the differentiation of adipose cells; Basic leucine zipper proteins	
NFKBIA	Nuclear factor of kappa light polypeptide gene enhancer in B-cells inhibitor, alpha; Inhibits the activity of dimeric NF-kappa-B/REL complexes by trapping REL dimers in the cytoplasm through masking of their nuclear localization signals. On cellular stimulation by immune and proinflammatory responses, becomes phosphorylated promoting ubiquitination and degradation, enabling the dimeric RELA to translocate to the nucleus and activate transcription.	-3.06
ANXA1	Phospholipase A2 inhibitory protein; Plays important roles in the innate immune response as effector of glucocorticoid-mediated responses and regulator of the inflammatory process. Has anti-inflammatory activity. Plays a role in glucocorticoid-mediated down- regulation of the early phase of the inflammatory response (By similarity).	-2.52
NR4A3	Nuclear receptor subfamily 4, group A, member 3; Transcriptional activator that binds to regulatory elements in promoter regions in a cell- and response element (target)-specific manner. Induces gene expression by binding as monomers to the NR4A1 response element (NBRE) 5'-AAAAGGTCA-3' site and as homodimers to the Nur response element (NurRE) site in the promoter of their regulated target genes (By similarity). Plays a role in the regulation of proliferation, survival, and differentiation of many different cell types and also in metabolism and inflammation.	-2.21
CLDN1	Senescence-associated epithelial membrane protein; Claudins function as major constituents of the tight junction complexes that regulate the permeability of epithelia. While some claudin family members play essential roles in the formation of impermeable barriers, others mediate the permeability to ions and small molecules. Often, several claudin family members are co-expressed and interact with each other, and this determines the overall permeability.	-2.10
P2RY2	Purinergic receptor P2Y, G-protein coupled, 2; Receptor for ATP and UTP coupled to G-proteins that activate a phosphatidylinositol-calcium second messenger system. The affinity range is UTP = ATP > ATP-gamma-S >> 2-methylthio-ATP = ADP; P2Y receptors	-2.07
PPARG	Peroxisome proliferator-activated receptor gamma; Nuclear receptor that binds peroxisome proliferators such as hypolipidemic drugs and fatty acids. Once activated by a ligand, the nuclear receptor binds to DNA specific PPAR response elements (PPRE) and modulates the transcription of its target genes, such as acyl-CoA oxidase. It therefore controls the peroxisomal beta-oxidation pathway of fatty acids. Key regulator of adipocyte differentiation and glucose homeostasis.	-2.04
CYP4B1	Cytochrome P450, family 4, subfamily B, polypeptide 1; Cytochromes P450 are a group of heme-thiolate monooxygenases. In liver microsomes, this enzyme is involved in an NADPH-dependent electron transport pathway. It oxidizes a variety of structurally unrelated compounds, including steroids, fatty acids, and xenobiotics.	-2.00

PTH	Parathyroid hormone; PTH elevates calcium level by dissolving the salts in bone and preventing their renal excretion. Stimulates [1-14C]-2-deoxy-D-glucose (2DG) transport and glycogen synthesis in osteoblastic cells; Belongs to the parathyroid hormone family.	-1.98
PDGFB	Platelet-derived growth factor beta polypeptide; Growth factor that plays an essential role in the regulation of embryonic development, cell proliferation, cell migration, survival, and chemotaxis. Potent mitogen for cells of mesenchymal origin. Required for normal proliferation and recruitment of pericytes and vascular smooth muscle cells in the central nervous system, skin, lung, heart, and placenta.	-1.90
HDAC8	Histone deacetylase 8; Responsible for the deacetylation of lysine residues on the N-terminal part of the core histones (H2A, H2B, H3 and H4). Histone deacetylation gives a tag for epigenetic repression and plays an important role in transcriptional regulation, cell cycle progression and developmental events. Histone deacetylases act via the formation of large multiprotein complexes.	-1.87
PTEN	Mutated in multiple advanced cancers 1, Tumor suppressor. Acts as a dual-specificity protein phosphatase, dephosphorylating tyrosine-, serine- and threonine- phosphorylated proteins. Also acts as a lipid phosphatase, removing the phosphate in the D3 position of the inositol ring from phosphatidylinositol 3,4,5-trisphosphate, phosphatidylinositol 3,4-diphosphate, phosphatidylinositol 3- phosphate and inositol 1,3,4,5-tetrakisphosphate with order of substrate preference in vitro PtdIns(3,4,5)P3 > PtdIns(3,4)P2 > PtdIns3P > Ins(1,3,4,5)P4. positioning, dendritic development and synapse formation.	-1.79
WFDC1	WAP four-disulfide core domain protein 1; Has growth inhibitory activity; WAP four-disulfide core domain containing	-1.73
TAF1	TAF1 RNA polymerase II, TATA box binding protein (TBP)-associated factor, 250kDa; Largest component and core scaffold of the TFIID basal transcription factor complex. Contains novel N- and C-terminal Ser/Thr kinase domains which can autophosphorylate or transphosphorylate other transcription factors. Phosphorylates TP53 on 'Thr-55' which leads to MDM2- mediated degradation of TP53. Phosphorylates GTF2A1 and GTF2F1 on Ser residues. Possesses DNA-binding activity.	-1.73
CASP8	Caspase 8, apoptosis-related cysteine peptidase; Most upstream protease of the activation cascade of caspases responsible for the TNFRSF6/FAS mediated and TNFRSF1A induced cell death. Binding to the adapter molecule FADD recruits it to either receptor. The resulting aggregate called death- inducing signaling complex (DISC) performs CASP8 proteolytic activation.	-1.71
MYB	V-myb avian myeloblastosis viral oncogene homolog; Transcriptional activator; DNA-binding protein that specifically recognize the sequence 5'-YAAC[GT]G-3'. Plays an important role in the control of proliferation and differentiation of hematopoietic progenitor cells; Myb/SANT domain containing	-1.58
IL13	Interleukin 13; Cytokine. Inhibits inflammatory cytokine production. Synergizes with IL2 in regulating interferon-gamma synthesis. May be critical in regulating inflammatory and immune responses.	-1.57

XRCC5	ATP-dependent DNA helicase II 80 kDa subunit; Single-stranded DNA-dependent ATP-dependent helicase. Has a role in chromosome translocation. The DNA helicase II complex binds preferentially to fork-like ends of double-stranded DNA in a cell cycle-dependent manner.	-1.56
LYN	V-yes-1 Yamaguchi sarcoma viral related oncogene homolog; Non-receptor tyrosine-protein kinase that transmits signals from cell surface receptors and plays an important role in the regulation of innate and adaptive immune responses, hematopoiesis, responses to growth factors and cytokines, integrin signaling, but also responses to DNA damage and genotoxic agents.	-1.50
**GO:0012501 Programmed cell death**		
**Symbol**	**Description**	**Z-score**
CTSV	Cathepsin L2; Cysteine protease. May have an important role in corneal physiology; Belongs to the peptidase C1 family.	-3.44
NFKBIA	Nuclear factor of kappa light polypeptide gene enhancer in B-cells inhibitor, alpha; Inhibits the activity of dimeric NF-kappa-B/REL complexes by trapping REL dimers in the cytoplasm through masking of their nuclear localization signals. On cellular stimulation by immune and proinflammatory responses, becomes phosphorylated promoting ubiquitination and degradation, enabling the dimeric RELA to translocate to the nucleus and activate transcription.	-3.06
CASP10	FAS-associated death domain protein interleukin-1B-converting enzyme 2; Involved in the activation cascade of caspases responsible for apoptosis execution. Recruited to both Fas- and TNFR-1 receptors in a FADD dependent manner. May participate in the granzyme B apoptotic pathways.	-2.49
DAP3	Mitochondrial small ribosomal subunit protein mS29; Involved in mediating interferon-gamma-induced cell death; Belongs to the mitochondrion-specific ribosomal protein mS29 family.	-2.35
CITED1	Cbp/p300-interacting transactivator, with Glu/Asp-rich carboxy-terminal domain, 1; Transcriptional coactivator of the p300/CBP-mediated transcription complex. Enhances SMAD-mediated transcription by strengthening the functional link between the DNA-binding SMAD transcription factors and the p300/CBP transcription coactivator complex. Stimulates estrogen-dependent transactivation activity mediated by estrogen receptors signaling; stabilizes the interaction of estrogen receptor ESR1 and histone acetyltransferase EP300.	-2.08
PTH	Parathyroid hormone; PTH elevates calcium level by dissolving the salts in bone and preventing their renal excretion. Stimulates [1-14C]-2-deoxy-D-glucose (2DG) transport and glycogen synthesis in osteoblastic cells; Belongs to the parathyroid hormone family.	-1.98
DSP	250/210 kDa paraneoplastic pemphigus antigen; Major high molecular weight protein of desmosomes. Involved in the organization of the desmosomal cadherin- plakoglobin complexes into discrete plasma membrane domains and in the anchoring of intermediate filaments to the desmosomes; Belongs to the plakin or cytolinker family.	-1.90

PTEN	Mutated in multiple advanced cancers 1, Tumor suppressor. Acts as a dual-specificity protein phosphatase, dephosphorylating tyrosine-, serine- and threonine- phosphorylated proteins. Also acts as a lipid phosphatase, removing the phosphate in the D3 position of the inositol ring from phosphatidylinositol 3,4,5-trisphosphate, phosphatidylinositol 3,4-diphosphate, phosphatidylinositol 3- phosphate and inositol 1,3,4,5-tetrakisphosphate with order of substrate preference in vitro PtdIns(3,4,5)P3 > PtdIns(3,4)P2 > PtdIns3P > Ins(1,3,4,5)P4.	-1.79
TOPORS	Topoisomerase I binding, arginine/serine-rich, E3 ubiquitin protein ligase; Functions as an E3 ubiquitin-protein ligase and as an E3 SUMO1-protein ligase. Probable tumor suppressor involved in cell growth, cell proliferation and apoptosis that regulates p53/TP53 stability through ubiquitin-dependent degradation. May regulate chromatin modification through sumoylation of several chromatin modification-associated proteins.	-1.72
CASP8	Caspase 8, apoptosis-related cysteine peptidase; Most upstream protease of the activation cascade of caspases responsible for the TNFRSF6/FAS mediated and TNFRSF1A induced cell death. Binding to the adapter molecule FADD recruits it to either receptor. The resulting aggregate called death- inducing signaling complex (DISC) performs CASP8 proteolytic activation.	-1.71
HIC1	Zinc finger and BTB domain-containing protein 29; Transcriptional repressor. Recognizes and binds to the consensus sequence '5-[CG]NG[CG]GGGCA[CA]CC-3'. May act as a tumor suppressor.	-1.63
CLU	Testosterone-repressed prostate message 2; Isoform 1 functions as extracellular chaperone that prevents aggregation of nonnative proteins. Prevents stress- induced aggregation of blood plasma proteins. Inhibits formation of amyloid fibrils by APP, APOC2, B2M, CALCA, CSN3, SNCA and aggregation-prone LYZ variants (in vitro). Does not require ATP.	-1.56
MAX	Class D basic helix-loop-helix protein 4; Transcription regulator. Forms a sequence-specific DNA- binding protein complex with MYC or MAD which recognizes the core sequence 5'-CAC[GA]TG-3'. The MYC:MAX complex is a transcriptional activator, whereas the MAD:MAX complex is a repressor.	-1.55
CDSN	Corneodesmosin; Important for the epidermal barrier integrity.	-1.52
**GO:0045785 Positive regulation of cell adhesion**		
**Symbol**	**Description**	**Z-score**
ANXA1	Phospholipase A2 inhibitory protein; Plays important roles in the innate immune response as effector of glucocorticoid-mediated responses and regulator of the inflammatory process. Has anti-inflammatory activity. Plays a role in glucocorticoid-mediated down- regulation of the early phase of the inflammatory response (By similarity).	-2.52
CD44	GP90 lymphocyte homing/adhesion receptor; Receptor for hyaluronic acid (HA). Mediates cell-cell and cell-matrix interactions through its affinity for HA, and possibly also through its affinity for other ligands such as osteopontin, collagens, and matrix metalloproteinases (MMPs). Adhesion with HA plays an important role in cell migration, tumor growth	-2.39

	and progression. In cancer cells, may play an important role in invadopodia formation.	
NR4A3	Nuclear receptor subfamily 4, group A, member 3; Transcriptional activator that binds to regulatory elements in promoter regions in a cell- and response element (target)-specific manner. Induces gene expression by binding as monomers to the NR4A1 response element (NBRE) 5'-AAAAGGTCA-3' site and as homodimers to the Nur response element (NurRE) site in the promoter of their regulated target genes (By similarity). Plays a role in the regulation of proliferation, survival, and differentiation of many different cell types and also in metabolism and inflammation.	-2.21
VTN	Serum-spreading factor; Vitronectin is a cell adhesion and spreading factor found in serum and tissues. Vitronectin interact with glycosaminoglycans and proteoglycans. Is recognized by certain members of the integrin family and serves as a cell-to-substrate adhesion molecule. Inhibitor of the membrane-damaging effect of the terminal cytolytic complement pathway; Endogenous ligands	-1.90
HRG	Histidine-proline-rich glycoprotein; Plasma glycoprotein that binds a number of ligands such as heme, heparin, heparan sulfate, thrombospondin, plasminogen, and divalent metal ions. Binds heparin and heparin/glycosaminoglycans in a zinc-dependent manner. Binds heparan sulfate on the surface of liver, lung, kidney, and heart endothelial cells.	-1.72
MYB	V-myb avian myeloblastosis viral oncogene homolog; Transcriptional activator; DNA-binding protein that specifically recognize the sequence 5'-YAAC[GT]G-3'. Plays an important role in the control of proliferation and differentiation of hematopoietic progenitor cells; Myb/SANT domain containing	-1.58
ERBB2	V-erb-b2 avian erythroblastic leukemia viral oncogene homolog 2; Protein tyrosine kinase that is part of several cell surface receptor complexes, but that apparently needs a coreceptor for ligand binding. Essential component of a neuregulin-receptor complex, although neuregulins do not interact with it alone. GP30 is a potential ligand for this receptor. Regulates outgrowth and stabilization of peripheral microtubules (MTs).	-1.54
CXCL12	Pre-B cell growth-stimulating factor; Chemoattractant active on T-lymphocytes, monocytes, but not neutrophils. Activates the C-X-C chemokine receptor CXCR4 to induce a rapid and transient rise in the level of intracellular calcium ions and chemotaxis. Also binds to atypical chemokine receptor ACKR3, which activates the beta-arrestin pathway and acts as a scavenger receptor for SDF-1. SDF-1-beta(3-72) and SDF-1-alpha(3-67) show a reduced chemotactic activity.	-1.52
LYN	V-yes-1 Yamaguchi sarcoma viral related oncogene homolog; Non-receptor tyrosine-protein kinase that transmits signals from cell surface receptors and plays an important role in the regulation of innate and adaptive immune responses, hematopoiesis, responses to growth factors and cytokines, integrin signaling, but also responses to DNA damage and genotoxic agents.	-1.50

Up-Regulated Transcripts after 48 h of UTP		
**GO:0030154 Cell differentiation**		
**Symbol**	**Description**	**Z-score**
RPS6	Small ribosomal subunit protein eS6; May play an important role in controlling cell growth and proliferation through the selective translation of particular classes of mRNA; S ribosomal proteins	4.37
ETV7	ETS translocation variant 7; Transcriptional repressor; binds to the DNA sequence 5'- CCGGAAGT-3'. Isoform A does not seem to have a repressor activity. Isoform C does not seem to have a repressor activity; Belongs to the ETS family.	3.05
RPS19	Small ribosomal subunit protein eS19; Required for pre-rRNA processing and maturation of 40S ribosomal subunits; Belongs to the eukaryotic ribosomal protein eS19 family.	2.63
SMAD2	Mothers against decapentaplegic homolog 2; Receptor-regulated SMAD (R-SMAD) that is an intracellular signal transducer and transcriptional modulator activated by TGF-beta (transforming growth factor) and activin type 1 receptor kinases. Binds the TRE element in the promoter region of many genes that are regulated by TGF-beta and, on formation of the SMAD2/SMAD4 complex, activates transcription. May act as a tumor suppressor in colorectal carcinoma.	2.52
C19orf80	Refeeding-induced fat and liver protein; Hormone that acts as a blood lipid regulator by regulating serum triglyceride levels. May be involved in the metabolic transition between fasting and refeeding: required to direct fatty acids to adipose tissue for storage in the fed state (By similarity); Belongs to the ANGPTL8 family.	2.49
RAB8A	RAB8A, member RAS oncogene family; The small GTPases Rab are key regulators of intracellular membrane trafficking, from the formation of transport vesicles to their fusion with membranes. Rabs cycle between an inactive GDP-bound form and an active GTP-bound form that is able to recruit to membranes different sets of downstream effectors directly responsible for vesicle formation, movement, tethering and fusion. That Rab is involved in polarized vesicular trafficking and neurotransmitter release.	2.41
CAMK2A	Calcium/calmodulin-dependent protein kinase type II subunit alpha; CaM-kinase II (CAMK2) is a prominent kinase in the central nervous system that may function in long-term potentiation and neurotransmitter release. Member of the NMDAR signaling complex in excitatory synapses it may regulate NMDAR-dependent potentiation of the AMPAR and synaptic plasticity (By similarity). Phosphorylates transcription factor FOXO3 on 'Ser-298'. Activates FOXO3 transcriptional activity (By similarity); Belongs to the protein kinase superfamily.	2.30
L1CAM	Neural cell adhesion molecule L1; Neural cell adhesion molecule involved in the dynamics of cell adhesion and in the generation of transmembrane signals at tyrosine kinase receptors. During brain development, critical in multiple processes, including neuronal migration, axonal growth and fasciculation, and synaptogenesis. In the mature brain, plays a role in the	2.30

	dynamics of neuronal structure and function, including synaptic plasticity; CD molecules	
MAPT	Microtubule-associated protein tau; Promotes microtubule assembly and stability and might be involved in the establishment and maintenance of neuronal polarity. The C-terminus binds axonal microtubules while the N- terminus binds neural plasma membrane components, suggesting that tau functions as a linker protein between both. Axonal polarity is predetermined by TAU/MAPT localization (in the neuronal cell) in the domain of the cell body defined by the centrosome. The short isoforms allow plasticity of the cytoskeleton whereas the longer isoforms may preferentially play a role in its stabilization.	2.27
PITPNA	Phosphatidylinositol transfer protein alpha isoform; Catalyzes the transfer of PtdIns and phosphatidylcholine between membranes; Phosphatidylinositol transfer proteins	2.26
IGF2	Insulin-like growth factor 2 (somatomedin A); The insulin-like growth factors possess growth-promoting activity. Major fetal growth hormone in mammals. Plays a key role in regulating fetoplacental development. IGF-II is influenced by placental lactogen. Also involved in tissue differentiation.	2.25
FMR1	Fragile X mental retardation protein 1; Multifunctional polyribosome-associated RNA-binding protein that plays a central role in neuronal development and synaptic plasticity through the regulation of alternative mRNA splicing, mRNA stability, mRNA dendritic transport and postsynaptic local protein synthesis of a subset of mRNAs. Plays a role in the alternative splicing of its own mRNA. Plays a role in mRNA nuclear export (By similarity).	2.22
SMARCB1	SWI/SNF related, matrix associated, actin dependent regulator of chromatin, subfamily b, member 1; Core component of the BAF (hSWI/SNF) complex. This ATP- dependent chromatin-remodeling complex plays important roles in cell proliferation and differentiation, in cellular antiviral activities and inhibition of tumor formation. The BAF complex is able to create a stable, altered form of chromatin that constrains fewer negative supercoils than normal.	2.18
MSX1	Msh homeobox 1-like protein; Acts as a transcriptional repressor. May play a role in limb-pattern formation. Acts in cranofacial development and specifically in odontogenesis.	2.02
AXL	Tyrosine-protein kinase receptor UFO; Receptor tkinase that transduces signals from the extracellular matrix intcytoplasm by binding growth factor GAS6 and which is thus regulating many phgical processes including cell survival, cell proliferation, migration, and differentiation. Ligand binding at the cell surface induces dimerization and autophosphorylation of AXL. Following activation by ligand, ALX binds and induces tyrosine phosphorylation of PI3- kinase subunits PIK3R1, PIK3R2 and PIK3R3; but also, GRB2, PLCG1, LCK and PTPN11.	1.99
CTGF	Hypertrophic chondrocyte-specific protein 24; Major connective tissue mitoattractant secreted by vascular endothelial cells. Promotes proliferation and differentiation of chondrocytes. Mediates heparin- and divalent cation-dependent cell adhesion in many cell types including	1.99

	fibroblasts, myofibroblasts, endothelial and epithelial cells. Enhances fibroblast growth factor-induced DNA synthesis; Belongs to the CCN family.	
VAV1	Vav 1 guanine nucleotide exchange factor; Couples tyrosine kinase signals with the activation of the Rho/Rac GTPases, thus leading to cell differentiation and/or proliferation; Pleckstrin homology domain containing	1.98
PLAC8	Placenta-specific gene 8 protein; Placenta specific 8	1.95
ANGPT2	Angiopoietin 2; Binds to TEK/TIE2, competing for the ANGPT1 binding site, and modulating ANGPT1 signaling. Can induce tyrosine phosphorylation of TEK/TIE2 in the absence of ANGPT1. In the absence of angiogenic inducers, such as VEGF, ANGPT2-mediated loosening of cell-matrix contacts may induce endothelial cell apoptosis with consequent vascular regression. In concert with VEGF, it may facilitate endothelial cell migration and proliferation, thus serving as a permissive angiogenic signal.	1.93
ITGB3	Integrin, beta 3 (platelet glycoprotein IIIa, antigen CD61); Integrin alpha-V/beta-3 (ITGAV:ITGB3) is a receptor for cytotactin, fibronectin, laminin, matrix metalloproteinase-2, osteopontin, osteomodulin, prothrombin, thrombospondin, vitronectin and von Willebrand factor. Integrin alpha-IIb/beta-3 (ITGA2B:ITGB3) is a receptor for fibronectin, fibrinogen, plasminogen, prothrombin, thrombospondin and vitronectin. Integrins alpha-IIb/beta-3 and alpha-V/beta-3 recognize the sequence R-G-D in a wide array of ligands. Integrin alpha- IIb/beta-3 recognizes the sequence H-H-L-G-G-G-A-K-Q-A-G-D-V in fibrinogen gamma chain.	1.91
TNF	Tumor necrosis factor ligand superfamily member 2; Cytokine that binds to TNFRSF1A/TNFR1 and TNFRSF1B/TNFBR. It is mainly secreted by macrophages and can induce cell death of certain tumor cell lines. It is potent pyrogen causing fever by direct action or by stimulation of interleukin-1 secretion and is implicated in the induction of cachexia. Under certain conditions it can stimulate cell proliferation and induce cell differentiation.	1.89
ROS1	ROS proto-oncogene 1, receptor tyrosine kinase; Orphan receptor tyrosine kinase (RTK) that plays a role in epithelial cell differentiation and regionalization of the proximal epididymal epithelium. May activate several downstream signaling pathways related to cell differentiation, proliferation, growth, and survival including the PI3 kinase-mTOR signaling pathway. Mediates the phosphorylation of PTPN11, an activator of this pathway.	1.86
CD74	CD74 molecule, major histocompatibility complex, class II invariant chain; Plays a critical role in MHC class II antigen processing by stabilizing peptide-free class II alpha/beta heterodimers in a complex soon after their synthesis and directing transport of the complex from the endoplasmic reticulum to the endosomal/lysosomal system where the antigen processing and binding of antigenic peptides to MHC class II takes place. Serves as cell surface receptor for the cytokine MIF; CD molecules	1.86

KDM5B	Retinoblastoma-binding protein 2 homolog 1; Histone demethylase that demethylates 'Lys-4' of histone H3, thereby playing a central role in histone code. Does not demethylate histone H3 'Lys-9' or H3 'Lys-27'. Demethylates trimethylated, dimethylated and monomethylated H3 'Lys-4'. Acts as a transcriptional corepressor for FOXG1B and PAX9. Favors the proliferation of breast cancer cells by repressing tumor suppressor genes such as BRCA1 and HOXA5.	1.83
TNFRSF8	Tumor necrosis factor receptor superfamily, member 8; Receptor for TNFSF8/CD30L. May play a role in the regulation of cellular growth and transformation of activated lymphoblasts. Regulates gene expression through activation of NF- kappa-B.	1.82
ETV1	ETS translocation variant 1; Transcriptional activator that binds to DNA sequences containing the consensus pentanucleotide 5'-CGGA[AT]-3'; Belongs to the ETS family.	1.78
SKIL	SKI-like proto-oncogene; May have regulatory role in cell division or differentiation in response to extracellular signals; Belongs to the SKI family.	1.77
SEMA3C	Sema domain, immunoglobulin domain (Ig), short basic domain, secreted, (semaphorin) 3C; Binds to plexin family members and plays an important role in the regulation of developmental processes. Required for normal cardiovascular development during embryogenesis. Functions as attractant for growing axons, and thereby plays an important role in axon growth and axon guidance (By similarity); I-set domain containing	1.76
RRAS	Related RAS viral (r-ras) oncogene homolog; Regulates the organization of the actin cytoskeleton. With OSPBL3, modulates integrin beta-1 (ITGB1) activity; Belongs to the small GTPase superfamily. Ras family.	1.75
ACHE	Acetylcholinesterase (Yt blood group); Terminates signal transduction at the neuromuscular junction by rapid hydrolysis of the acetylcholine released into the synaptic cleft. Role in neuronal apoptosis; Belongs to the type-B carboxylesterase/lipase family.	1.74
DAB2	Dab, mitogen-responsive phosphoprotein, homolog 2 (Drosophila); Adapter protein that functions as clathrin-associated sorting protein (CLASP) required for clathrin-mediated endocytosis of selected cargo proteins. Can bind and assemble clathrin, and binds simultaneously to phosphatidylinositol 4,5-bisphosphate (PtdIns(4,5)P2) and cargos containing non-phosphorylated NPXY internalization motifs, such as the LDL receptor, to recruit them to clathrin-coated pits. Can function in clathrin-mediated endocytosis independently of the AP-2 complex.	1.69
FGF8	Fibroblast growth factor 8 (androgen-induced); Plays an important role in the regulation of embryonic development, cell proliferation, cell differentiation and cell migration. Required for normal brain, eye, ear, and limb development during embryogenesis. Required for normal development of the gonadotropin-releasing hormone (GnRH) neuronal system.	1.69
MED1	Thyroid hormone receptor-associated protein complex 220 kDa component; Component of the Mediator complex, a coactivator involved in the regulated transcription of nearly all RNA polymerase II-dependent genes. Mediator functions as a bridge to convey information from gene-	1.68

	specific regulatory proteins to the basal RNA polymerase II transcription machinery. Mediator is recruited to promoters by direct interactions with regulatory proteins and serves as a scaffold for the assembly of a functional preinitiation complex with RNA polymerase II and the general transcription factors. Acts as a coactivator for GATA1-mediated transcriptional activation during erythroid differentiation of K562 erythroleukemia cells.	
TYROBP	Killer-activating receptor-associated protein; Non-covalently associates with activating receptors of the CD300 family. Cross-linking of CD300-TYROBP complexes results in cellular activation. Involved for instance in neutrophil activation mediated by integrin.	1.68
RAPGEF1	Rap guanine nucleotide exchange factor (GEF) 1; Guanine nucleotide-releasing protein that binds to SH3 domain of CRK and GRB2/ASH. Transduces signals from CRK to activate RAS. Plays a role in the establishment of basal endothelial barrier function.	1.68
GAS1	Growth arrest-specific protein 1; Specific growth arrest protein involved in growth suppression. Blocks entry to S phase. Prevents cycling of normal and transformed cells.	1.65
SMAD3	Mothers against decapentaplegic homolog 3; Receptor-regulated SMAD (R-SMAD) that is an intracellular signal transducer and transcriptional modulator activated by TGF-beta (transforming growth factor) and activin type 1 receptor kinases. Binds the TRE element in the promoter region of many genes that are regulated by TGF-beta and, on formation of the SMAD3/SMAD4 complex, activates transcription. Also, can form a SMAD3/SMAD4/JUN/FOS complex at the AP-1/SMAD site to regulate TGF-beta-mediated transcription.	1.65
ABL2	ABL proto-oncogene 2, non-receptor tyrosine kinase; Non-receptor tyrosine-protein kinase that plays an ABL1- overlapping role in key processes linked to cell growth and survival such as cytoskeleton remodeling in response to extracellular stimuli, cell motility and adhesion and receptor endocytosis. Coordinates actin remodeling through tyrosine phosphorylation of proteins controlling cytoskeleton dynamics like MYH10 (involved in movement); CTTN (involved in signaling); or TUBA1 and TUBB (microtubule subunits). Binds directly F-actin and regulates actin cytoskeletal structure through its F-actin- bundling activity.	1.65
PGF	Placental growth factor; Growth factor active in angiogenesis and endothelial cell growth, stimulating their proliferation and migration. It binds to the receptor FLT1/VEGFR-1. Isoform PlGF-2 binds NRP1/neuropilin-1 and NRP2/neuropilin-2 in a heparin-dependent manner. Also promotes cell tumor growth.	1.62
CCND1	B-cell lymphoma 1 protein; Regulatory component of the cyclin D1-CDK4 (DC) complex that phosphorylates and inhibits members of the retinoblastoma (RB) protein family including RB1 and regulates the cell-cycle during G(1)/S transition. Phosphorylation of RB1 allows dissociation of the transcription factor E2F from the RB/E2F complex and the subsequent transcription of E2F target genes which are responsible for the progression through the G(1) phase. Hypophosphorylates RB1 in	1.55

	early G(1) phase. Cyclin D-CDK4 complexes are major integrators of various mitogenic and antimitogenic signals.	
ADAM17	Disintegrin and metalloproteinase domain-containing protein 17; Cleaves the membrane-bound precursor of TNF-alpha to its mature soluble form. Responsible for the proteolytical release of soluble JAM3 from endothelial cells surface. Responsible for the proteolytic release of several other cell-surface proteins, including p75 TNF-receptor, interleukin 1 receptor type II, p55 TNF-receptor, transforming growth factor-alpha, L-selectin, growth hormone receptor, MUC1 and the amyloid precursor protein.	1.54
WT1	Wilms tumor protein: Transcription factor that plays an important role in cellular development and cell survival. Recognizes and binds to the DNA sequence 5'-GCG(T/G)GGGCG-3'. Regulates the expression of numerous target genes, including EPO.	1.51
ITGA7	Integrin, alpha 7; Integrin alpha-7/beta-1 is the primary laminin receptor on skeletal myoblasts and adult myofibers. During myogenic differentiation, it may induce changes in the shape and mobility of myoblasts and facilitate their localization at laminin-rich sites of secondary fiber formation. It is involved in the maintenance of the myofibers cytoarchitecture as well as for their anchorage, viability, and functional integrity.	1.51
AXIN2	Axis inhibition protein 2; Inhibitor of the Wnt signaling pathway. Down-regulates beta-catenin. Probably facilitate the phosphorylation of beta-catenin and APC by GSK3B (By similarity).	1.50
**GO:0042127 Regulation of cell population**		
**Symbol**	**Description**	**Z-score**
FAP	170 kDa melanoma membrane-bound gelatinase; Cell surface glycoprotein serine protease that participates in extracellular matrix degradation and involved in many cellular processes including tissue remodeling, fibrosis, wound healing, inflammation, and tumor growth. Both plasma membrane and soluble forms exhibit post-proline cleaving endopeptidase activity, with a marked preference for Ala/Ser-Gly-Pro-Ser/Asn/Ala consensus sequences, on substrate such as alpha-2-antiplasmin SERPINF2 and SPRY2. Also degrade gelatin, heat-denatured type I collagen, but not native collagen type I and IV, vibronectin, tenascin, laminin, fibronectin, fibrin, or casein.	2.73
SMAD2	Mothers against decapentaplegic homolog 2; Receptor-regulated SMAD (R-SMAD) that is an intracellular signal transducer and transcriptional modulator activated by TGF-beta (transforming growth factor) and activin type 1 receptor kinases. Binds the TRE element in the promoter region of many genes that are regulated by TGF-beta and, on formation of the SMAD2/SMAD4 complex, activates transcription. May act as a tumor suppressor in colorectal carcinoma.	2.52
IGF2	Insulin-like growth factor 2 (somatomedin A); The insulin-like growth factors possess growth-promoting activity. Major fetal growth hormone in mammals. Plays a key role in regulating fetoplacental development.	2.25

	IGF-II is influenced by placental lactogen. Also involved in tissue differentiation.	
DPP4	Adenosine deaminase complexing protein 2; Cell surface glycoprotein receptor involved in the costimulatory signal essential for T-cell receptor (TCR)-mediated T-cell activation. Acts as a positive regulator of T-cell coactivation, by binding at least ADA, CAV1, IGF2R, and PTPRC. Its binding to CAV1 and CARD11 induces T-cell proliferation and NF- kappa-B activation in a T-cell receptor/CD3-dependent manner.	2.20
SMARCB1	SWI/SNF related, matrix associated, actin dependent regulator of chromatin, subfamily b, member 1; Core component of the BAF (hSWI/SNF) complex. This ATP- dependent chromatin-remodeling complex plays important roles in cell proliferation and differentiation, in cellular antiviral activities and inhibition of tumor formation. The BAF complex is able to create a stable, altered form of chromatin that constrains fewer negative supercoils than normal.	2.18
HMGA1	High mobility group protein HMG-I/HMG-Y; HMG-I/Y bind preferentially to the minor groove of A+T rich regions in double-stranded DNA. It is suggested that these proteins could function in nucleosome phasing and in the 3'-end processing of mRNA transcripts. They are also involved in the transcription regulation of genes containing, or in close proximity to A+T-rich regions; Canonical high mobility group	2.15
MSX1	Msh homeobox 1-like protein; Acts as a transcriptional repressor. May play a role in limb-pattern formation. Acts in cranofacial development and specifically in odontogenesis. Expression in the developing nail bed mesenchyme is important for nail plate thickness and integrity; Belongs to the Msh homeobox family.	2.02
CTGF	Hypertrophic chondrocyte-specific protein 24; Major connective tissue mitoattractant secreted by vascular endothelial cells. Promotes proliferation and differentiation of chondrocytes. Mediates heparin- and divalent cation-dependent cell adhesion in many cell types including fibroblasts, myofibroblasts, endothelial and epithelial cells.	1.99
PLAC8	Placenta-specific gene 8 protein; Placenta specific 8	1.95
ITGB3	Integrin, beta 3 (platelet glycoprotein IIIa, antigen CD61); Integrin alpha-V/beta-3 (ITGAV:ITGB3) is a receptor for cytotactin, fibronectin, laminin, matrix metalloproteinase-2, osteopontin, osteomodulin, prothrombin, thrombospondin, vitronectin and von Willebrand factor. Integrin alpha-IIb/beta-3 (ITGA2B:ITGB3) is a receptor for fibronectin, fibrinogen, plasminogen, prothrombin, thrombospondin and vitronectin. Integrins alpha-IIb/beta-3 and alpha-V/beta-3 recognize the sequence R-G-D in a wide array of ligands. Integrin alpha- IIb/beta-3 recognizes the sequence H-H-L-G-G-G-A-K-Q-A-G-D-V in fibrinogen gamma chain.	1.91
TNF	Tumor necrosis factor ligand superfamily member 2; Cytokine that binds to TNFRSF1A/TNFR1 and TNFRSF1B/TNFBR. It is mainly secreted by macrophages and can induce cell death of certain tumor cell lines. It is potent pyrogen causing fever by direct action or by stimulation of interleukin-1 secretion and is implicated in the induction of cachexia.	1.89

	Under certain conditions it can stimulate cell proliferation and induce cell differentiation.	
CD74	CD74 molecule, major histocompatibility complex, class II invariant chain; Plays a critical role in MHC class II antigen processing by stabilizing peptide-free class II alpha/beta heterodimers in a complex soon after their synthesis and directing transport of the complex from the endoplasmic reticulum to the endosomal/lysosomal system where the antigen processing and binding of antigenic peptides to MHC class II takes place. Serves as cell surface receptor for the cytokine MIF; CD molecules	1.86
KDM5B	Retinoblastoma-binding protein 2 homolog 1; Histone demethylase that demethylates 'Lys-4' of histone H3, thereby playing a central role in histone code. Does not demethylate histone H3 'Lys-9' or H3 'Lys-27'. Demethylates trimethylated, dimethylated and monomethylated H3 'Lys-4'. Acts as a transcriptional corepressor for FOXG1B and PAX9. Favors the proliferation of breast cancer cells by repressing tumor suppressor genes such as BRCA1 and HOXA5.	1.83
TNFRSF8	Tumor necrosis factor receptor superfamily, member 8; Receptor for TNFSF8/CD30L. May play a role in the regulation of cellular growth and transformation of activated lymphoblasts. Regulates gene expression through activation of NF- kappa-B.	1.82
ASPH	Aspartyl/asparaginyl beta-hydroxylase; Isoform 1: specifically hydroxylates an Asp or Asn residue in certain epidermal growth factor-like (EGF) domains of a number of proteins; Belongs to the aspartyl/asparaginyl beta-hydroxylase family.	1.76
APC	Adenomatous polyposis coli protein; Tumor suppressor. Promotes rapid degradation of CTNNB1 and participates in Wnt signaling as a negative regulator. APC activity is correlated with its phosphorylation state.	1.75
IGFBP7	Insulin-like growth factor binding protein 7; Binds IGF-I and IGF-II with a relatively low affinity. Stimulates prostacyclin (PGI2) production. Stimulates cell adhesion; I-set domain containing	1.75
CDH13	Truncated cadherin; Cadherins are calcium-dependent cell adhesion proteins. They preferentially interact with themselves in a homophilic manner in connecting cells; cadherins may thus contribute to the sorting of heterogeneous cell types. May act as a negative regulator of neural cell growth.	1.72
VIPR1	Pituitary adenylate cyclase-activating polypeptide type II receptor; This is a receptor for VIP. The activity of this receptor is mediated by G proteins which activate adenylyl cyclase. The affinity is VIP = PACAP-27 > PACAP-38; Vasoactive intestinal peptide receptor family	1.71
FGF8	Fibroblast growth factor 8 (androgen-induced); Plays an important role in the regulation of embryonic development, cell proliferation, cell differentiation and cell migration. Required for normal brain, eye, ear, and limb development during embryogenesis. Required for normal development of the gonadotropin-releasing hormone (GnRH) neuronal system.	1.69
MED1	Thyroid hormone receptor-associated protein complex 220 kDa component; Component of the Mediator complex, a coactivator involved	1.68

	in the regulated transcription of nearly all RNA polymerase II-dependent genes. Mediator functions as a bridge to convey information from gene-specific regulatory proteins to the basal RNA polymerase II transcription machinery. Mediator is recruited to promoters by direct interactions with regulatory proteins and serves as a scaffold for the assembly of a functional preinitiation complex with RNA polymerase II and the general transcription factors. Acts as a coactivator for GATA1-mediated transcriptional activation during erythroid differentiation of K562 erythroleukemia cells.	
RAPGEF1	Rap guanine nucleotide exchange factor (GEF) 1; Guanine nucleotide-releasing protein that binds to SH3 domain of CRK and GRB2/ASH. Transduces signals from CRK to activate RAS. Plays a role in the establishment of basal endothelial barrier function. Plays a role in nerve growth factor (NGF)-induced sustained activation of Rap1 and neurite outgrowth.	1.68
FLT4	Vascular endothelial growth factor receptor 3; Tyrosine-protein kinase that acts as a cell-surface receptor for VEGFC and VEGFD and plays an essential role in adult lymphangiogenesis and in the development of the vascular network and the cardiovascular system during embryonic development. Promotes proliferation, survival, and migration of endothelial cells, and regulates angiogenic sprouting. Signaling by activated FLT4 leads to enhanced production of VEGFC, and to a lesser degree VEGFA, thereby creating a positive feedback loop that enhances FLT4 signaling. Modulates KDR signaling by forming heterodimers.	1.66
SMAD3	Mothers against decapentaplegic homolog 3; Receptor-regulated SMAD (R-SMAD) that is an intracellular signal transducer and transcriptional modulator activated by TGF-beta (transforming growth factor) and activin type 1 receptor kinases. Binds the TRE element in the promoter region of many genes that are regulated by TGF-beta and, on formation of the SMAD3/SMAD4 complex, activates transcription. Also, can form a SMAD3/SMAD4/JUN/FOS complex at the AP-1/SMAD site to regulate TGF-beta-mediated transcription.	1.65
ABL2	ABL proto-oncogene 2, non-receptor tyrosine kinase; Non-receptor tyrosine-protein kinase that plays an ABL1- overlapping role in key processes linked to cell growth and survival such as cytoskeleton remodeling in response to extracellular stimuli, cell motility and adhesion and receptor endocytosis. Coordinates actin remodeling through tyrosine phosphorylation of proteins controlling cytoskeleton dynamics like MYH10 (involved in movement); CTTN (involved in signaling); or TUBA1 and TUBB (microtubule subunits). Binds directly F-actin and regulates actin cytoskeletal structure through its F-actin- bundling activity.	1.65
PGF	Placental growth factor; Growth factor active in angiogenesis and endothelial cell growth, stimulating their proliferation and migration. It binds to the receptor FLT1/VEGFR-1. Isoform PlGF-2 binds NRP1/neuropilin-1 and NRP2/neuropilin-2 in a heparin-dependent manner. Also promotes cell tumor growth.	1.62

TNFRSF4	TAX transcriptionally-activated glycoprotein 1 receptor; Receptor for TNFSF4/OX40L/GP34. Is a costimulatory molecule implicated in long-term T-cell immunity; CD molecules	1.61
TINF2	TERF1 (TRF1)-interacting nuclear factor 2; Component of the shelterin complex (telosome) that is involved in the regulation of telomere length and protection. Shelterin associates with arrays of double-stranded TTAGGG repeats added by telomerase and protects chromosome ends; without its protective activity, telomeres are no longer hidden from the DNA damage surveillance and chromosome ends are inappropriately processed by DNA repair pathways. Plays a role in shelterin complex assembly. Isoform 1 may have additional role in tethering telomeres to the nuclear matrix.	1.58
ADAMTS8	A disintegrin and metalloproteinase with thrombospondin motifs 8; Has anti-angiogenic properties; ADAM metallopeptidases with thrombospondin type 1 motif	1.56
CCND1	B-cell lymphoma 1 protein; Regulatory component of the cyclin D1-CDK4 (DC) complex that phosphorylates and inhibits members of the retinoblastoma (RB) protein family including RB1 and regulates the cell-cycle during G(1)/S transition. Phosphorylation of RB1 allows dissociation of the transcription factor E2F from the RB/E2F complex and the subsequent transcription of E2F target genes which are responsible for the progression through the G(1) phase. Hypophosphorylates RB1 in early G(1) phase. Cyclin D-CDK4 complexes are major integrators of various mitogenenic and antimitogenic signals.	1.55
ADAM17	Disintegrin and metalloproteinase domain-containing protein 17; Cleaves the membrane-bound precursor of TNF-alpha to its mature soluble form. Responsible for the proteolytical release of soluble JAM3 from endothelial cells surface. Responsible for the proteolytic release of several other cell-surface proteins, including p75 TNF-receptor, interleukin 1 receptor type II, p55 TNF-receptor, transforming growth factor-alpha, L-selectin, growth hormone receptor, MUC1 and the amyloid precursor protein.	1.54
WT1	Wilms tumor protein: Transcription factor that plays an important role in cellular development and cell survival. Recognizes and binds to the DNA sequence 5'-GCG(T/G)GGGCG-3'. Regulates the expression of numerous target genes, including EPO.	1.51
AXIN2	Axis inhibition protein 2; Inhibitor of the Wnt signaling pathway. Down-regulates beta-catenin. Probably facilitate the phosphorylation of beta-catenin and APC by GSK3B (By similarity).	1.50
**GO:0016477 Cell migration**		
**Symbol**	**Description**	**Z-score**
FAP	170 kDa melanoma membrane-bound gelatinase; Cell surface glycoprotein serine protease that participates in extracellular matrix degradation and involved in many cellular processes including tissue remodeling, fibrosis, wound healing, inflammation, and tumor growth. Both plasma membrane and soluble forms exhibit post-proline cleaving endopeptidase activity, with a marked preference for Ala/Ser-Gly-Pro-	2.73

	Ser/Asn/Ala consensus sequences, on substrate such as alpha-2-antiplasmin SERPINF2 and SPRY2. Also degrade gelatin, heat-denatured type I collagen, but not native collagen type I and IV, vibronectin, tenascin, laminin, fibronectin, fibrin, or casein.	
RPS19	Small ribosomal subunit protein eS19; Required for pre-rRNA processing and maturation of 40S ribosomal subunits; Belongs to the eukaryotic ribosomal protein eS19 family.	2.63
L1CAM	Neural cell adhesion molecule L1; Neural cell adhesion molecule involved in the dynamics of cell adhesion and in the generation of transmembrane signals at tyrosine kinase receptors. During brain development, critical in multiple processes, including neuronal migration, axonal growth and fasciculation, and synaptogenesis. In the mature brain, plays a role in the dynamics of neuronal structure and function, including synaptic plasticity; CD molecules	2.30
DPP4	Adenosine deaminase complexing protein 2; Cell surface glycoprotein receptor involved in the costimulatory signal essential for T-cell receptor (TCR)-mediated T-cell activation. Acts as a positive regulator of T-cell coactivation, by binding at least ADA, CAV1, IGF2R, and PTPRC. Its binding to CAV1 and CARD11 induces T-cell proliferation and NF- kappa-B activation in a T-cell receptor/CD3-dependent manner.	2.20
AXL	Tyrosine-protein kinase receptor UFO; Receptor tkinase that transduces signals from the extracellular matrix intcytoplasm by binding growth factor GAS6 and which is thus regulating many phgical processes including cell survival, cell proliferation, migration, and differentiation. Ligand binding at the cell surface induces dimerization and autophosphorylation of AXL. Following activation by ligand, ALX binds and induces tyrosine phosphorylation of PI3- kinase subunits PIK3R1, PIK3R2 and PIK3R3; but also, GRB2, PLCG1, LCK and PTPN11.	1.99
CTGF	Hypertrophic chondrocyte-specific protein 24; Major connective tissue mitoattractant secreted by vascular endothelial cells. Promotes proliferation and differentiation of chondrocytes. Mediates heparin- and divalent cation-dependent cell adhesion in many cell types including fibroblasts, myofibroblasts, endothelial and epithelial cells.	1.99
BIN2	Breast cancer-associated protein 1; Promotes cell motility and migration, probably via its interaction with the cell membrane and with podosome proteins that mediate interaction with the cytoskeleton. Modulates membrane curvature and mediates membrane tubulation. Plays a role in podosome formation. Inhibits phagocytosis; N-BAR domain containing	1.99
VAV1	Vav 1 guanine nucleotide exchange factor; Couples tyrosine kinase signals with the activation of the Rho/Rac GTPases, thus leading to cell differentiation and/or proliferation; Pleckstrin homology domain containing	1.98
ANGPT2	Angiopoietin 2; Binds to TEK/TIE2, competing for the ANGPT1 binding site, and modulating ANGPT1 signaling. Can induce tyrosine phosphorylation of TEK/TIE2 in the absence of ANGPT1. In the absence of angiogenic inducers, such as VEGF, ANGPT2-mediated loosening of cell-matrix contacts may induce endothelial cell apoptosis with consequent vascular regression. In concert with VEGF, it may facilitate	1.93

	endothelial cell migration and proliferation, thus serving as a permissive angiogenic signal.	
ITGB3	Integrin, beta 3 (platelet glycoprotein IIIa, antigen CD61); Integrin alpha-V/beta-3 (ITGAV:ITGB3) is a receptor for cytotactin, fibronectin, laminin, matrix metalloproteinase-2, osteopontin, osteomodulin, prothrombin, thrombospondin, vitronectin and von Willebrand factor. Integrin alpha-IIb/beta-3 (ITGA2B:ITGB3) is a receptor for fibronectin, fibrinogen, plasminogen, prothrombin, thrombospondin and vitronectin. Integrins alpha-IIb/beta-3 and alpha-V/beta-3 recognize the sequence R-G-D in a wide array of ligands. Integrin alpha- IIb/beta-3 recognizes the sequence H-H-L-G-G-G-A-K-Q-A-G-D-V in fibrinogen gamma chain.	1.91
TNF	Tumor necrosis factor ligand superfamily member 2; Cytokine that binds to TNFRSF1A/TNFR1 and TNFRSF1B/TNFBR. It is mainly secreted by macrophages and can induce cell death of certain tumor cell lines. It is potent pyrogen causing fever by direct action or by stimulation of interleukin-1 secretion and is implicated in the induction of cachexia. Under certain conditions it can stimulate cell proliferation and induce cell differentiation.	1.89
CD74	CD74 molecule, major histocompatibility complex, class II invariant chain; Plays a critical role in MHC class II antigen processing by stabilizing peptide-free class II alpha/beta heterodimers in a complex soon after their synthesis and directing transport of the complex from the endoplasmic reticulum to the endosomal/lysosomal system where the antigen processing and binding of antigenic peptides to MHC class II takes place. Serves as cell surface receptor for the cytokine MIF; CD molecules	1.86
SEMA3C	Sema domain, immunoglobulin domain (Ig), short basic domain, secreted, (semaphorin) 3C; Binds to plexin family members and plays an important role in the regulation of developmental processes. Required for normal cardiovascular development during embryogenesis. Functions as attractant for growing axons, and thereby plays an important role in axon growth and axon guidance (By similarity); I-set domain containing	1.76
VAV2	Vav 2 guanine nucleotide exchange factor; Guanine nucleotide exchange factor for the Rho family of Ras-related GTPases. Plays an important role in angiogenesis. Its recruitment by phosphorylated EPHA2 is critical for EFNA1-induced RAC1 GTPase activation and vascular endothelial cell migration and assembly (By similarity); Pleckstrin homology domain containing	1.76
APC	Adenomatous polyposis coli protein; Tumor suppressor. Promotes rapid degradation of CTNNB1 and participates in Wnt signaling as a negative regulator. APC activity is correlated with its phosphorylation state.	1.75
CDH13	Truncated cadherin; Cadherins are calcium-dependent cell adhesion proteins. They preferentially interact with themselves in a homophilic manner in connecting cells; cadherins may thus contribute to the sorting of heterogeneous cell types. May act as a negative regulator of neural cell growth.	1.72
FGF8	Fibroblast growth factor 8 (androgen-induced); Plays an important role in the regulation of embryonic development, cell proliferation, cell differentiation and cell migration. Required for normal brain, eye, ear,	1.69

	and limb development during embryogenesis. Required for normal development of the gonadotropin-releasing hormone (GnRH) neuronal system.	
EPX	Eosinophil peroxidase; Mediates tyrosine nitration of secondary granule proteins in mature resting eosinophils. Shows significant inhibitory activity towards Mycobacterium tuberculosis H37Rv by inducing bacterial fragmentation and lysis; Belongs to the peroxidase family.	1.62
ADAM17	Disintegrin and metalloproteinase domain-containing protein 17; Cleaves the membrane-bound precursor of TNF-alpha to its mature soluble form. Responsible for the proteolytical release of soluble JAM3 from endothelial cells surface. Responsible for the proteolytic release of several other cell-surface proteins, including p75 TNF-receptor, interleukin 1 receptor type II, p55 TNF-receptor, transforming growth factor-alpha, L-selectin, growth hormone receptor, MUC1 and the amyloid precursor protein.	1.54
**Down-Regulated Transcripts after 48 h of UTP**		
**GO:0042493 Response to drug**		
**Symbol**	**Description**	**Z-score**
FZD4	Frizzled class receptor 4; Receptor for Wnt proteins. Most of frizzled receptors are coupled to the beta-catenin (CTNNB1) canonical signaling pathway, which leads to the activation of disheveled proteins, inhibition of GSK-3 kinase, nuclear accumulation of beta-catenin (CTNNB1) and activation of Wnt target genes. Plays a critical role in retinal vascularization by acting as a receptor for Wnt proteins and norrin (NDP).	-3.21
CYP2C19	Cytochrome P450, family 2, subfamily C, polypeptide 19; Responsible for the metabolism of a number of therapeutic agents such as the anticonvulsant drug S-mephenytoin, omeprazole, proguanil, certain barbiturates, diazepam, propranolol, citalopram, and imipramine; Belongs to the cytochrome P450 family.	-2.38
CREB1	Cyclic AMP-responsive element-binding protein 1; Phosphorylation-dependent transcription factor that stimulates transcription upon binding to the DNA cAMP response element (CRE), a sequence present in many viral and cellular promoters. Transcription activation is enhanced by the TORC coactivators which act independently of Ser-133 phosphorylation. Involved in different cellular processes including the synchronization of circadian rhythmicity and the differentiation of adipose cells; Basic leucine zipper proteins	-2.30
ANXA1	Phospholipase A2 inhibitory protein; Plays important roles in the innate immune response as effector of glucocorticoid-mediated responses and regulator of the inflammatory process. Has anti-inflammatory activity. Plays a role in glucocorticoid-mediated down- regulation of the early phase of the inflammatory response (By similarity).	-2.02
NR4A3	Nuclear receptor subfamily 4, group A, member 3; Transcriptional activator that binds to regulatory elements in promoter regions in a cell- and response element (target)-specific manner. Induces gene expression by binding as monomers to the NR4A1 response element (NBRE) 5'-AAAAGGTCA-3' site and as homodimers to the Nur response element	-1.88

	(NurRE) site in the promoter of their regulated target genes (By similarity). Plays a role in the regulation of proliferation, survival, and differentiation of many different cell types and also in metabolism and inflammation.	
CSF3	Colony stimulating factor 3 (granulocyte); Granulocyte/macrophage colony-stimulating factors are cytokines that act in hematopoiesis by controlling the production, differentiation, and function of 2 related white cell populations of the blood, the granulocytes, and the monocytes-macrophages. This CSF induces granulocytes; Belongs to the IL-6 superfamily.	-1.82
ABL1	ABL proto-oncogene 1, non-receptor tyrosine kinase; Non-receptor tyrosine-protein kinase that plays a role in many key processes linked to cell growth and survival such as cytoskeleton remodeling in response to extracellular stimuli, cell motility and adhesion, receptor endocytosis, autophagy, DNA damage response and apoptosis. Coordinates actin remodeling through tyrosine phosphorylation of proteins controlling cytoskeleton dynamics like WASF3 (involved in branch formation); ANXA1 (involved in membrane anchoring); DBN1, DBNL, CTTN, RAPH1 and ENAH (involved in signaling); or MAPT and PXN (microtubule-binding proteins). Phosphorylation of WASF3 is critical for the stimulation of lamellipodia formation and cell migration. Involved in the regulation of cell adhesion and motility through phosphorylation of key regulators of these processes such as BCAR1, CRK, CRKL, DOK1, EFS or NEDD9.	-1.66
IL1B	Interleukin 1, beta; Potent proinflammatory cytokine. Initially discovered as the major endogenous pyrogen, induces prostaglandin synthesis, neutrophil influx and activation, T-cell activation and cytokine production, B-cell activation and antibody production, and fibroblast proliferation and collagen production. Promotes Th17 differentiation of T-cells.	-1.62
XRCC5	ATP-dependent DNA helicase II 80 kDa subunit; Single-stranded DNA-dependent ATP-dependent helicase. Has a role in chromosome translocation. The DNA helicase II complex binds preferentially to fork-like ends of double-stranded DNA in a cell cycle-dependent manner.	-1.60
CASP8	Caspase 8, apoptosis-related cysteine peptidase; Most upstream protease of the activation cascade of caspases responsible for the TNFRSF6/FAS mediated and TNFRSF1A induced cell death. Binding to the adapter molecule FADD recruits it to either receptor. The resulting aggregate called death- inducing signaling complex (DISC) performs CASP8 proteolytic activation.	-1.58
NFKBIA	Nuclear factor of kappa light polypeptide gene enhancer in B-cells inhibitor, alpha; Inhibits the activity of dimeric NF-kappa-B/REL complexes by trapping REL dimers in the cytoplasm through masking of their nuclear localization signals. On cellular stimulation by immune and proinflammatory responses, becomes phosphorylated promoting ubiquitination and degradation, enabling the dimeric RELA to translocate to the nucleus and activate transcription.	-1.58
LYN	V-yes-1 Yamaguchi sarcoma viral related oncogene homolog; Non-receptor tyrosine-protein kinase that transmits signals from cell surface	-1.58

	receptors and plays an important role in the regulation of innate and adaptive immune responses, hematopoiesis, responses to growth factors and cytokines, integrin signaling, but also responses to DNA damage and genotoxic agents	
FADD	FAS-associating death domain-containing protein; Apoptotic adaptor molecule that recruits caspase-8 or caspase-10 to the activated Fas (CD95) or TNFR-1 receptors. The resulting aggregate called the death-inducing signaling complex (DISC) performs caspase-8 proteolytic activation. Active caspase-8 initiates the subsequent cascade of caspases mediating apoptosis.	-1.56
FOS	FBJ murine osteosarcoma viral oncogene homolog; Nuclear phosphoprotein which forms a tight but non- covalently linked complex with the JUN/AP-1 transcription factor. In the heterodimer, FOS and JUN/AP-1 basic regions seem to interact with symmetrical DNA half sites. On TGF-beta activation, forms a multimeric SMAD3/SMAD4/JUN/FOS complex at the AP1/SMAD- binding site to regulate TGF-beta-mediated signaling.	-1.53
COMT	Catechol O-methyltransferase; Catalyzes the O-methylation, and thereby the inactivation, of catecholamine neurotransmitters and catechol hormones. Also shortens the biological half-lives of certain neuroactive drugs, like L-DOPA, alpha-methyl DOPA and isoproterenol; Seven-beta-strand methyltransferase motif containing	-1.51
**GO:0012501 Programmed cell death**		
**Symbol**	**Description**	**Z-score**
TRAF4	Cysteine-rich domain associated with RING and Traf domains protein 1; Adapter protein and signal transducer that links members of the tumor necrosis factor receptor (TNFR) family to different signaling pathways. Plays a role in the activation of NF-kappa-B and JNK, and in the regulation of cell survival and apoptosis. Regulates activation of NF-kappa-B in response to signaling through Toll-like receptors.	-3.50
SEPT4	Apoptosis-related protein in the TGF-beta signaling pathway; Filament-forming cytoskeletal GTPase (By similarity). May play a role in cytokinesis (Potential). Forms a filamentous structure with SEPT12, SEPT6, SEPT2 and probably SEPT4 at the sperm annulus which is required for the structural integrity and motility of the sperm tail during postmeiotic differentiation.	-2.44
DAP3	Mitochondrial small ribosomal subunit protein mS29; Involved in mediating interferon-gamma-induced cell death; Belongs to the mitochondrion-specific ribosomal protein mS29 family.	-2.30
CTSV	Cathepsin L2; Cysteine protease. May have an important role in corneal physiology; Belongs to the peptidase C1 family.	-2.27
THRB	Nuclear receptor subfamily 1 group A member 2; Nuclear hormone receptor that can act as a repressor or activator of transcription. High affinity receptor for thyroid hormones, including triiodothyronine and thyroxine; Belongs to the nuclear hormone receptor family. NR1 subfamily.	-1.98
CASP10	FAS-associated death domain protein interleukin-1B-converting enzyme 2; Involved in the activation cascade of caspases responsible for	-1.90

	apoptosis execution. Recruited to both Fas- and TNFR-1 receptors in a FADD dependent manner. May participate in the granzyme B apoptotic pathways. Cleaves and activates caspase- 3, -4, -6, -7, -8, and -9.	
TNFRSF14	Tumor necrosis factor receptor superfamily, member 14; Receptor for BTLA. Receptor for TNFSF14/LIGHT and homotrimeric TNFSF1/lymphotoxin-alpha. Involved in lymphocyte activation. Plays an important role in HSV pathogenesis because it enhanced the entry of several wild-type HSV strains of both serotypes into CHO cells, and mediated HSV entry into activated human T-cells; CD molecules	-1.73
TNFSF15	Tumor necrosis factor (ligand) superfamily, member 15; Receptor for TNFRSF25 and TNFRSF6B. Mediates activation of NF-kappa-B. Inhibits vascular endothelial growth and angiogenesis (in vitro). Promotes activation of caspases and apoptosis; Belongs to the tumor necrosis factor family.	-1.70
ABL1	ABL proto-oncogene 1, non-receptor tyrosine kinase; Non-receptor tyrosine-protein kinase that plays a role in many key processes linked to cell growth and survival such as cytoskeleton remodeling in response to extracellular stimuli, cell motility and adhesion, receptor endocytosis, autophagy, DNA damage response and apoptosis. Coordinates actin remodeling through tyrosine phosphorylation of proteins controlling cytoskeleton dynamics like WASF3 (involved in branch formation); ANXA1 (involved in membrane anchoring); DBN1, DBNL, CTTN, RAPH1 and ENAH (involved in signaling); or MAPT and PXN (microtubule-binding proteins).	-1.66
IL1B	Interleukin 1, beta; Potent proinflammatory cytokine. Initially discovered as the major endogenous pyrogen, induces prostaglandin synthesis, neutrophil influx and activation, T-cell activation and cytokine production, B-cell activation and antibody production, and fibroblast proliferation and collagen production. Promotes Th17 differentiation of T-cells.	-1.62
CASP8	Caspase 8, apoptosis-related cysteine peptidase; Most upstream protease of the activation cascade of caspases responsible for the TNFRSF6/FAS mediated and TNFRSF1A induced cell death. Binding to the adapter molecule FADD recruits it to either receptor. The resulting aggregate called death- inducing signaling complex (DISC) performs CASP8 proteolytic activation.	-1.58
NFKBIA	Nuclear factor of kappa light polypeptide gene enhancer in B-cells inhibitor, alpha; Inhibits the activity of dimeric NF-kappa-B/REL complexes by trapping REL dimers in the cytoplasm through masking of their nuclear localization signals. On cellular stimulation by immune and proinflammatory responses, becomes phosphorylated promoting ubiquitination and degradation, enabling the dimeric RELA to translocate to the nucleus and activate transcription.	-1.58
FADD	FAS-associating death domain-containing protein; Apoptotic adaptor molecule that recruits caspase-8 or caspase-10 to the activated Fas (CD95) or TNFR-1 receptors. The resulting aggregate called the death-inducing signaling complex (DISC) performs caspase-8 proteolytic	-1.56

	activation. Active caspase-8 initiates the subsequent cascade of caspases mediating apoptosis.	
CD70	Tumor necrosis factor ligand superfamily member 7; Cytokine that binds to CD27. Plays a role in T-cell activation. Induces the proliferation of co-stimulated T-cells and enhances the generation of cytolytic T-cells; Belongs to the tumor necrosis factor family.	-1.53
HIC1	Zinc finger and BTB domain-containing protein 29; Transcriptional repressor. Recognizes and binds to the consensus sequence '5-[CG]NG[CG]GGGCA[CA]CC-3'. May act as a tumor suppressor.	-1.52
**GO:0045785 Positive regulation of cell adhesion**		
**Symbol**	**Description**	**Z-score**
TEK	Tyrosine kinase with Ig and EGF homology domains-2; Tyrosine-protein kinase that acts as cell-surface receptor for ANGPT1, ANGPT2 and ANGPT4 and regulates angiogenesis, endothelial cell survival, proliferation, migration, adhesion and cell spreading, reorganization of the actin cytoskeleton, but also maintenance of vascular quiescence. Has anti-inflammatory effects by preventing the leakage of proinflammatory plasma proteins and leukocytes from blood vessels. Required for normal angiogenesis and heart development during embryogenesis. Required for post- natal hematopoiesis.	-2.14
YES1	YES proto-oncogene 1, Src family tyrosine kinase; Non-receptor protein tyrosine kinase that is involved in the regulation of cell growth and survival, apoptosis, cell-cell adhesion, cytoskeleton remodeling, and differentiation. Stimulation by receptor tyrosine kinases (RTKs) including EGRF, PDGFR, CSF1R and FGFR leads to recruitment of YES1 to the phosphorylated receptor, and activation and phosphorylation of downstream substrates. Upon EGFR activation, promotes the phosphorylation of PARD3 to favor epithelial tight junction assembly.	-2.02
ANXA1	Phospholipase A2 inhibitory protein; Plays important roles in the innate immune response as effector of glucocorticoid-mediated responses and regulator of the inflammatory process. Has anti-inflammatory activity. Plays a role in glucocorticoid-mediated down- regulation of the early phase of the inflammatory response (By similarity).	-2.02
NR4A3	Nuclear receptor subfamily 4, group A, member 3; Transcriptional activator that binds to regulatory elements in promoter regions in a cell- and response element (target)-specific manner. Induces gene expression by binding as monomers to the NR4A1 response element (NBRE) 5'-AAAAGGTCA-3' site and as homodimers to the Nur response element (NurRE) site in the promoter of their regulated target genes (By similarity). Plays a role in the regulation of proliferation, survival, and differentiation of many different cell types and also in metabolism and inflammation.	-1.88
CXCL12	Pre-B cell growth-stimulating factor; Chemoattractant active on T-lymphocytes, monocytes, but not neutrophils. Activates the C-X-C chemokine receptor CXCR4 to induce a rapid and transient rise in the level of intracellular calcium ions and chemotaxis. Also binds to atypical chemokine receptor ACKR3, which activates the beta-arrestin pathway	-1.87

	and acts as a scavenger receptor for SDF-1. SDF-1-beta(3-72) and SDF-1-alpha(3-67) show a reduced chemotactic activity.	
TNFRSF14	Tumor necrosis factor receptor superfamily, member 14; Receptor for BTLA. Receptor for TNFSF14/LIGHT and homotrimeric TNFSF1/lymphotoxin-alpha. Involved in lymphocyte activation. Plays an important role in HSV pathogenesis because it enhanced the entry of several wild-type HSV strains of both serotypes into CHO cells, and mediated HSV entry into activated human T-cells; CD molecules	-1.73
ABL1	ABL proto-oncogene 1, non-receptor tyrosine kinase; Non-receptor tyrosine-protein kinase that plays a role in many key processes linked to cell growth and survival such as cytoskeleton remodeling in response to extracellular stimuli, cell motility and adhesion, receptor endocytosis, autophagy, DNA damage response and apoptosis. Coordinates actin remodeling through tyrosine phosphorylation of proteins controlling cytoskeleton dynamics like WASF3 (involved in branch formation); ANXA1 (involved in membrane anchoring); DBN1, DBNL, CTTN, RAPH1 and ENAH (involved in signaling); or MAPT and PXN (microtubule-binding proteins).	-1.66
IL1B	Interleukin 1, beta; Potent proinflammatory cytokine. Initially discovered as the major endogenous pyrogen, induces prostaglandin synthesis, neutrophil influx and activation, T-cell activation and cytokine production, B-cell activation and antibody production, and fibroblast proliferation and collagen production. Promotes Th17 differentiation of T-cells.	-1.62
LYN	V-yes-1 Yamaguchi sarcoma viral related oncogene homolog; Non-receptor tyrosine-protein kinase that transmits signals from cell surface receptors and plays an important role in the regulation of innate and adaptive immune responses, hematopoiesis, responses to growth factors and cytokines, integrin signaling, but also responses to DNA damage and genotoxic agents.	-1.58
FADD	FAS-associating death domain-containing protein; Apoptotic adaptor molecule that recruits caspase-8 or caspase-10 to the activated Fas (CD95) or TNFR-1 receptors. The resulting aggregate called the death-inducing signaling complex (DISC) performs caspase-8 proteolytic activation. Active caspase-8 initiates the subsequent cascade of caspases mediating apoptosis.	-1.56
CD80	T-lymphocyte activation antigen CD80; Involved in the costimulatory signal essential for T- lymphocyte activation. T-cell proliferation and cytokine production is induced by the binding of CD28, binding to CTLA-4 has opposite effects and inhibits T-cell activation; C2-set domain containing	-1.51

Supplementary Table 3. Transcript changes in SHARED network after 24 and 48 h of UTP-treatment in AGS cells.

Up-regulated transcripts in SHARED network			
**GO: 0030154 Cell differentiation**			
**Symbol**	**Description**	**Z-score**	
		24 h	48 h
ETV7	ETS translocation variant 7; Transcriptional repressor; binds to the DNA sequence 5'- CCGGAAGT-3'. Isoform A does not seem to have a repressor activity. Isoform C does not seem to have a repressor activity; Belongs to the ETS family.	1.81	3.05
SMAD2	Mothers against decapentaplegic homolog 2; Receptor-regulated SMAD (R-SMAD) that is an intracellular signal transducer and transcriptional modulator activated by TGF-beta (transforming growth factor) and activin type 1 receptor kinases. Binds the TRE element in the promoter region of many genes that are regulated by TGF-beta and, on formation of the SMAD2/SMAD4 complex, activates transcription. May act as a tumor suppressor in colorectal carcinoma.	1.57	2.52
CAMK2A	Calcium/calmodulin-dependent protein kinase type II subunit alpha; CaM-kinase II (CAMK2) is a prominent kinase in the central nervous system that may function in long-term potentiation and neurotransmitter release. Member of the NMDAR signaling complex in excitatory synapses it may regulate NMDAR-dependent potentiation of the AMPAR and synaptic plasticity (By similarity). Phosphorylates transcription factor FOXO3 on 'Ser-298'. Activates FOXO3 transcriptional activity (By similarity); Belongs to the protein kinase superfamily.	2.10	2.30
IGF2	Insulin-like growth factor 2 (somatomedin A); The insulin-like growth factors possess growth-promoting activity. Major fetal growth hormone in mammals. Plays a key role in regulating fetoplacental development. IGF-II is influenced by placental lactogen. Also involved in tissue differentiation.	2.16	2.25
SMARCB1	SWI/SNF related, matrix associated, actin dependent regulator of chromatin, subfamily b, member 1; Core component of the BAF (hSWI/SNF) complex. This ATP- dependent chromatin-remodeling complex plays important roles in cell proliferation and differentiation, in cellular antiviral activities and inhibition of tumor formation. The BAF complex is able to create a stable, altered form of chromatin that constrains fewer negative supercoils than normal.	1.91	2.18
AXL	Tyrosine-protein kinase receptor UFO; Receptor tyrosine kinase that transduces signals from the extracellular matrix into the cytoplasm by binding growth factor GAS6 and which is thus regulating many physiological processes including cell survival, cell proliferation, migration, and differentiation. Ligand binding at the cell surface induces dimerization and autophosphorylation of AXL. Following activation by ligand, ALX binds and induces tyrosine phosphorylation of PI3- kinase subunits PIK3R1, PIK3R2 and PIK3R3; but also, GRB2, PLCG1, LCK and PTPN11.	2.64	1.99

PLAC8	Placenta-specific gene 8 protein; Placenta specific 8	2.30	1.95
ANGPT2	Angiopoietin 2; Binds to TEK/TIE2, competing for the ANGPT1 binding site, and modulating ANGPT1 signaling. Can induce tyrosine phosphorylation of TEK/TIE2 in the absence of ANGPT1. In the absence of angiogenic inducers, such as VEGF, ANGPT2-mediated loosening of cell-matrix contacts may induce endothelial cell apoptosis with consequent vascular regression	1.86	1.93
ROS1	ROS proto-oncogene 1, receptor tyrosine kinase; Orphan receptor tyrosine kinase (RTK) that plays a role in epithelial cell differentiation and regionalization of the proximal epididymal epithelium. May activate several downstream signaling pathways related to cell differentiation, proliferation, growth, and survival including the PI3 kinase-mTOR signaling pathway. Mediates the phosphorylation of PTPN11, an activator of this pathway. May also phosphorylate and activate the transcription factor STAT3 to control anchorage-independent cell growth.	1.87	1.86
CD74	CD74 molecule, major histocompatibility complex, class II invariant chain; Plays a critical role in MHC class II antigen processing by stabilizing peptide-free class II alpha/beta heterodimers in a complex soon after their synthesis and directing transport of the complex from the endoplasmic reticulum to the endosomal/lysosomal system where the antigen processing and binding of antigenic peptides to MHC class II takes place. Serves as cell surface receptor for the cytokine MIF; CD molecules	3.00	1.86
KDM5B	Retinoblastoma-binding protein 2 homolog 1; Histone demethylase that demethylates 'Lys-4' of histone H3, thereby playing a central role in histone code. Does not demethylate histone H3 'Lys-9' or H3 'Lys-27'. Demethylates trimethylated, dimethylated and monomethylated H3 'Lys-4'.	1.87	1.83
FGF8	Fibroblast growth factor 8 (androgen-induced); Plays an important role in the regulation of embryonic development, cell proliferation, cell differentiation and cell migration. Required for normal brain, eye, ear, and limb development during embryogenesis. Required for normal development of the gonadotropin-releasing hormone (GnRH) neuronal system.	1.82	1.69
MED1	Thyroid hormone receptor-associated protein complex 220 kDa component; Component of the Mediator complex, a coactivator involved in the regulated transcription of nearly all RNA polymerase II-dependent genes. Mediator functions as a bridge to convey information from gene-specific regulatory proteins to the basal RNA polymerase II transcription machinery. Mediator is recruited to promoters by direct interactions with regulatory proteins and serves as a scaffold for the assembly of a functional preinitiation complex with RNA polymerase II and the general transcription factors. Acts as a coactivator for GATA1-mediated transcriptional activation during erythroid differentiation of K562 erythroleukemia cells.	1.96	1.68
PGF	Placental growth factor; Growth factor active in angiogenesis and endothelial cell growth, stimulating their proliferation and migration. It	1.97	1.62

	binds to the receptor FLT1/VEGFR-1. Isoform PlGF-2 binds NRP1/neuropilin-1 and NRP2/neuropilin-2 in a heparin-dependent manner. Also promotes cell tumor growth.		
CCND1	B B-cell lymphoma 1 protein; Regulatory component of the cyclin D1-CDK4 (DC) complex that phosphorylates and inhibits members of the retinoblastoma (RB) protein family including RB1 and regulates the cell-cycle during G(1)/S transition. Phosphorylation of RB1 allows dissociation of the transcription factor E2F from the RB/E2F complex and the subsequent transcription of E2F target genes which are responsible for the progression through the G(1) phase. Hypophosphorylates RB1 in early G(1) phase. Cyclin D-CDK4 complexes are major integrators of various mitogenic and antimitogenic signals.	3.26	1.55
**GO:0042127 Regulation of cell population proliferation**			
**Symbol**	**Description**	**Z-score**	
SMAD2	Mothers against decapentaplegic homolog 2; Receptor-regulated SMAD (R-SMAD) that is an intracellular signal transducer and transcriptional modulator activated by TGF-beta (transforming growth factor) and activin type 1 receptor kinases. Binds the TRE element in the promoter region of many genes that are regulated by TGF-beta and, on formation of the SMAD2/SMAD4 complex, activates transcription. May act as a tumor suppressor in colorectal carcinoma.	1.57	2.52
IGF2	Insulin-like growth factor 2 (somatomedin A); The insulin-like growth factors possess growth-promoting activity. Major fetal growth hormone in mammals. Plays a key role in regulating fetoplacental development. IGF-II is influenced by placental lactogen. Also involved in tissue differentiation.	2.16	2.25
SMARCB1	SWI/SNF related, matrix associated, actin dependent regulator of chromatin, subfamily b, member 1; Core component of the BAF (hSWI/SNF) complex. This ATP- dependent chromatin-remodeling complex plays important roles in cell proliferation and differentiation, in cellular antiviral activities and inhibition of tumor formation. The BAF complex is able to create a stable, altered form of chromatin that constrains fewer negative supercoils than normal	1.91	2.18
PLAC8	Placenta-specific gene 8 protein; Placenta specific 8	2.30	1.95
CD74	CD74 molecule, major histocompatibility complex, class II invariant chain; Plays a critical role in MHC class II antigen processing by stabilizing peptide-free class II alpha/beta heterodimers in a complex soon after their synthesis and directing transport of the complex from the endoplasmic reticulum to the endosomal/lysosomal system where the antigen processing and binding of antigenic peptides to MHC class II takes place. Serves as cell surface receptor for the cytokine MIF; CD molecules	3.00	1.86
KDM5B	Retinoblastoma-binding protein 2 homolog 1; Histone demethylase that demethylates 'Lys-4' of histone H3, thereby playing a central role in histone code. Does not demethylate histone H3 'Lys-9' or H3 'Lys-27'. Demethylates trimethylated, dimethylated and monomethylated H3 'Lys-4'. Acts as a transcriptional corepressor for FOXG1B and PAX9.	1.87	1.83

ASPH	Aspartyl/asparaginyl beta-hydroxylase; Isoform 1: specifically hydroxylates an Asp or Asn residue in certain epidermal growth factor-like (EGF) domains of a number of proteins; Belongs to the aspartyl/asparaginyl beta-hydroxylase family.	1.63	1.76
APC	Adenomatous polyposis coli protein; Tumor suppressor. Promotes rapid degradation of CTNNB1 and participates in Wnt signaling as a negative regulator. APC activity is correlated with its phosphorylation state.	1.54	1.75
CDH13	Truncated cadherin; Cadherins are calcium-dependent cell adhesion proteins. They preferentially interact with themselves in a homophilic manner in connecting cells; cadherins may thus contribute to the sorting of heterogeneous cell types. May act as a negative regulator of neural cell growth.	2.91	1.72
FGF8	Fibroblast growth factor 8 (androgen-induced); Plays an important role in the regulation of embryonic development, cell proliferation, cell differentiation and cell migration. Required for normal brain, eye, ear, and limb development during embryogenesis. Required for normal development of the gonadotropin-releasing hormone (GnRH) neuronal system.	1.82	1.69
MED1	Thyroid hormone receptor-associated protein complex 220 kDa component; Component of the Mediator complex, a coactivator involved in the regulated transcription of nearly all RNA polymerase II-dependent genes. Mediator functions as a bridge to convey information from gene-specific regulatory proteins to the basal RNA polymerase II transcription machinery. Mediator is recruited to promoters by direct interactions with regulatory proteins and serves as a scaffold for the assembly of a functional preinitiation complex with RNA polymerase II and the general transcription factors. Acts as a coactivator for GATA1-mediated transcriptional activation during erythroid differentiation of K562 erythroleukemia cells.	1.96	1.68
PGF	Placental growth factor; Growth factor active in angiogenesis and endothelial cell growth, stimulating their proliferation and migration. It binds to the receptor FLT1/VEGFR-1. Isoform PlGF-2 binds NRP1/neuropilin-1 and NRP2/neuropilin-2 in a heparin-dependent manner.	1.97	1.62
TNFRSF4	TAX transcriptionally-activated glycoprotein 1 receptor; Receptor for TNFSF4/OX40L/GP34. Is a costimulatory molecule implicated in long-term T-cell immunity; CD molecules	2.05	1.61
CCND1	B-cell lymphoma 1 protein; Regulatory component of the cyclin D1-CDK4 (DC) complex that phosphorylates and inhibits members of the retinoblastoma (RB) protein family including RB1 and regulates the cell-cycle during G(1)/S transition. Phosphorylation of RB1 allows dissociation of the transcription factor E2F from the RB/E2F complex and the subsequent transcription of E2F target genes which are responsible for the progression through the G(1) phase. Hypophosphorylates RB1 in early G(1) phase. Cyclin D-CDK4 complexes are major integrators of various mitogenenic and antimitogenic signals.	3.26	1.55
**GO:007162 Negative regulation of cell adhesion**			

Symbol	Description	Z-score	
HFE	Hereditary hemochromatosis protein: Binds to transferrin receptor (TFR) and reduces its affinity for iron-loaded transferrin; Belongs to the MHC class I family.	2.53	2.52
ANGPT2	Angiopoietin 2; Binds to TEK/TIE2, competing for the ANGPT1 binding site, and modulating ANGPT1 signaling. Can induce tyrosine phosphorylation of TEK/TIE2 in the absence of ANGPT1. In the absence of angiogenic inducers, such as VEGF, ANGPT2-mediated loosening of cell-matrix contacts may induce endothelial cell apoptosis with consequent vascular regression.	1.86	1.93
CD74	CD74 molecule, major histocompatibility complex, class II invariant chain; plays a critical role in MHC class II antigen processing by stabilizing peptide-free class II alpha/beta heterodimers in a complex soon after their synthesis and directing transport of the complex from the endoplasmic reticulum to the endosomal/lysosomal system where the antigen processing and binding of antigenic peptides to MHC class II takes place. Serves as cell surface receptor for the cytokine MIF; CD molecules	3.00	1.86
CDH13	Truncated cadherin; Cadherins are calcium-dependent cell adhesion proteins. They preferentially interact with themselves in a homophilic manner in connecting cells; cadherins may thus contribute to the sorting of heterogeneous cell types. May act as a negative regulator of neural cell growth.	2.91	1.72
**Down-regulated transcripts in SHARED network**			
**GO:0042493 Response to drug**			
**Symbol**	**Description**	**Z-score**	
CREB1	Cyclic AMP-responsive element-binding protein 1; Phosphorylation-dependent transcription factor that stimulates transcription upon binding to the DNA cAMP response element (CRE), a sequence present in many viral and cellular promoters. Transcription activation is enhanced by the TORC coactivators which act independently of Ser-133 phosphorylation. Involved in different cellular processes including the synchronization of circadian rhythmicity and the differentiation of adipose cells; Basic leucine zipper proteins	-3.36	-2.30
ANXA1	Phospholipase A2 inhibitory protein; Plays important roles in the innate immune response as effector of glucocorticoid-mediated responses and regulator of the inflammatory process. Has anti-inflammatory activity. Plays a role in glucocorticoid-mediated down- regulation of the early phase of the inflammatory response (By similarity).	-2.52	-2.02
NR4A3	Nuclear receptor subfamily 4, group A, member 3; Transcriptional activator that binds to regulatory elements in promoter regions in a cell- and response element (target)-specific manner. Induces gene expression by binding as monomers to the NR4A1 response element (NBRE) 5'-AAAAGGTCA-3' site and as homodimers to the Nur response element (NurRE) site in the promoter of their regulated target genes (By similarity). Plays a role in the regulation of proliferation, survival, and differentiation of many different cell types and also in metabolism and inflammation.	-2.21	-1.88

XRCC5	ATP-dependent DNA helicase II 80 kDa subunit; Single-stranded DNA-dependent ATP-dependent helicase. Has a role in chromosome translocation. The DNA helicase II complex binds preferentially to fork-like ends of double-stranded DNA in a cell cycle-dependent manner.	-1.56	-1.60
CASP8	Caspase 8, apoptosis-related cysteine peptidase; Most upstream protease of the activation cascade of caspases responsible for the TNFRSF6/FAS mediated and TNFRSF1A induced cell death. Binding to the adapter molecule FADD recruits it to either receptor. The resulting aggregate called death- inducing signaling complex (DISC) performs CASP8 proteolytic activation. The active dimeric enzyme is then liberated from the DISC and free to activate downstream apoptotic proteases.	-1.71	-1.58
NFKBIA	Nuclear factor of kappa light polypeptide gene enhancer in B-cells inhibitor, alpha; Inhibits the activity of dimeric NF-kappa-B/REL complexes by trapping REL dimers in the cytoplasm through masking of their nuclear localization signals. On cellular stimulation by immune and proinflammatory responses, becomes phosphorylated promoting ubiquitination and degradation, enabling the dimeric RELA to translocate to the nucleus and activate transcription.	-3.06	-1.58
LYN	V-yes-1 Yamaguchi sarcoma viral related oncogene homolog; Non-receptor tyrosine-protein kinase that transmits signals from cell surface receptors and plays an important role in the regulation of innate and adaptive immune responses, hematopoiesis, responses to growth factors and cytokines, integrin signaling, but also responses to DNA damage and genotoxic agents.	-1.50	-1.58
**GO:0012501 Programmed cell death**			
**Symbol**	**Description**	**Z-score**	
DAP3	Mitochondrial small ribosomal subunit protein mS29; Involved in mediating interferon-gamma-induced cell death; Belongs to the mitochondrion-specific ribosomal protein mS29 family.	-2.35	-2.30
CTSV	Cathepsin L2; Cysteine protease. May have an important role in corneal physiology; Belongs to the peptidase C1 family.	-3.44	-2.27
CASP10	FAS-associated death domain protein interleukin-1B-converting enzyme 2; Involved in the activation cascade of caspases responsible for apoptosis execution. Recruited to both Fas- and TNFR-1 receptors in a FADD dependent manner. May participate in the granzyme B apoptotic pathways.	-2.49	-1.90
CASP8	Caspase 8, apoptosis-related cysteine peptidase; Most upstream protease of the activation cascade of caspases responsible for the TNFRSF6/FAS mediated and TNFRSF1A induced cell death. Binding to the adapter molecule FADD recruits it to either receptor. The resulting aggregate called death- inducing signaling complex (DISC) performs CASP8 proteolytic activation.	-1.71	-1.58
NFKBIA	Nuclear factor of kappa light polypeptide gene enhancer in B-cells inhibitor, alpha; Inhibits the activity of dimeric NF-kappa-B/REL complexes by trapping REL dimers in the cytoplasm through masking of their nuclear localization signals. On cellular stimulation by immune and proinflammatory responses, becomes phosphorylated promoting	-3.06	-1.58

	ubiquitination and degradation, enabling the dimeric RELA to translocate to the nucleus and activate transcription.		
HIC1	Zinc finger and BTB domain-containing protein 29; Transcriptional repressor. Recognizes and binds to the consensus sequence '5-[CG]NG[CG]GGGCA[CA]CC-3'. May act as a tumor suppressor. May be involved in development of head, face, limbs, and ventral body wall.	-1.63	-1.52
**GO:0045785 Positive regulation of cell adhesion**			
**Symbol**	**Description**	**Z-score**	
ANXA1	Phospholipase A2 inhibitory protein; Plays important roles in the innate immune response as effector of glucocorticoid-mediated responses and regulator of the inflammatory process. Has anti-inflammatory activity. Plays a role in glucocorticoid-mediated down- regulation of the early phase of the inflammatory response (By similarity).	-2.52	-2.02
NR4A3	Nuclear receptor subfamily 4, group A, member 3; Transcriptional activator that binds to regulatory elements in promoter regions in a cell- and response element (target)-specific manner. Induces gene expression by binding as monomers to the NR4A1 response element (NBRE) 5'-AAAAGGTCA-3' site and as homodimers to the Nur response element (NurRE) site in the promoter of their regulated target genes (By similarity). Plays a role in the regulation of proliferation, survival, and differentiation of many different cell types and also in metabolism and inflammation.	-2.21	-1.88
CXCL12	Pre-B cell growth-stimulating factor; Chemoattractant active on T-lymphocytes, monocytes, but not neutrophils. Activates the C-X-C chemokine receptor CXCR4 to induce a rapid and transient rise in the level of intracellular calcium ions and chemotaxis. Also binds to atypical chemokine receptor ACKR3, which activates the beta-arrestin pathway and acts as a scavenger receptor for SDF-1. SDF-1-beta(3-72) and SDF-1-alpha(3-67) show a reduced chemotactic activity.	-1.52	-1.87
LYN	V-yes-1 Yamaguchi sarcoma viral related oncogene homolog; Non-receptor tyrosine-protein kinase that transmits signals from cell surface receptors and plays an important role in the regulation of innate and adaptive immune responses, hematopoiesis, responses to growth factors and cytokines, integrin signaling, but also responses to DNA damage and genotoxic agents.	-1.50	-1.58

Supplementary table 4. Transcript changes after apyrase treatment in AGS cells

Up- regulated transcripts		
GO:0045930 Negative regulation of mitotic cell cycle		FDR: 3.1E-4
**Symbol**	**Description**	**Z-score**
**ATM**	Ataxia telangiectasia mutated; Serine/threonine protein kinase which activates checkpoint signaling upon double strand breaks (DSBs), apoptosis and genotoxic stresses, thereby acting as a DNA damage sensor. Phosphorylates 'Ser-139' of histone variant H2AX/H2AFX at double strand breaks (DSBs), thereby regulating DNA damage response mechanism. Also involved in signal transduction and cell cycle control. May function as a tumor suppressor.	2.50
**MSH2**	DNA mismatch repair protein Msh2; Component of the post-replicative DNA mismatch repair system (MMR). Forms two different heterodimers: MutS alpha (MSH2- MSH6 heterodimer) and MutS beta (MSH2-MSH3 heterodimer) which binds to DNA mismatches thereby initiating DNA repair. When bound, heterodimers bend the DNA helix and shields approximately 20 base pairs. In melanocytes may modulate both UV-B-induced cell cycle regulation and apoptosis.	2.29
**GLTSCR2**	Glioma tumor suppressor candidate region gene 2 protein; Nucleolar protein which is involved in the integration of the 5S RNP into the ribosomal large subunit during ribosome biogenesis. Also functions as a nucleolar sensor that regulates the activation of p53/TP53 in response to ribosome biogenesis perturbation, DNA damage and other stress conditions. It may also positively regulate the function of p53/TP53 in cell cycle arrest and apoptosis through direct interaction, preventing its MDM2-dependent ubiquitin-mediated proteasomal degradation. Originally identified as a tumor suppressor, it may also play a role in cell proliferation and apoptosis by positively regulating the stability of PTEN, thereby antagonizing the PI3K-AKT/PKB signaling pathway. May also inhibit cell proliferation and increase apoptosis through its interaction with NF2.	2.28
**MAD2L2**	Mitotic spindle assembly checkpoint protein MAD2B; Adapter protein able to interact with different proteins and involved in different biological processes. Mediates the interaction between the error-prone DNA polymerase zeta catalytic subunit REV3L and the inserter polymerase REV1, thereby mediating the second polymerase switching in translesion DNA synthesis. Inhibits the FZR1- and probably CDC20-mediated activation of the anaphase promoting complex APC thereby regulating progression through the cell cycle. Regulates TCF7L2-mediated gene transcription and may play a role in epithelial-mesenchymal transdifferentiation.	1.97

**CDKN1B**	Cyclin-dependent kinase inhibitor 1B (p27, Kip1); Important regulator of cell cycle progression. Inhibits the kinase activity of CDK2 bound to cyclin A but has little inhibitory activity on CDK2 bound to SPDYA. Involved in G1 arrest.	1.95
**GAS1**	Growth arrest-specific protein 1; Specific growth arrest protein involved in growth suppression. Blocks entry to S phase. Prevents cycling of normal and transformed cells.	1.87
**CDK2AP2**	Cyclin-dependent kinase 2 associated protein 2; Plays a role in regulating the self-renewal of embryonic stem cells (ESCs) and in maintaining cell survival during terminal differentiation of ESCs. Regulates microtubule organization of metaphase II oocytes (By similarity). Inhibits cell cycle G1/S phase transition by repressing CDK2 expression and activation; represses CDK2 activation by inhibiting its interaction with cyclin E and A.	1.84
**SMAD3**	Mothers against decapentaplegic homolog 3; Receptor-regulated SMAD (R-SMAD) that is an intracellular signal transducer and transcriptional modulator activated by TGF-beta (transforming growth factor) and activin type 1 receptor kinases. Has an inhibitory effect on wound healing probably by modulating both growth and migration of primary keratinocytes and by altering the TGF- mediated chemotaxis of monocytes.	1.72
**PRCC**	Papillary renal cell carcinoma translocation-associated gene protein; May regulate cell cycle progression through interaction with MAD2L2; Spliceosomal Bact complex	1.63
**GO:0045785 Positive regulation of cell adhesion**		FDR: 0.0076
**Symbol**	**Description**	**Z-score**
**TFE3**	Transcription factor binding to IGHM enhancer 3; Transcription factor that specifically recognizes and binds E-box sequences (5'-CANNTG-3'). Efficient DNA-binding requires dimerization with itself or with another MiT/TFE family member such as TFEB or MITF. In association with TFEB, activates the expression of CD40L in T-cells, thereby playing a role in T- cell-dependent antibody responses in activated CD4(+) T-cells and thymus-dependent humoral immunity.	2.46
**CD74**	CD74 molecule, major histocompatibility complex, class II invariant chain; Plays a critical role in MHC class II antigen processing by stabilizing peptide-free class II alpha/beta heterodimers in a complex soon after their synthesis and directing transport of the complex from the endoplasmic reticulum to the endosomal/lysosomal system where the antigen processing and binding of antigenic peptides to MHC class II takes place. Serves as cell surface receptor for the cytokine MIF; CD molecules	2.44
**RREB1**	Zinc finger motif enhancer-binding protein 1; Transcription factor that binds specifically to the RAS- responsive elements (RRE) of gene promoters. May be involved in Ras/Raf-mediated cell differentiation by enhancing calcitonin expression. Represses the angiotensinogen gene.	2.42

**ALOX15**	Arachidonate 12-lipoxygenase, leukocyte-type; Non-heme iron-containing dioxygenase that catalyzes the stereo-specific peroxidation of free and esterified polyunsaturated fatty acids generating a spectrum of bioactive lipid mediators. Converts arachidonic acid into 12- hydroperoxyeicosatetraenoic acid/12-HPETE and 15- hydroperoxyeicosatetraenoic acid/15-HPETE. Also converts linoleic acid to 13-hydroperoxyoctadecadienoic acid. In parallel, may regulate actin polymerization which is crucial for several biological processes.	2.20
**PAK1**	P21 protein (Cdc42/Rac)-activated kinase 1; Protein kinase involved in intracellular signaling pathways downstream of integrins and receptor-type kinases that plays an important role in cytoskeleton dynamics, in cell adhesion, migration, proliferation, apoptosis, mitosis, and in vesicle-mediated transport processes. Can directly phosphorylate BAD and protects cells against apoptosis. Involved in the reorganization of the actin cytoskeleton, actin stress fibers and of focal adhesion complexes, increasing its efficiency in chemokine uptake and degradation.	2.07
**EGFL6**	MAM and EGF domains-containing gene protein; May bind integrin alpha-8/beta-1 and play a role in hair follicle morphogenesis. Promotes matrix assembly (By similarity); Belongs to the nephronectin family.	1.86
**SMAD3**	Mothers against decapentaplegic homolog 3; Receptor-regulated SMAD (R-SMAD) that is an intracellular signal transducer and transcriptional modulator activated by TGF-beta (transforming growth factor) and activin type 1 receptor kinases. Has an inhibitory effect on wound healing probably by modulating both growth and migration of primary keratinocytes and by altering the TGF- mediated chemotaxis of monocytes.	1.72
**MAP3K8**	Mitogen-activated protein kinase kinase kinase 8; Required for lipopolysaccharide (LPS)-induced, TLR4- mediated activation of the MAPK/ERK pathway in macrophages, thus being critical for production of the proinflammatory cytokine TNF- alpha (TNF) during immune responses. Involved in the regulation of T-helper cell differentiation and IFNG expression in T-cells. Involved in mediating host resistance to bacterial infection through negative regulation of type I interferon (IFN) production.	1.66
**Down-regulated transcripts**		
**GO: 0030335 Positive regulation of cell migration**		FDR: 0.0022
**Symbol**	**Description**	**Z-score**
**NR4A3**	Nuclear receptor subfamily 4, group A, member 3; Transcriptional activator that binds to regulatory elements in promoter regions in a cell- and response element (target)-specific manner. Plays a role in the regulation of proliferation, survival, and differentiation of many different cell types and also in metabolism and inflammation.	-3.04
**FADD**	FAS-associating death domain-containing protein; Apoptotic adaptor molecule that recruits caspase-8 or caspase-10 to the	-2.26

	activated Fas (CD95) or TNFR-1 receptors. The resulting aggregate called the death-inducing signaling complex (DISC) performs caspase-8 proteolytic activation. Active caspase-8 initiates the subsequent cascade of caspases mediating apoptosis.	
**IGFBP5**	Insulin-like growth factor binding protein 5; IGF-binding proteins prolong the half-life of the IGFs and have been shown to either inhibit or stimulate the growth promoting effects of the IGFs on cell culture. They alter the interaction of IGFs with their cell surface receptors.	-2.03
**TEK**	Tyrosine kinase with Ig and EGF homology domains-2; acts as cell-surface receptor for ANGPT1, ANGPT2 and ANGPT4 and regulates angiogenesis, endothelial cell survival, proliferation, migration, adhesion, and cell spreading, reorganization of the actin cytoskeleton, but also maintenance of vascular quiescence.	-1.87
**SRC**	SRC proto-oncogene, non-receptor tyrosine kinase; Non-receptor protein tyrosine kinase which is activated following engagement of many different classes of cellular receptors. Participates in signaling pathways that control a diverse spectrum of biological activities including gene transcription, immune response, cell adhesion, cell cycle progression, apoptosis, migration, and transformation.	-1.74
**LYN**	V-yes-1 Yamaguchi sarcoma viral related oncogene homolog; Non-receptor tyrosine-protein kinase that transmits signals from cell surface receptors and plays an important role in the regulation of innate and adaptive immune responses, hematopoiesis, responses to growth factors and cytokines, integrin signaling, but also responses to DNA damage and genotoxic agents. Regulates cell proliferation, survival, differentiation, migration, adhesion, degranulation, and cytokine release.	-1.64
**ABL1**	ABL proto-oncogene 1, non-receptor tyrosine kinase; plays a role in many key processes linked to cell growth and survival such as cytoskeleton remodeling in response to extracellular stimuli, cell motility and adhesion, receptor endocytosis, autophagy, DNA damage response and apoptosis. Phosphorylation of WASF3 is critical for the stimulation of lamellipodia formation and cell migration. Involved in the regulation of cell adhesion and motility through phosphorylation of key regulators of these processes such as BCAR1, CRK, CRKL, DOK1, EFS or NEDD9.	-1.63
**CXCL10**	10 kDa interferon gamma-induced protein; Chemotactic for monocytes and T-lymphocytes. Binds to CXCR3; Belongs to the intercrine alpha (chemokine CxC) family.	-1.62
**PTGS2**	Prostaglandin-endoperoxide synthase 2 (prostaglandin G/H synthase and cyclooxygenase); Converts arachidonate to prostaglandin H2 (PGH2), a committed step in prostanoid synthesis. Constitutively expressed in some tissues in physiological conditions, such as the endothelium, kidney, and brain, and in pathological conditions, such as in cancer. In cancer cells, PTGS2 is a key step in the production of prostaglandin E2 (PGE2), which	-1.57

	plays important roles in modulating motility, proliferation and resistance to apoptosis.	
**EPHA1**	Erythropoietin-producing hepatoma receptor; Receptor tyrosine kinase which binds promiscuously membrane-bound ephrin-A family ligands residing on adjacent cells, leading to contact-dependent bidirectional signaling into neighboring cells. T. Plays also a role in angiogenesis and regulates cell proliferation. May play a role in apoptosis.	-1.54
	**GO:0043410 Positive regulation of MAPK cascade**	FDR: 4.4E-4
**Symbol**	**Description**	**Z-score**
**TRAF4**	Cysteine-rich domain associated with RING and Traf domains protein 1; Adapter protein and signal transducer that links members of the tumor necrosis factor receptor (TNFR) family to different signaling pathways. Plays a role in the activation of NF-kappa-B and JNK, and in the regulation of cell survival and apoptosis. Required for activation of RPS6KB1 in response to TNF signaling. Modulates TRAF6 functions.	-3.53
**FZD4**	Frizzled class receptor 4; Receptor for Wnt proteins. Plays a critical role in retinal vascularization by acting as a receptor for Wnt proteins and norrin (NDP). May be involved in transduction and intercellular transmission of polarity information during tissue morphogenesis and/or in differentiated tissues; CD molecules	-2.58
**MAP4K2**	Mitogen-activated protein kinase kinase kinase kinase 2; Serine/threonine-protein kinase which acts as an essential component of the MAP kinase signal transduction pathway. Is an upstream activator of the stress-activated protein kinase/c-Jun N-terminal kinase (SAP/JNK) signaling pathway and to a lesser extent of the p38 MAPKs signaling pathway. Required for the efficient activation of JNKs by TRAF6-dependent stimuli. May play a role in the regulation of vesicle targeting or fusion.	-2.29
**BRAF**	v-Raf murine sarcoma viral oncogene homolog B1; Protein kinase involved in the transduction of mitogenic signals from the cell membrane to the nucleus. May play a role in the postsynaptic responses of hippocampal neuron. Phosphorylates MAP2K1, and thereby contributes to the MAP kinase signal transduction pathway.	-1.97
**TNFRSF11A**	Tumor necrosis factor receptor superfamily, member 11a, NFKB activator; Receptor for TNFSF11/RANKL/TRANCE/OPGL; essential for RANKL-mediated osteoclastogenesis. Involved in the regulation of interactions between T-cells and dendritic cells; CD molecules	-1.95
**TEK**	Tyrosine kinase with Ig and EGF homology domains-2; acts as cell-surface receptor for ANGPT1, ANGPT2 and ANGPT4 and regulates angiogenesis, endothelial cell survival, proliferation, migration, adhesion, and cell spreading, reorganization of the actin cytoskeleton, but also maintenance of vascular quiescence.	-1.87
**CD36**	CD36 molecule (thrombospondin receptor); Multifunctional glycoprotein that acts as receptor for a broad range of ligands such	-1.76

	as like thrombospondin, fibronectin, collagen or amyloid-beta as well as oxidized low-density lipoprotein (oxLDL), anionic phospholipids, long-chain fatty acids and bacterial diacylated lipopeptides. Cellular responses to these ligands are involved in angiogenesis, inflammatory response, fatty acid metabolism, taste and dietary fat processing in the intestine (Probable).	
**HLA-DRB1**	HLA class II histocompatibility antigen, DRB1-15 beta chain; Binds peptides derived from antigens that access the endocytic route of antigen presenting cells (APC) and presents them on the cell surface for recognition by the CD4 T-cells. The peptide binding cleft accommodates peptides of 10-30 residues. The peptides presented by MHC class II molecules are generated mostly by degradation of proteins that access the endocytic route, where they are processed by lysosomal proteases and other hydrolases. Exogenous antigens that have been endocytosed by the APC are thus readily available for presentation via MHC II molecules, and for this reason this antigen presentation pathway is usually referred to as exogenous. As membrane proteins on their way to degradation in lysosomes as part of their normal turn-over are also contained in the endosomal/lysosomal compartments, exogenous antigens must compete with those derived from endogenous components. Autophagy is also a source of endogenous peptides, autophagosomes constitutively fuse with MHC class II loading compartments. In addition to APCs, other cells of the gastrointestinal tract, such as epithelial cells, express MHC class II molecules and CD74 and act as APCs, which is an unusual trait of the GI tract. To produce a MHC class II molecule that presents an antigen, three MHC class II molecules (heterodimers of an alpha and a beta chain) associate with a CD74 trimer in the ER to form a heterononamer. Soon after the entry of this complex into the endosomal/lysosomal system where antigen processing occurs, CD74 undergoes a sequential degradation by various proteases, including CTSS and CTSL, leaving a small fragment termed CLIP (class-II-associated invariant chain peptide). The removal of CLIP is facilitated by HLA-DM via direct binding to the alpha-beta-CLIP complex so that CLIP is released. HLA-DM stabilizes MHC class II molecules until primary high affinity antigenic peptides are bound. The MHC II molecule bound to a peptide is then transported to the cell membrane surface. In B-cells, the interaction between HLA-DM and MHC class II molecules is regulated by HLA-DO. Primary dendritic cells (DCs) also to express HLA-DO. Lysosomal microenvironment has been implicated in the regulation of antigen loading into MHC II molecules, increased acidification produces increased proteolysis and efficient peptide loading.	-1.74
**SRC**	SRC proto-oncogene, non-receptor tyrosine kinase; Non-receptor protein tyrosine kinase which is activated following engagement of many different classes of cellular receptors. Participates in signaling pathways that control a diverse spectrum of biological	-1.74

	activities including gene transcription, immune response, cell adhesion, cell cycle progression, apoptosis, migration, and transformation.	
**DFNA5**	Inversely correlated with estrogen receptor expression 1; Plays a role in the TP53-regulated cellular response to DNA damage probably by cooperating with TP53.	-1.65
**ABL1**	ABL proto-oncogene 1, non-receptor tyrosine kinase; plays a role in many key processes linked to cell growth and survival such as cytoskeleton remodeling in response to extracellular stimuli, cell motility and adhesion, receptor endocytosis, autophagy, DNA damage response and apoptosis. Phosphorylation of WASF3 is critical for the stimulation of lamellipodia formation and cell migration. Involved in the regulation of cell adhesion and motility through phosphorylation of key regulators of these processes such as BCAR1, CRK, CRKL, DOK1, EFS or NEDD9.	-1.63
**MAPKAPK3**	Mitogen-activated protein kinase-activated protein kinase 3; Stress-activated serine/threonine-protein kinase involved in cytokines production, endocytosis, cell migration, chromatin remodeling and transcriptional regulation. Involved in inflammatory response by regulating tumor necrosis factor (TNF) and IL6 production post-transcriptionally. Also acts as a modulator of Polycomb-mediated repression.	-1.60
